# Development of naringenin-*O*-alkylamine derivatives as multifunctional agents for the treatment of Alzheimer’s disease

**DOI:** 10.1080/14756366.2022.2041627

**Published:** 2022-02-22

**Authors:** Jing Yang, Yi Zhou, Yujuan Ban, Jing Mi, Ying He, Xinjuan Li, Zhengwei Liu, Keren Wang, Gaofeng Zhu, Wenmin Liu, Zhenghuai Tan, Zhipei Sang

**Affiliations:** a College of Chemistry and Pharmaceutical Engineering, Nanyang Normal University, Nanyang, China; b State Key Laboratory of Functions and Applications of Medicinal Plants, Guizhou Provincial Engineering Technology Research Center for Chemical Drug R&D, Guizhou Medical University, Guiyang, China; c Institute of Traditional Chinese Medicine Pharmacology and Toxicology, Sichuan Academy of Chinese Medicine Sciences, Chengdu, China

**Keywords:** Alzheimer’s disease, naringenin-*O*-alkylamine derivatives, multifunctional agents, scopolamine-induced mice memory impairment

## Abstract

In this study, a series of naringenin-*O*-alkylamine derivatives were designed and obtained by introducing an alkylamine fragment into the naringenin skeleton. The *in vitro* biological activity results revealed that compounds **5f** and **7k** showed good antioxidant activity with ORAC values of 2.3*eq* and 1.2*eq*, respectively. Compounds **5f** and **7k** were reversible and excellent *hu*AChE inhibitors with IC_50_ values of 0.91 μM and 0.57 μM, respectively. Moreover, compounds **5f** and **7k** could inhibit self-induced A*β*_1–42_ aggregation with 62.1% and 43.8% inhibition rate, respectively, and significantly inhibited *hu*AChE-A*β*_1–40_ aggregation with 51.7% and 43.4% inhibition rate, respectively. In addition, compounds **5f** and **7k** were selective metal chelators and remarkably inhibited Cu^2+^-induced A*β*_1–42_ aggregation with 73.5% and 68.7% inhibition rates, respectively. Furthermore, compounds **5f** and **7k** could cross **the** blood-brain barrier *in vitro* and displayed good neuroprotective effects and anti-inflammatory properties. Further investigation showed that compound **5f** did not show obvious hepatotoxicity and displayed a good hepatoprotective effect by its antioxidant activity. The *in vivo* study displayed that compound **5f** significantly improved scopolamine-induced mice memory impairment. Therefore, compound **5f** was a potential multifunctional candidate for the treatment of AD.

## 1. Introduction

Alzheimer’s disease (AD), accounting for about 70% of all dementia cases, is a chronic, progressive neurodegenerative brain disease in elderly people. According to the World Alzheimer Report, there are more than 50 million people living with dementia worldwide and the figure of AD patients will triple by 2050^1^. Accordingly, AD poses a great problem for global health. Despite many scientific efforts, the mechanism underlying the aetiology of AD is not exactly explained due to its complex and multifactorial characteristics. However, several factors including deficits of acetylcholine (ACh), amyloid-*β* (A*β*) oligomer deposits, elevated oxidative stress, dyshomeostasis of biometals and neuroinflammation, have been considered as crucial roles in AD onset and progression^2^.

The cholinergic hypothesis states that the degeneration of cholinergic neurons and the associated loss of cholinergic neurotransmission in the cerebral cortex are responsible for the deterioration of cognitive function observed in the brain of AD patients. There are two types of cholinesterases (ChEs) that can catalyse the hydrolysis of ACh, namely acetylcholinesterase (AChE) and butyrylcholinesterase (BuChE). AChE is mainly distributed in the nerve tissues like white matter and grey matter, while BuChE is widely spread in the plasma, liver and muscle tissues^3^^,^^4^. Therefore, selective inhibition of AChE would be beneficial to the treatment of AD, such as the approved anti-AD drugs, donepezil, galanthamine and tacrine^3^. In addition, evidence display that AChE can accelerate the aggregation of A*β* through peripheral anion site (PAS), producing stable AChE-A*β* complexes, which present more neurotoxicity than single A*β* peptide^5^. Moreover, A*β* is generated by the proteolytic processing of amyloid precursor protein (APP), including A*β*_1–40_ *and* A*β*_1–42_. A*β*_1–42_ is more likely to aggregate and form the A*β* oligomer, which triggers neuroinflammation, tau-related neurofibrillary tangles and neuronal degeneration, leading to AD^6^. Therefore, selective inhibition of AChE and the reduction of A*β*_1–42_ are promising therapeutic strategies for the treatment of AD.

Accumulation of evidence exhibits that neuroinflammation acts as a fundamental role in AD patients, which is related to microglia, astrocytes and inflammatory factors. Microglia activated by A*β* stimulation produce inflammatory factors and free radicals, such as tumour necrosis factor (TNF-α), interferon γ (INF-γ), ROS and so on^7^. These neurotoxic substances induce neuroinflammation and neurons damage, and increase A*β* deposition, leading to cognitive dysfunction and neuron loss. Therefore, the anti-inflammatory property might be a potent approach for treating AD.

Numerous studies show that oxidative damage is one of the earliest features of AD. The oxidative homeostasis imbalance in AD brain leads to the abnormal generation of reactive oxygen species (ROS)^8^. ROS can damage lipids, proteins and nucleic acids, and further accelerates the formation of amyloid and inflammation. Furthermore, the bio-metals (Cu^2+^, Zn^2+^, Al^3+^, Fe^2+^) have been verified to be related to AD, the concentration of bio-metals in the brain of AD patients is 3–7 times higher than healthy individuals. The metal dyshomeostasis may potentially cause neurotoxic effects by affecting A*β* aggregation and oxidative stress^9^. Thus, antioxidant activity or metal chelators could be beneficial to the treatment of AD.

Given that AD is a complex disease with multifactorial pathological nature, the multi-target-directed ligand strategy (MTDLs) to develop novel small molecules which can hit two or more AD-relevant complementary targets in the progression of AD, has drawn considerable attention for their advancement in the treatment of AD^10–12^. In particular, the multifunctional agents with AChE inhibitory potency present the ability to tacking the progression of AD while relieving symptoms^13^.

**Figure 1.**
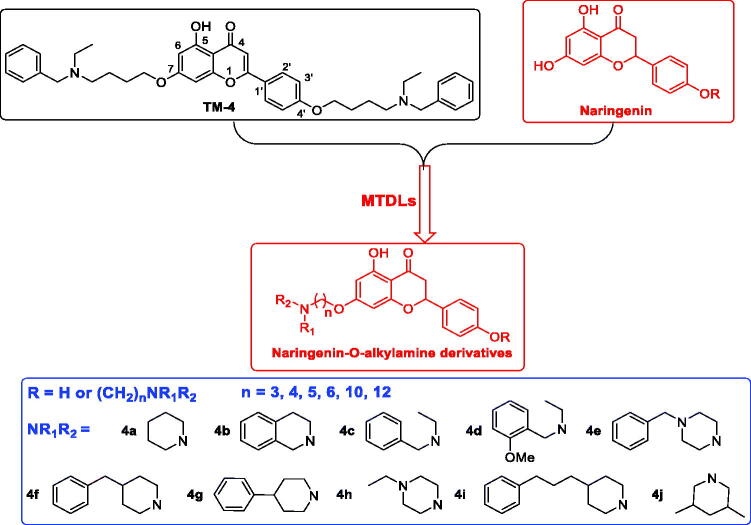
The design strategy of naringenin-*O*-alkylamine derivatives.

Naringenin, 5,7-dihydroxy-2–(4-hydroxyphenyl)-2,3-dihydrochromen-4-one, is a primary flavanone, present in orange, grape fruit, tangerine, raw lemon peels and raw lime peel. Naringenin possesses significant antioxidant and anti-inflammatory activities, which are beneficial to the treatment of AD^14^. Studies show that naringenin could improve cholinergic function *in vivo* due to its antioxidant property, and it attenuates A*β*-induced impairment of learning and memory^15^. However, the hydroxy of naringenin limited its clinical use as an anti-AD drug due to merely 15% oral bioavailability^14^. In order to improve bioavailability and develop naringenin as multifunctional agents anti-AD, naringenin carbamate derivatives have been designed and evaluated, however, these derivatives were selective BuChE inhibitors^16^. Donepezil is a selective AChE inhibitor approved by FDA, and 1-benzylpiperidine is the key pharmacophore, based on this, *O*-alkylamine fragments have been developed as effective AChE inhibition fragments, our group has developed natural flavonoids as multifunctional agents by introducing *O*-alkylamine fragments^17–20^. In our previous work, we had developed a series of apigenin/naringenin/genistein-*O*-alkylamine derivatives with the length of methylene was 4 based on MTDLs, and the results revealed that apigenin-*O*-alkylamine derivative candidate **TM-4** was a promising multifunctional agent for treating AD^20^. In order to further develop naringenin skeleton as multifunctional agents, we plan to introduce different tertiary amino groups into the naringenin skeleton by changing the length of methylene to obtain novel naringenin-*O*-alkylamine derivatives (Figure 1), moreover, apigenin-*O*-alkylamine derivatives were also synthesised to explore the structure-activity-relationship of naringenin-*O*-alkylamine derivatives, hoping these derivatives possess multifunctional activity to treat AD, such as AChE inhibitory potency, antioxidant activity, anti-inflammatory property, metal chelation property and neuroprotective effect.

Herein, a series of novel naringenin-*O*-alkylamine derivatives are rationally designed by MTDLs. These derivatives are synthesised and evaluated by AChE/BuChE inhibition, antioxidant property, inhibition of A*β* aggregation, metal chelation property, neuroprotective effect and anti-inflammatory effect. The leading compound was further investigated by hepatotoxicity and hepatoprotective activity *in vitro*, and scopolamine-induced mice memory impairment *in vivo*.

## 2. Results and discussion

### 2.1. Chemistry

**Scheme 1.**
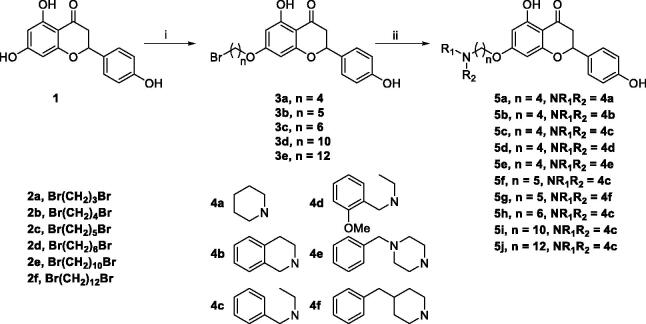
Synthesis of 7-*O*-modified naringenin derivatives **5a–5j**. *Reagents and conditions:* (i) Br(CH_2_)_n_Br (**2b–2f**), K_2_CO_3_, CH_3_CN, 65 °C, 8–12 h; (ii) R_1_R_2_NH (**4a–4f**), K_2_CO_3_, CH_3_CN, 65 °C 6–10 h.

**Scheme 2.**
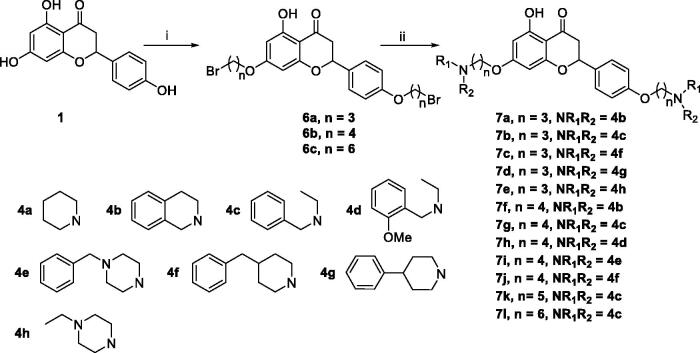
Synthesis of 7,4’-*O*-modified naringenin derivatives **7a–7l**. *Reagents and conditions:* (i) Br(CH_2_)_n_Br (**2a, 2b** and **2d**), K_2_CO_3_, CH_3_CN, 65 °C, 10–15 h; (ii) R_1_R_2_NH (**4a–4h**), K_2_CO_3_, CH_3_CN, 65 °C 8–12 h.

**Scheme 3.**
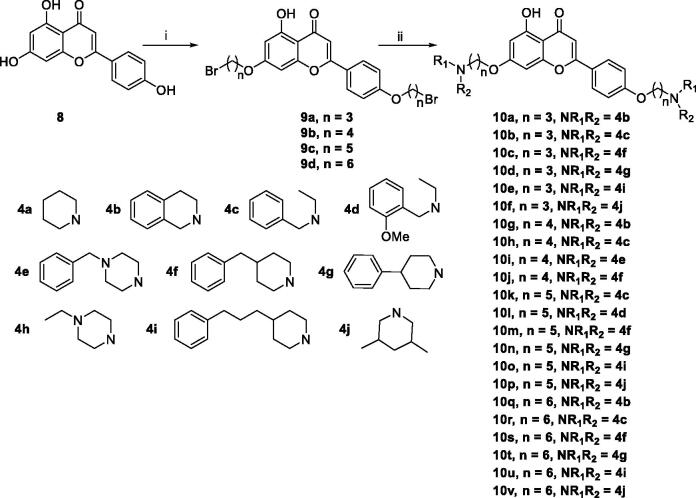
Synthesis of 7,4’-*O*-modified apigenin derivatives **10a–10v**. *Reagents and conditions:* (i) Br(CH_2_)_n_Br (**2a–2d**), K_2_CO_3_, CH_3_CN, 65 °C, 10–15 h; (ii) R_1_R_2_NH (**4a–4j**), K_2_CO_3_, CH_3_CN, 65 °C 8–12 h.

The target compounds **5a–5j**, **7a–7l** and **10a–10v** were synthesised according to Scheme 1–3. Naringenin possessed three hydroxy groups at 4′-, 5-, 7-position, respectively. The 7-OH group showed more acidity than 4′-OH group, and the 5-OH group is the weaker nucleophilic of naringenin due to the formation of intramolecular hydrogen bond with a carbonyl group at 4 position^21^. Given the difference in reactivity of the OH group, it is easy to regioselective make substitutions at 7-OH or/and 4′-OH position. Synthesis of 7-*O*-modified naringenin derivatives was displayed in Scheme 1. The starting material naringenin **1** was treated with 1.2 equivalent dibromides (1,4-dibromobutane **2b** or 1,5-dibromopentane **2c**, 1,6-dibromohexane **2d**, 1,10-dibromodecane **2e**, and 1,12-dibromododecane, respectively) in the presence of 1.3 equivalent of K_2_CO_3_ at 65 °C for 8–12 h to get key intermediates **3a–3c**. Subsequently, compounds **3a–3c** were reacted with 2.0 equivalent of secondary amines **4a–4f** in the presence of 1.3 equivalent of K_2_CO_3_ at 65 °C for 6–10 h to obtain 7-*O*-modified naringenin derivatives **5a–5j** with good yields (Table 1).

**Table 1.** The property and yield of 7-*O*-modified naringenin derivatives.

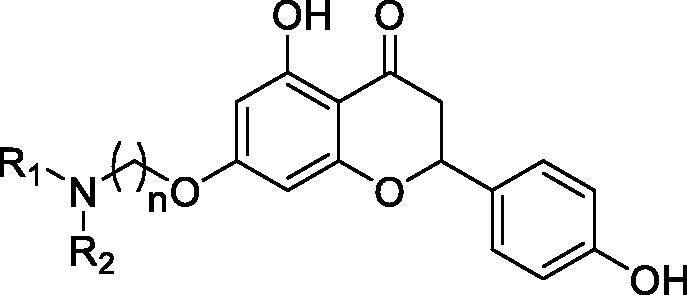				
Compound	*n*	NR_1_R_2_	Property	Yield (%)
**5a**	4	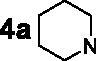	Light yellow oily matter	76.2%
**5b**	4	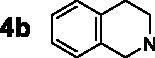	Light yellow oily matter	70.1%
**5c**	4	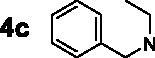	Light yellow oily matter	73.9%
**5d**	4	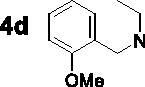	Light yellow oily matter	74.6%
**5e**	4	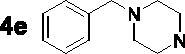	Light yellow oily matter	70.3%
**5f**	5	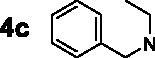	Light yellow oily matter	68.7%
**5g**	5	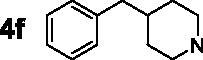	Light yellow oily matter	60.7%
**5h**	6	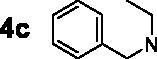	Light yellow oily matter	62.4%
**5i**	10	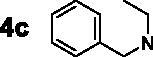	Light yellow oily matter	52.6%
**5j**	12	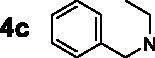	Light yellow oily matter	51.7%

For the synthesis of 7,4′-*O*-modified naringenin derivatives **7a–7k** (Scheme 2), compounds **6a–6c** were the key intermediates, which were prepared by using dibromides **2a–2d** in the presence of base following the described method. Thus, naringenin was alkylated with 3.0 equivalent of K_2_CO_3_ in CH_3_CN at 65 °C for 10–15 h followed by the addition of 5.5 equivalent dibromides (**2a**, **2b** and **2d)** to get intermediates **6a–6c**, respectively. Then, the target compounds 7,4′-*O*-modified naringenin derivatives **7a–7k** were obtained through reacting **6a–6c** with 3.0 equivalent of secondary amines **4a–4h** in the presence of 3.5 equivalent of K_2_CO_3_ at 65 °C for 8–12 h (Table 2).

**Table 2.** The property and yield of 7,4’-*O*-modified naringenin derivatives **7a–7l.**

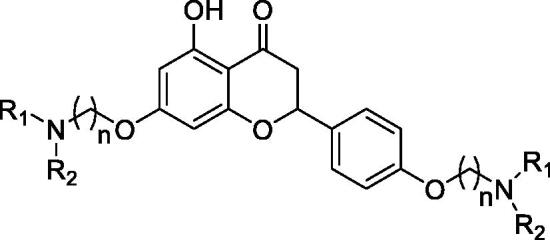				
Compound	*n*	NR_1_R_2_	Property	Yield (%)
**7a**	3	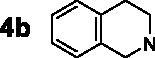	Light yellow oily matter	40.9%
**7b**	3	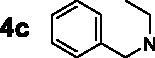	Light yellow oily matter	57.2%
**7c**	3	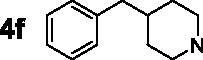	Light yellow oily matter	47.1%
**7d**	3	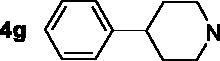	Light yellow oily matter	56.8%
**7e**	3	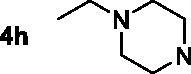	Light yellow oily matter	52.3%
**7f**	4	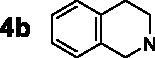	Light yellow oily matter	61.3%
**7g**	4	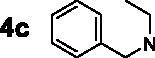	Light yellow oily matter	57.4%
**7h**	4	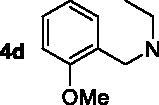	Light yellow oily matter	43.9%
**7i**	4	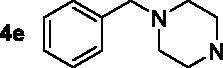	Light yellow oily matter	51.9%
**7j**	4	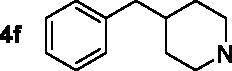	Light yellow oily matter	51.7%
**7k**	5	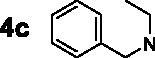	Light yellow oily matter	40.1%
**7l**	6	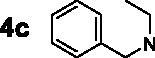	Light yellow oily matter	46.7%

In order to further explore the structure-activity-relationship of naringenin derivatives, we synthesised a series of 7,4′-*O*-modified apigenin derivatives **10a–10v** in Scheme 3. The experiment procedure was similar to the synthesis of 7,4′-*O*-modified naringenin derivatives. The starting material apigenin **8** was treated with 5.5 equivalent dibromides **2a–2d** in the presence of 3.0 equivalent of K_2_CO_3_ in CH_3_CN at 65 °C for 10–15 h to get compounds **9a–9d**. Subsequently, compounds **9a–9d** were treated with secondary amines **4a–4j** (3.0 equivalent) in the presence of K_2_CO_3_ (3.5 equivalent) in CH_3_CN at 65 °C for 8–12 h (Table 3).

**Table 3.** The property and yield of 7,4’-*O*-modified apigenin derivatives **10a–10v**.

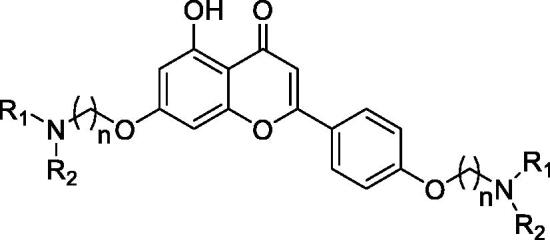				
Compound	*n*	NR_1_R_2_	Property	Yield (%)
**10a**	3	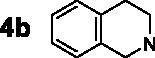	Light yellow oily matter	41.3%
**10b**	3	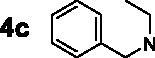	Light yellow oily matter	56.8%
**10c**	3	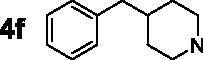	Light yellow oily matter	43.6%
**10d**	3	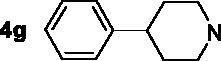	Light yellow oily matter	50.8%
**10e**	3	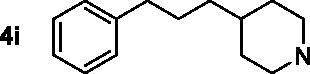	Light yellow oily matter	45.7%
**10f**	3	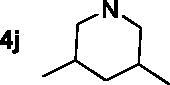	Light yellow oily matter	43.4%
**10g**	4	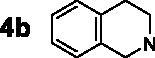	Light yellow oily matter	78.2%
**10h**	4	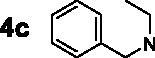	Light yellow oily matter	83.7%
**10i**	4	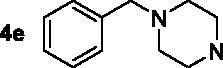	Light yellow oily matter	65.2%
**10j**	4	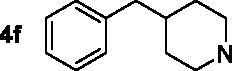	Light yellow oily matter	71.8%
**10k**	5	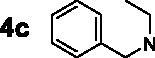	Light yellow oily matter	49.3%
**10l**	5	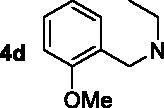	Light yellow oily matter	38.1%
**10m**	5	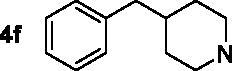	Light yellow oily matter	45.2%
**10n**	5	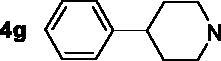	Light yellow oily matter	80.2%
**10o**	5	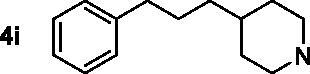	Light yellow oily matter	73.8%
**10p**	5	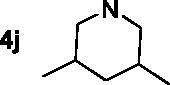	Light yellow oily matter	63.7%
**10q**	6	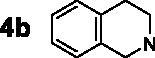	Light yellow oily matter	78.9%
**10r**	6	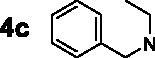	Light yellow oily matter	76.2%
**10s**	6	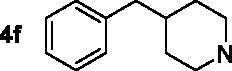	Light yellow oily matter	83.4%
**10t**	6	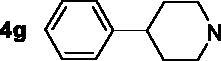	Light yellow oily matter	80.7%
**10u**	6	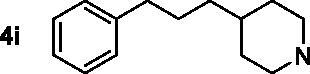	Light yellow oily matter	60.6%
**10v**	6	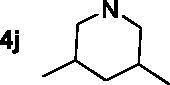	Light yellow oily matter	67.9%

### 2.2. Biological activity

#### 2.2.1. Antioxidant potency

The Oxygen Radicals Absorbance Capacity by Fluorescence (ORAC-FL) assay was employed to test the antioxidant activity of target compounds^22^. The skeleton compounds naringenin and apigenin were also evaluated for comparison purposes, and vitamin E analogue Trolox was used as a standard. As shown in Table 4, the skeleton compounds naringenin and apigenin demonstrated excellent antioxidant activity with ORAC values of 5.2 *eq* and 5.5 *eq*, while introducing alkylamine into the naringenin and apigenin skeleton, respectively, the antioxidant activity significantly decreased. In short, the number of hydroxy groups remarkably influenced the antioxidant potency, the derivatives (**5a**–**5j**) with two hydroxy groups showed better antioxidant activity than the derivatives with one hydroxy group (compounds **7a–7l** and **10a–10v**). While, both the length of methylene and the N terminal secondary amines did not produce an obvious influence on antioxidant activity.

**Table 4.** AChE/BuChE inhibitory activities and oxygen radical absorbance capacity (ORAC, Trolox equivalents) by target compounds **5a–5j**, **7a–7l**, **10a–10v** and the positive compounds naringenin, apigenin, genistein and rivastigmine.

Compound	ORAC*^a^*	IC_50_ ± SD*^b^* (μM)		SI*^e^*	IC_50_ ± SD*^b^* (μM)		SI*^e^*
		*rat*AChE*^c^*	*rat*BChE*^d^*		*hu*AChE*^f^*	*hu*BuChE*^g^*	
**5a**	2.2 ± 0.03	6.7 ± 0.31	20.9 ± 0.68	3.1	NT*^h^*	NT*^h^*	–
**5b**	2.1 ± 0.04	5.8 ± 0.19	17.4 ± 0.52	3.0	NT*^h^*	NT*^h^*	–
**5c**	2.3 ± 0.02	3.9 ± 0.44	22.6 ± 0.23	5.8	NT*^h^*	NT*^h^*	–
**5d**	2.2 ± 0.02	2.0 ± 0.46	18.1 ± 0.56	9.1	NT*^h^*	NT*^h^*	–
**5e**	2.5 ± 0.01	9.2 ± 0.72	13.2 ± 0.43	1.4	NT*^h^*	NT*^h^*	–
**5f**	2.3 ± 0.03	1.7 ± 0.08	16.9 ± 0.27	9.9	0.91 ± 0.02	6.3 ± 0.74	6.9
**5g**	2.1 ± 0.02	2.5 ± 0.33	18.7 ± 0.33	7.5	NT*^h^*	NT*^h^*	–
**5h**	2.3 ± 0.03	3.3 ± 0.09	15.5 ± 0.52	4.7	NT*^h^*	NT*^h^*	–
**5i**	2.1 ± 0.04	4.9 ± 0.16	14.7 ± 0.62	3.0	NT*^h^*	NT*^h^*	–
**5j**	2.2 ± 0.05	6.5 ± 0.09	15.9 ± 0.37	2.9	NT*^h^*	NT*^h^*	–
**7a**	1.1 ± 0.02	10.1 ± 0.41	20.2 ± 0.23	2.0	NT*^h^*	NT*^h^*	–
**7b**	1.2 ± 0.04	7.4 ± 0.29	18.7 ± 0.37	2.5	NT*^h^*	NT*^h^*	–
**7c**	1.2 ± 0.02	6.7 ± 0.16	19.4 ± 0.28	2.9	NT*^h^*	NT*^h^*	–
**7d**	1.0 ± 0.03	9.3 ± 0.37	20.3 ± 0.26	2.2	NT*^h^*	NT*^h^*	–
**7e**	1.5 ± 0.02	8.2 ± 0.47	15.8 ± 0.62	1.9	NT*^h^*	NT*^h^*	–
**7f**	1.1 ± 0.03	5.4 ± 0.16	20.7 ± 0.55	3.8	NT*^h^*	NT*^h^*	–
**7g**	1.2 ± 0.04	1.4 ± 0.08	15.7 ± 0.29	11.2	NT*^h^*	NT*^h^*	–
**7h**	1.0 ± 0.04	0.86 ± 0.05	16.9 ± 0.34	19.7	NT*^h^*	NT*^h^*	–
**7i**	1.4 ± 0.03	6.1 ± 0.21	12.3 ± 0.16	2.0	NT*^h^*	NT*^h^*	–
**7j**	1.1 ± 0.03	1.4 ± 0.08	15.7 ± 0.29	11.2	NT*^h^*	NT*^h^*	–
**7k**	1.2 ± 0.02	0.79 ± 0.06	14.8 ± 0.36	18.7	0.57 ± 0.02	8.2 ± 0.37	14.4
**7l**	1.2 ± 0.01	2.2 ± 0.13	15.1 ± 0.28	6.9	NT*^h^*	NT*^h^*	–
**10a**	1.1 ± 0.01	8.1 ± 0.27	16.8 ± 0.36	2.1	NT*^h^*	NT*^h^*	–
**10b**	1.0 ± 0.04	4.3 ± 0.38	18.6 ± 0.24	4.3	NT*^h^*	NT*^h^*	–
**10c**	1.2 ± 0.03	5.5 ± 0.17	13.6 ± 0.67	2.5	NT*^h^*	NT*^h^*	–
**10d**	1.1 ± 0.02	6.4 ± 0.22	15.1 ± 0.49	2.4	NT*^h^*	NT*^h^*	–
**10e**	1.1 ± 0.02	7.9 ± 0.26	13.8 ± 0.43	2.3	NT*^h^*	NT*^h^*	–
**10f**	1.0 ± 0.04	8.6 ± 0.33	16.7 ± 0.52	1.9	NT*^h^*	NT*^h^*	–
**10g**	1.2 ± 0.03	4.6 ± 0.21	17.4 ± 0.35	3.8	NT*^h^*	NT*^h^*	–
**10h**	1.1 ± 0.03	0.92 ± 0.06	12.9 ± 0.51	14.0	0.16 ± 0.01	3.7 ± 0.06	23.1
**10i**	1.5 ± 0.02	6.9 ± 0.14	10.3 ± 0.19	1.7	NT*^h^*	NT*^h^*	–
**10j**	1.2 ± 0.03	1.8 ± 0.04	15.7 ± 0.24	8.7	NT*^h^*	NT*^h^*	–
**10k**	1.1 ± 0.02	0.81 ± 0.05	13.3 ± 0.27	16.4	0.13 ± 0.01	4.5 ± 0.12	34.6
**10l**	1.3 ± 0.03	0.62 ± 0.05	14.4 ± 0.32	23.2	0.09 ± 0.002	2.2 ± 0.18	24.4
**10m**	1.2 ± 0.02	1.3 ± 0.08	16.6 ± 0.27	12.8	NT*^h^*	NT*^h^*	–
**10n**	1.1 ± 0.04	3.6 ± 0.11	17.9 ± 0.46	4.9	NT*^h^*	NT*^h^*	–
**10o**	1.2 ± 0.03	4.1 ± 0.63	16.1 ± 0.25	3.9	NT*^h^*	NT*^h^*	–
**10p**	1.1 ± 0.02	6.2 ± 0.54	13.8 ± 0.37	2.2	NT*^h^*	NT*^h^*	–
**10q**	1.0 ± 0.02	4.2 ± 0.15	12.2 ± 0.28	2.9	NT*^h^*	NT*^h^*	–
**10r**	1.2 ± 0.04	1.7 ± 0.21	14.1 ± 0.22	8.3	NT*^h^*	NT*^h^*	–
**10s**	1.1 ± 0.03	2.6 ± 0.33	16.9 ± 0.54	6.5	NT*^h^*	NT*^h^*	–
**10t**	1.0 ± 0.02	5.9 ± 0.37	19.3 ± 0.67	3.3	NT*^h^*	NT*^h^*	–
**10u**	1.2 ± 0.03	6.3 ± 0.21	17.2 ± 0.45	2.7	NT*^h^*	NT*^h^*	–
**10v**	1.3 ± 0.03	8.1 ± 0.49	14.8 ± 0.29	1.8	NT*^h^*	NT*^h^*	–
**naringenin**	5.2 ± 0.29	>50	>50	–	NT*^h^*	NT*^h^*	–
**apigenin**	5.6 ± 0.52	>50	>50	–	NT*^h^*	NT*^h^*	–
**donepezil**	NT*^h^*	0.017 ± 0.003	16.2 ± 0.31	953	0.013 ± 0.0004	2.7 ± 0.06	208

*^a^*Results are expressed as μM of Trolox equivalent/μM of tested compound. *^b^*Values are expressed as the mean ± standard deviation of the mean by 3 independent experiments in triplicate. *^c^*From 5% rat cortex homogenate. *^d^*BuChE from rat serum. *^e^*Selectivity Index = IC_50_ (BuChE)/IC_50_ (AChE). *^f^*From human erythrocytes. *^g^*From human serum. *^h^*NT = not tested.

#### 2.2.2. AChE and BChE inhibition of target compounds

The inhibitory potency of the synthesised compounds **5a–5j**, **7a–7l** and **10a–10v** against *rat*AChE (from *rat cortex homogenate*) and *rat*BuChE (from *rat serum*) were evaluated through the slightly modified Ellman’s method^17^. Donepezil served as a positive compound, and naringenin and apigenin were also tested for comparative purposes. The results were listed in Table 4. Firstly, the 7-*O*-modified naringenin derivatives were synthesised and evaluated, the screening data in Table 4 showed that compounds **5a–5j** displayed good *rat*AChE inhibitory activity and weak *rat*BuChE inhibitory potency, indicating that compound **5a–5j** were selective *rat*AChE inhibitors. In addition, both the length of methylene and the N terminal secondary amines produced significantly influence on the *rat*AChE inhibitory activity. When the length of methylene was 4, compound **5a** with piperidine fragment showed good *rat*AChE inhibitory activity with IC_50_ value of 6.7 μM. Replacing piperidine fragment of **5a** with 1,2,3,4-tetrahydroisoquinoline to get compound **5b**, the *rat*AChE inhibitory activity slightly increased to 5.8 μM. Then opening the ring of 1,2,3,4-tetrahydroisoquinoline of compound **5b** to obtain compound **5c** with *N*-ethylbenzylamine fragment, the *rat*AChE inhibitory activity significantly increased to 3.9 μM. And then adding methoxy group at 2-position of *N*-ethylbenzylamine to get compound **5d** with *N*-(2-methoxybenzyl)ethanamine fragment, the *rat*AChE inhibitory activity remarkably increased to 2.0 μM. While, replacing *N*-(2-methoxybenzyl)ethanamine of **5d** with phenylpiperazine fragment to afford compound **5e**, the *rat*AChE inhibitory activity sharply decreased to 9.2 μM. When the length of methylene was 5, compound **5f** with *N*-ethylbenzylamine fragment presented the best *rat*AChE inhibitory activity with IC_50_ value of 1.7 μM, and replacing *N*-ethylbenzylamine of **5f** with benzylpiperidine fragment to get compound **5g**, the *rat*AChE inhibitory activity slightly decreased to 2.5 μM. Correspondingly, when the N terminal secondary amines were fixed and were *N*-ethylbenzylamine fragments, the potencies to inhibit AChE were in the order *n* = 4 (**5c**) < *n* = 5 (**5f**) > *n* = 6 (**5h**) > *n* = 10 (**5i**) > *n* = 12 (**5j**), and the optimal length of methylene was 5.

7,4′-*O*-modified naringenin derivatives **7a–7l** were also synthesised and evaluated to explore the structure-activity-relationship (SAR). The data were listed in Table 4, compounds **7a–7l** showed good ratAChE inhibitory potency and weak ratBuChE inhibitory potency, displaying that all the compounds **7a–7l** were selective *rat*AChE inhibitors. Similarly, both the length of methylene and the N terminal secondary amines created significantly influence on the *rat*AChE inhibitory activity. When the length of methylene was 3, compound **7a** with 1,2,3,4-tetrahydroisoquinoline fragment showed moderate *rat*AChE inhibitory activity with an IC_50_ value of 10.1 μM. Opening the ring of 1,2,3,4-tetrahydroisoquinoline of compound **7a** to obtain compound **7b** with *N*-ethylbenzylamine fragment, the *rat*AChE inhibitory activity significantly increased to 7.4 μM. Replacing *N*-ethylbenzylamine fragment of **7b** with benzylpiperidine to get compound **7c**, the *rat*AChE inhibitory activity slightly increased to 6.7 μM. When replacing benzylpiperidine of compound **7c** with 4-phenylpiperidine to obtain compound **7d**, the *rat*AChE inhibitory activity decreased to 9.3 μM. And replacing benzylpiperidine of compound **7c** with ethylpiperazine to get compound **7e**, the *rat*AChE inhibitory activity decreased to 8.2 μM. When the length of methylene was 4, a similar phenomenon was also observed, such as compound **7f** with 1,2,3,4-tetrahydroisoquinoline (IC_50_ = 5.4 μM) < compound **7g** with *N*-ethylbenzylamine (IC_50_ = 1.4 μM) < compound **7j** with benzylpiperidine (IC_50_ = 1.3 μM). In addition, when replacing *N*-ethylbenzylamine fragment of **7g** with *N*-(2-methoxybenzyl)ethanamine to obtain compound **7h**, the *rat*AChE inhibitory activity increased to 0.86 μM. And replacing benzylpiperidine fragment of **7j** with benzylpiperazine to get compound **7i**, the *rat*AChE inhibitory activity sharply decreased to 6.1 μM. Furthermore, when the N terminal secondary amines were fixed and were *N*-ethylbenzylamine fragment, the potency to inhibit *rat*AChE was in order *n* = 3 (**7b**, 7.4 μM) < *n* = 4 (**7g**, 1.4 μM) < *n* = 5 (**7k**, 0.79 μM) > *n* = 6 (**7l**, 2.2 μM). Thus, the optimal length of methylene was 5. Overall speaking, 7,4′-*O*-modified naringenin derivatives showed better *rat*AChE inhibitory potency than 7-*O*-modified naringenin derivatives, such as, **5b** < **7f**; **5c** < **7g**; **5d** < **7f**; **5e** < **7i**; **5f** < **7k**; **5h** < **7f**.

In order to further explore the SAR, a series of 7,4′-*O*-modified apigenin derivatives **10a–10v** were synthesised by replacing the naringenin skeleton of compounds **7a–7l** with apigenin skeleton. The data were shown in Table 4, all the 7,4′-*O*-modified apigenin derivatives **10a–10v** showed good selective *rat*AChE inhibitory potency, which was consistent with agreed with the 7,4′-*O*-modified naringenin derivatives **7a–7l.** Likewise, both the length of methylene and the N terminal secondary amines significantly influenced the *rat*AChE inhibitory activity. When the length of methylene was 3, the potency of secondary amines to inhibit *rat*AChE was in order *N*-ethylbenzylamine (**10b**, 4.3 μM) > benzylpiperidine (**10c**, 5.5 μM) > 4-phenylpiperidine (**10d**, 6.4 μM) > 1,2,3,4-tetrahydroisoquinoline (**10a**, 7.4 μM) > 4–(3-phenylpropyl)piperidine (**10e**, 8.6 μM) > 3,5-dimethylpiperidine (**10f**, 8.6 μM). A similar phenomenon was also observed when the length of methylene was 4, such as *N*-ethylbenzylamine (**10h**, 0.92 μM) > benzylpiperidine (**10j**, 1.8 μM) > 1,2,3,4-tetrahydroisoquinoline (**10g**, 4.6 μM). Moreover, replacing benzylpiperidine fragment of **10j** with benzylpiperazine to get compound **10i**, the *rat*AChE inhibitory activity sharply decreased to 6.9 μM. When the length of methylene was 5, the similar results were also observed, *N*-ethylbenzylamine (**10k**, 0.81 μM) > benzylpiperidine (**10m**, 1.3 μM) > 4-phenylpiperidine (**10n**, 3.6 μM) > 4–(3-phenylpropyl)piperidine (**10o**, 4.1 μM) > 3,5-dimethylpiperidine (**10p**, 6.2 μM), and when replacing *N*-ethylbenzylamine of **10k** with *N*-(2-methoxybenzyl)ethanamine to obtain compound **10l**, the *rat*AChE inhibitory activity increased to 0.62 μM. When the length of methylene was 6, the potency of secondary amines to inhibit *rat*AChE was in order *N*-ethylbenzylamine (**10r**, 1.7 μM) > benzylpiperidine (**10s**, 2.6 μM) > 1,2,3,4-tetrahydroisoquinoline (**10q**, 4.2 μM) > 4-phenylpiperidine (**10t**, 5.9 μM) > 4–(3-phenylpropyl)piperidine (**10u**, 6.3 μM) > 3,5-dimethylpiperidine (**10v**, 8.1 μM). Overall speaking, the derivatives with *N*-ethylbenzylamine, *N*-(2-methoxybenzyl)ethanamine and benzylpiperidine fragment presented good *rat*AChE inhibitory potency. Furthermore, when the the N terminal secondary amines were fixed, the potency of the length of methylene to inhibit *rat*AChE was in order *n* = 5 > *n* = 4 > *n* = 6 > *n* = 3, for example, when the N terminal secondary amine was *N*-ethylbenzylamine, *n* = 5 (**10k**, 0.81 μM) > *n* = 4 (**10h**, 0.92 μM) > *n* = 6 (**10r**, 1.7 μM) > *n* = 3 (**10b**, 4.3 μM); when the N terminal secondary amine was benzylpiperidine, *n* = 5 (**10m**, 1.3 μM) > *n* = 4 (**10j**, 1.8 μM) > *n* = 6 (**10s**, 2.6 μM) > *n* = 3 (**10c**, 5.5 μM).

Based on the above results, compounds **5f**, **7k**, **10h**, **10k** and **10l** presented good *rat*AChE inhibitory potency from the 7-*O*-modified naringenin derivatives **5a–5j**, 7,4′-*O*-modified naringenin derivatives **7a–7l** and 7,4′-*O*-modified apigenin derivatives **10a–10v**, respectively, which were selected to re-evaluate using human erythrocytes AChE (*hu*AChE) and human serum BuChE (*hu*BChE). The results were listed in Table 4, all the representative compounds presented excellent *hu*AChE inhibitory potency and good *hu*BuChE inhibitory potency. As a whole, 7,4′-*O*-modified apigenin derivatives displayed the best *hu*AChE inhibitory activity, such as **10h** (IC_50_ = 0.16 μM), **10k** (IC_50_ = 0.13 μM) and **10l** (IC_50_ = 0.09 μM). While, in our previous work, we have done much work for the representative 7,4′-*O*-modified apigenin derivative by the *in vitro* and *in vivo* assay. And the 7,4′-*O*-modified apigenin derivatives were synthesised for comparison purposes to explore the SAR of 7,4′-*O*-modified naringenin derivatives. So, compounds **5f** and **7k** were selected to perform further investigation.

#### 2.2.3. *Hu*AChE reversibility of inhibition by 5f and 7k

**Figure 2.**
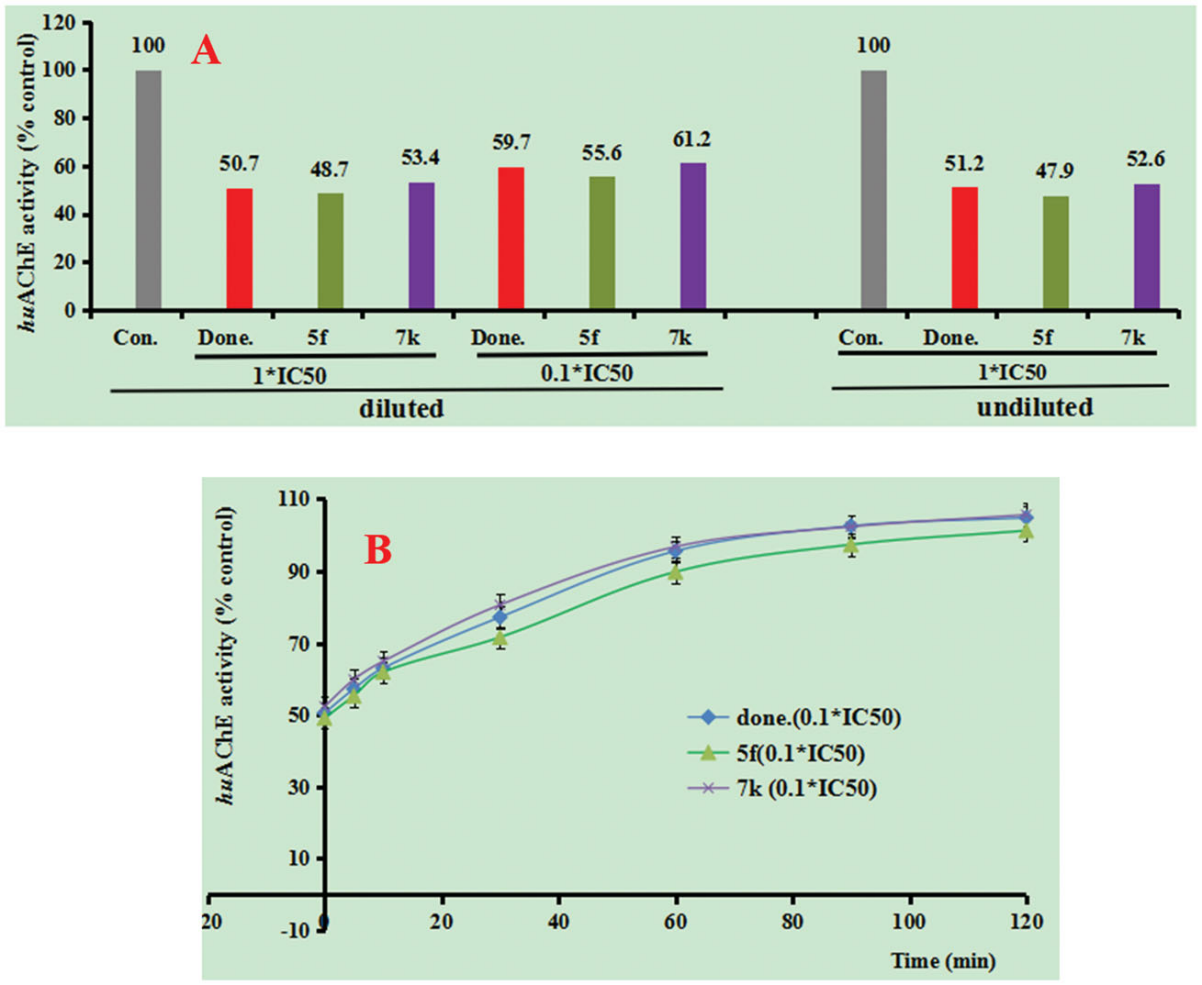
(A) *hu*AChE recovery after preincubation of compounds **5f** and **7k** diluted to 1× or 0.1 × IC_50_, compared to donepezil diluted, and undiluted inhibition. (B) *hu*AChE recovery of donepezil, **5f** and **7k** diluted to 0.1 × IC_50_, were monitored with time at room temperature for 120 min. con. = control, done. = donepezil. Data were expressed as the mean ± SEM by three independent experiments.

To determine whether compounds **5f** and **7k** were reversible *hu*AChE inhibitors, we investigated the recovery of *hu*AChE inhibitors inhibition after dilution (Figure 2A,B)^20^. In Figure 2(A), when the positive compound donepezil dilute to 0.1 × IC_50_, the *hu*AChE activity increased to 9.0%, indicating a reversible *hu*AChE inhibitor, which was consistent with our previous work. When the concentration of compounds **5f** and **7k** was diluted to 0.1 × IC_50_, the *hu*AChE activity increased to 6.9% and 7.8%, respectively, which was in keeping with donepezil. In order to further explore the reversibility of inhibition, the recovery of *hu*AChE inhibitors inhibition after dilution was carried out with time monitoring. As displayed in Figure 2(B), the *hu*AChE activity was restored to 95.6% with 0.1× IC_50_ donepezil at 60 min, and the 0.1 × IC_50_ compounds (**5f** and **7k**) were restored *hu*AChE activity to 89.8% and 96.8%, respectively. The results showed that both **5f** and **7k** were reversible *hu*AChE inhibitors.

#### 2.2.4. Enzemye kinetic study on of huAChE

**Figure 3.**
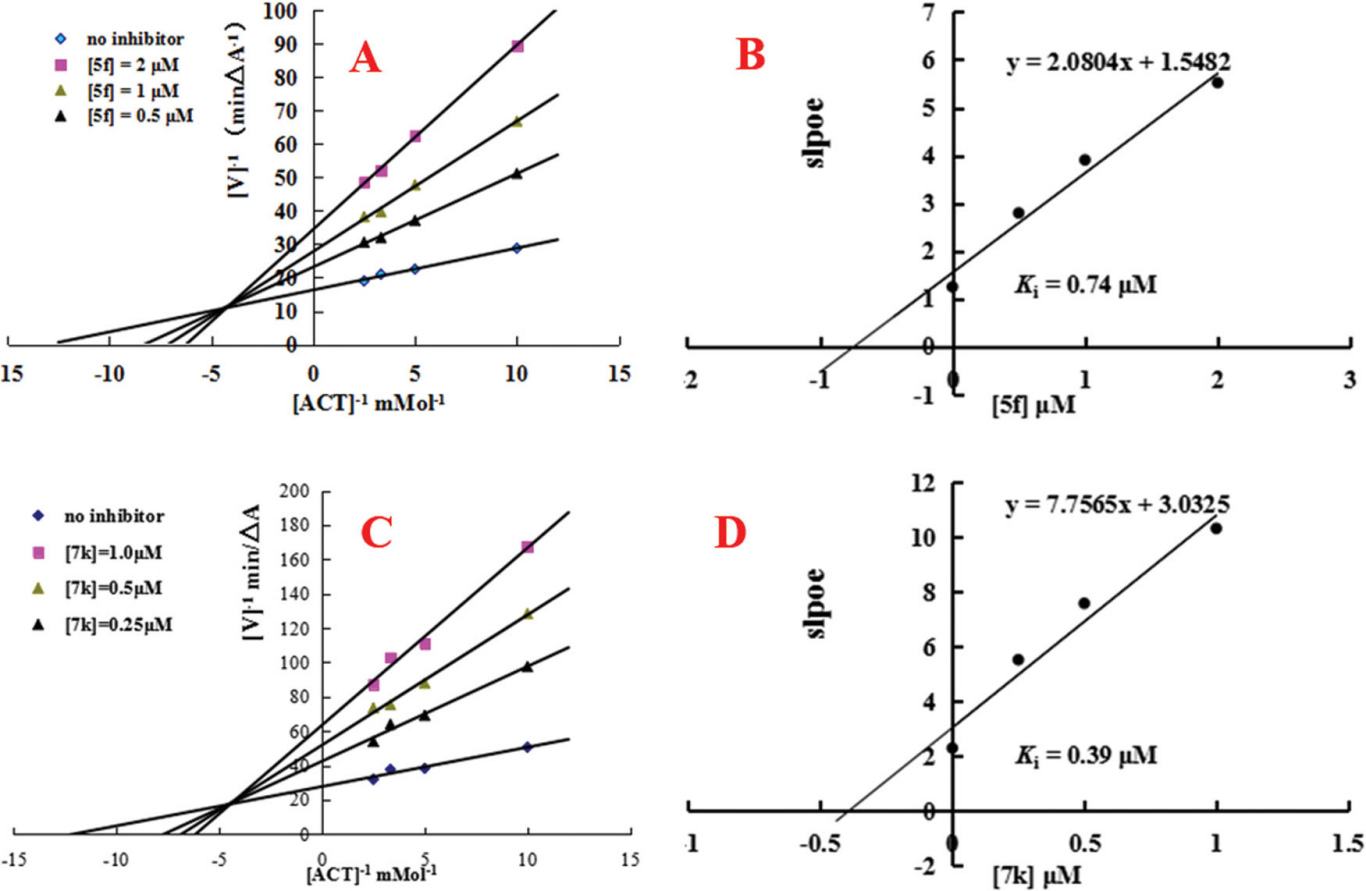
Enzyme kinetic study on the mechanism of *hu*AChE inhibition by compounds **5f** and **7k**. (A) Overlaid Lineweaver-Burk reciprocal plots of AChE initial velocity at increasing acetylthiocholine concentration in the absence and in the presence of **5f** are displayed. (B) Representing the plots of slope versus the concentration of **5f** for determining the inhibition constants *K*_i_. (C) Overlaid Lineweaver-Burk reciprocal plots of AChE initial velocity at increasing acetylthiocholine concentration in the absence and in the presence of **7k** are displayed. (D) Representing the plots of slope versus the concentration of **7k** for determining the inhibition constants *K*_i_.

An enzyme kinetic study was applied to determine the mechanism of AChE inhibition for compounds **5f** and **7k**. The lineweaver-Burk double reciprocal plot was used to define the inhibition by plotting the initial velocities of the substrate at the y-axis and increasing concentrations of the substrate at the x-axis^17^^,^^19^. As shown in Figure 3(A), decreased Vmax and increased Ki values corresponding to increasing concentration of tested compound **5f** exhibited a mixed-type of AChE inhibition. As shown in Figure 3(B), replots of the slope versus concentration of compound **5f** gave an estimate of the inhibition constant and the *K*_i_ value was 0.74 μM. Similarly, Figure 3(C) showed that compound **7k** also demonstrated a mixed-type of AChE inhibition, and the Ki value was 0.39 μM (Figure 3(D)). The results exhibited that compounds **5f** and **7k** were mixed-type of AChE inhibitors and were able to simultaneously bind both catalytic active site (CAS) and peripheral anionic site (PAS) of AChE.

#### 2.2.5. Molecular docking of compounds 5f and 7k with AChE

**Figure 4.**
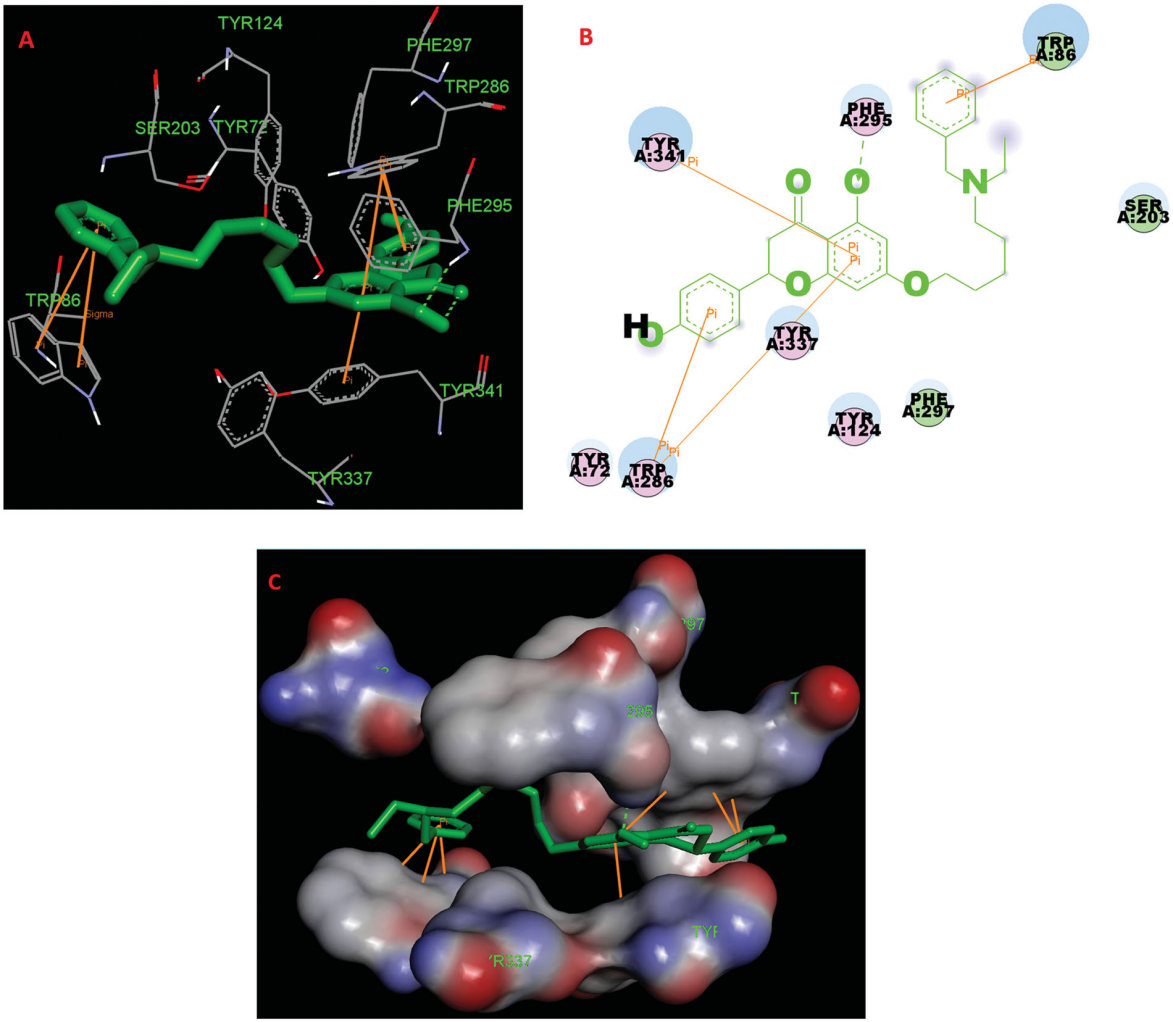
(A) Compound **5f** (green stick) acted on residues in the binding site of *hu*AChE (PDB code: 4ey4). (B) 2 D docking model of **5f** with *hu*AChE. (C) 3 D docking model of **5f** with *hu*AChE.

The possible interacting mechanism of compounds **5f** and **7k** with *hu*AChE (PDB code: 4ey4) was carried out using AUTODOCK 4.2 package^20^. It had been verified that amino acid residues Tyr72, Asp74, Trp86, Tyr124, Trp286, Tyr337, Phe295 and Phe297 were the key active sites residues of *hu*AChE^23^. As displayed in **5f**-*hu*AChE complex (Figure 4), the carbonyl group at 4-position interacted with the hydroxy group at 5-position *via* one intramolecular hydrogen bonding, and the O atom of the hydroxy group interacted with Phe295 *via* one intermolecular hydrogen bonding. The benzene ring of naringenin interacted with Trp286 and Tyr341 *via* one Pi-Pi interaction, respectively. The benzene ring at 2-position interacted with key residue Trp286 via two Pi-Pi interactions. The benzene ring of the alkylamine side chain at 7-position interacted with key residue Trp86 via two Pi-Pi interactions and one Sigma-Pi interaction. Besides, some hydrophobic interactions were presented between compound **5f** and residues (such as Trp86, Tyr124, Tyr337, Phe295, Tyr341 and Trp286). The above interactions offered a possible mechanism for its high AChE inhibitory activity.

**Figure 5.**
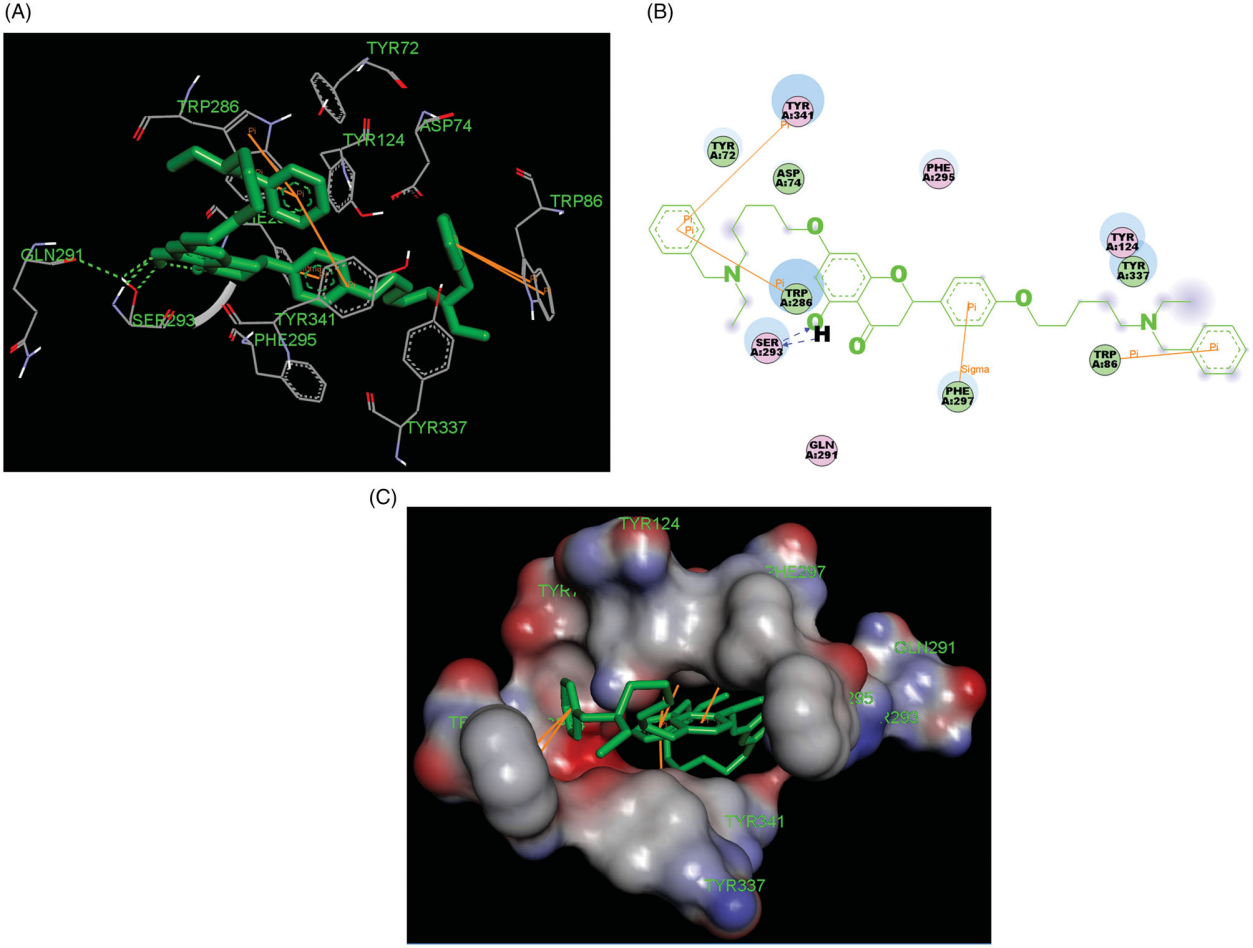
(A) Compound **7k** (green stick) acted on residues in the binding site of *hu*AChE (PDB code: 4ey4). (B) 2 D docking model of **7k** with *hu*AChE. (C) 3 D docking model of **7k** with *hu*AChE.

In **7k**-*hu*AChE complex (Figure 5), compound **7k** simultaneously occupied the entire *hu*AChE enzymatic catalytic site (CAS), the mid-gorge sites and the peripheral anionic site (PAS). compound **7k** interacted with huAChE through multi-interactions. The carbonyl group at 4-position interacted with the hydroxy group at 5-position *via* one intramolecular hydrogen bonding. The H atom of the hydroxy group interacted with Ser293 *via* one intermolecular hydrogen bonding, and the O atom of the hydroxy group interacted with Ser293 *via* two intermolecular hydrogen bonding. The benzene ring at 2-position interacted with key residue Phe297 via one Sigma-Pi interaction. The benzene ring of the alkylamine side chain at 4′-position interacted with key residue Trp286 via two Pi-Pi interactions. The benzene ring of the alkylamine side chain at 7-position interacted with key residue Trp286 via two Pi-Pi interactions and simultaneously interacted with key residue Tyr337 via one Pi-Pi interaction. Furthermore, some hydrophobic interactions could also be observed between the ligand **7k** and Tyr341, Trp286, Ser293, Phe297, Tyr124 and Tyr337. In general, the interactions between compound **7k** and *hu*AChE provided a reasonable explanation for its potent *hu*AChE inhibitory potency.

#### 2.2.6. Molecular dynamics (MD) simulations

**Figure 6.**
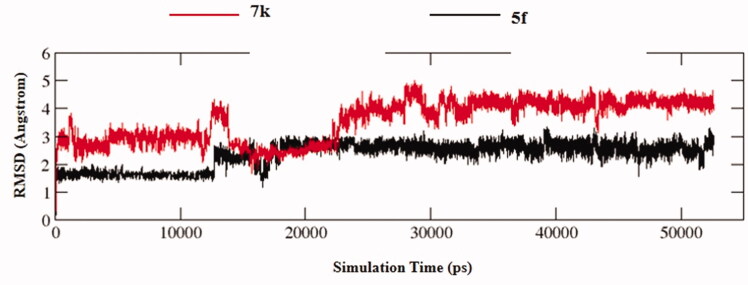
RMSD analysis of compounds **5f** (black stick) and **7k** (red stick).

**Figure 7.**
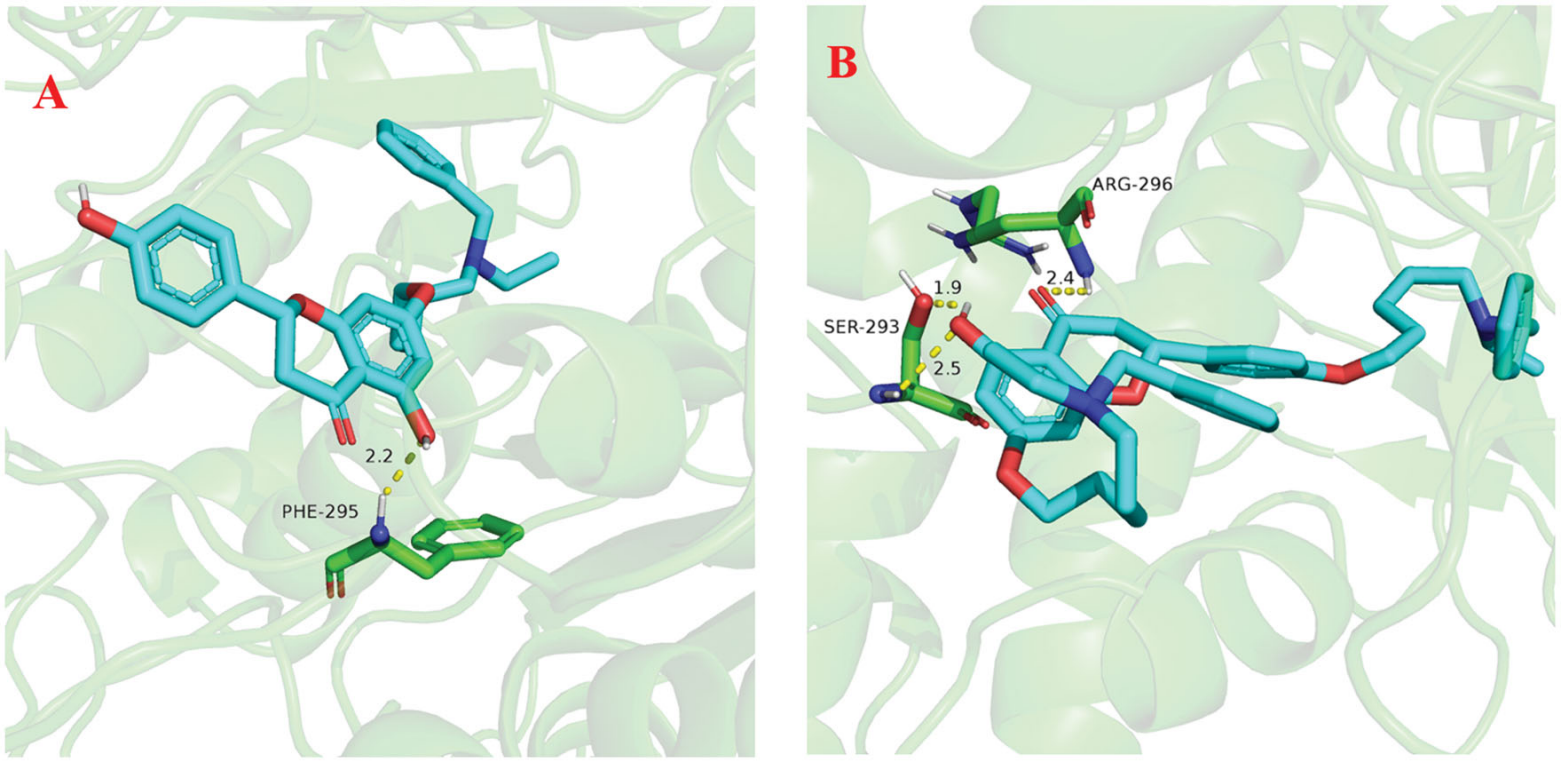
(A) The docking model for **5f** into the protein crystal structure of *hu*AChE (PDB code: 4ey4). (B) The docking model for **7k** into the protein crystal structure of *hu*AChE (PDB code: 4ey4).

The stability of docked binding pose of the compound **5f**-AChE complex and **7k**-AChE were analysed by molecular dynamics simulation analysis using Amber 16^24^. Figure 6 showed the root means square deviations (RMSD) analysis of compounds **5f** and **7k** with the amino acid residues of AChE, respectively. The results demonstrated that the RMSDs of all the replicas for the six simulated systems presented relatively stable fluctuations after 50 ns of the MSMD simulations, displaying that the six simulated systems basically reach equilibrium. The binding free energies of compounds **5f** and **7k** towards *hu*AChE calculated using MM-PBSA were displayed in Table 5, and have values of −51.98 and −64.42 kcal/mol, respectively, which were mainly contributed by Vander Waals forces, electrostatic interactions and non-polar solvation energies^24^. Furthermore, Figure 7 showed the key residues and interaction modes of compounds **5f** and **7k** with *hu*AChE. In Figure 7(A), the hydroxy group of naringenin skeleton formed one intermolecular hydrogen bonding with the key amino acid Phe295 (2.2 Å). Figure 7(B) displayed the interactions modes of **7k** with AChE, and three intermolecular hydrogen bonding were observed. The hydroxy group of naringenin skeleton interacted with key amino acid residue Ser293 *via* two intermolecular hydrogen-bonding interactions with the distance of 1.9 Å and 2.5 Å, respectively. Moreover, the carbonyl of the naringenin skeleton formed one intermolecular hydrogen bonding interaction with the key amino acid Arg296 (2.4 Å).

**Table 5.** The binding free energy and components of **5f** and **7k** (kcal/mol)

Terms	Δ*E*_vdw_	Δ*E*_ele_	Δ*E*_egb_	Δ*E*_esurf_	Δ*G*_gas_	Δ*G*_solv_	Δ*G*_bind_
**5f**	−59.10 (0.30)	−15.10 (0.42)	27.40 (0.38)	−5.18 (0.02)	−74.20 (0.57)	22.22 (0.37)	−51.98 (0.45)
**7k**	−82.24 (0.42)	−16.34 (0.55)	40.95 (0.45)	−6.80 (0.03)	−98.57 (0.79)	34.15 (0.45)	−64.42 (0.85)

#### 2.2.7. Propidium iodide displacement assay

From the above results, compounds **5f** and **7k** interacted with PAS residues of *hu*AChE. Thus, the propidium iodide displacement assay was employed to evaluate the affinity of compounds **5f** and **7k** (10 and 50 μM), and donepezil was also tested as a positive compound^25–27^. As listed in Table 6, compound **5f** showed higher displacement of propidium iodide (10 μM = 25.7%, 50 μM = 36.9%) than donepezil (10 μM = 20.9%, 50 μM = 33.4%). Correspondingly, compound **7k** demonstrated significant displacement of propidium iodide (10 μM = 23.1%, 50 μM = 34.3%). The results were in agreement with the computational study.

**Table 6.** The data of propidium iodide displacement assay towards compounds **5f**, **7k** and doneipezil.

Compound	Propidium iodide displacement from AChE PAS (% inhibition)^*a*^	
	10 μM	50 μM
**5f**	25.7 ± 1.9	36.9 ± 3.5
**7k**	23.1 ± 1.2	34.3 ± 2.1
**Donepezil**	20.9 ± 2.7	33.4 ± 2.6

*^a^*Propidium iodide displacement assay was performed on AChE to test the ability of compounds **5f** and **7k** to displace propidium with reference to the donepezil at 10 and 50 μM. Data are presented as the mean ± SEM of three independent experiments.

#### 2.2.8. Effects on self-induced Aβ_1–42_ aggregation

In order to evaluate the inhibition and disaggregation effects of compounds **5f** and **7k** on self-induced A*β*_1–42_ aggregation, the Thioflavin T (ThT) fluorescence method was applied^17^^,^^28^. Curcumin was also tested as the positive compound. As listed in Table 7, compounds **5f** and **7k** significantly inhibited self-induced A*β*_1–42_ aggregation at 25 μM with 62.1% and 43.8% inhibition rates, respectively, compared with curcumin (45.9%).

**Table 7.** Effects on self-induced/Cu^2+^-induced A*β*_1–42_ aggregation by compounds **5f, 7k** and curcumin.

Compound	Inhibition of A*β*_1–42_ aggregation^*a*^		Inhibition of *huAChE-induced Aβ_1–40_ aggregation* (%)*^d^*
	Self-induced (%)*^b^*	Cu^2+^-induced (%)*^c^*	
**5f**	62.1 ± 0.38	73.5 ± 0.25	51.7 ± 0.75
**7k**	43.8 ± 0.51	68.7 ± 0.46	43.4 ± 0.43
Donepezil	N.T.*^e^*	N.T.*^e^*	28.2 ± 0.29
Curcumin	45.9 ± 0.67	50.6 ± 0.33	N.T.*^e^*

*^a^*The inhibition effects of A*β*_1–42_ aggregation was tested using thioflavin-T fluorescence assay, data are expressed as the mean ± SEM by three independent experiments. *^b^*Inhibition of self-induced A*β*_1–42_ aggregation, both the concentration of tested compounds and A*β*_1–42_ were 25 μM. *^c^*Inhibition of Cu^2+^-induced A*β*_1–42_ aggregation, both the tested compounds and A*β*_1–42_ were 25 μM. *^d^* The inhibition *hu*AChE-induced A*β*_1–40_ aggregation was tested using ThT fluorescence assay, the concentration of tested inhibitor and A*β*_1–40_ was 100 and 230 μM, respectively, whereas the A*β*_1–40_/AChE ratio was equal to 100/1. Data are expressed as the mean ± SEM through three independent experiments. *^e^* N.T. = not tested.

#### 2.2.9. Effects on huAChE-induced Aβ_1–40_ aggregation by compounds 5f and 7k

Accumulation of evidence displayed that PAS of AChE could bind to the A*β*, and accelerated the formation of amyloid fibrils. So, inhibiting the PAS of AChE could significantly affect A*β* aggregation^3^. According to the results from the enzyme kinetic study, docking study and propidium iodide displacement assay compounds **5f** and **7k** could bind the PAS of AChE. Herein, the inhibition potency of compounds **5f** and **7k** on *hu*AChE-induced A*β*_1–40_ was evaluated by employing the ThT assay^29^. As displayed in Table 7, compounds **5f** and **7k** significantly inhibited *hu*AChE-induced A*β*_1–40_ aggregation 51.7% and 43.4%, respectively, which was better than donepezil (28.2%).

#### 2.2.10. Metal-chelating property

**Figure 8.**
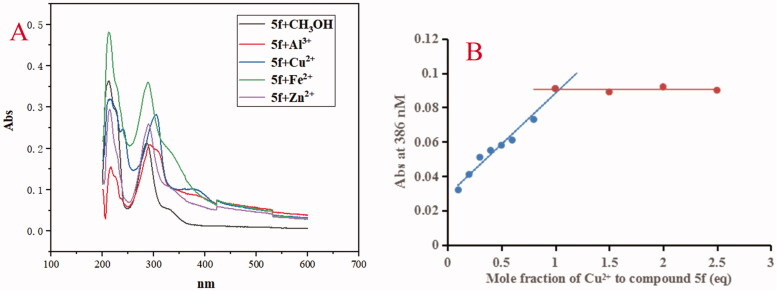
(A) The UV spectrum of compound **5f** (37.5 μM) alone or in the presence of CuCl_2_, FeSO_4_, ZnCl_2_ and AlCl_3_ (37.5 μM) in methanol; (B) Determination of the stoichiometry of complex**-**Cu^2+^ by using molar ratio method at 386 nM.

The chelating property of compounds **5f** and **7k** were determined by UV-visual spectrometry and biometals such as Cu^2+^, Zn^2+^, Al^3+^ and Fe^2+^ were applied^19^^,^^20^. As shown in Figure 8(A), adding CuCl_2_ and AlCl_3_ to a solution of **5f**, the maximum absorption wavelength shifted from 331 nm to 386 nm and 388 nm, respectively, while, when adding FeSO_4_ and ZnCl_2_ to the solution of **5f**, respectively, the maximum absorption at 331 nm did not present obvious shift, exhibiting the formation of **5f-**Cu^2+^ and **5f-**Al^3+^ complex. The stoichiometry of the **5f-**Cu^2+^ complex was determined using the molar ratio method by preparing solutions of compound **5f** with increasing amounts of CuCl_2_ at 386 nm. According to the screening data in Figure 8(B), the absorbance linearly increased at first and then tended to be stable. The two straight lines intersected at a mole fraction of 1.1, revealing a 1:1 stoichiometry for complex **5f**-Cu^2+^.

**Figure 9.**
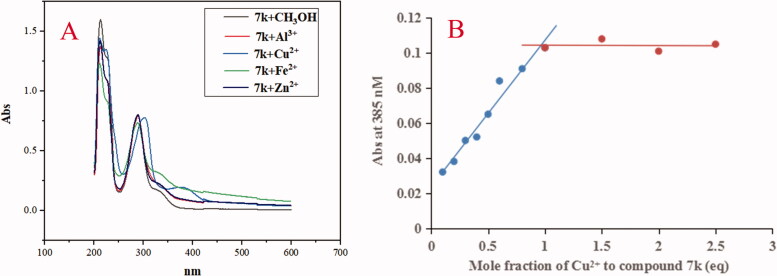
(A) The UV spectrum of compound **7k** (37.5 μM) alone or in the presence of CuCl_2_, FeSO_4_, ZnCl_2_ and AlCl_3_ (37.5 μM) in methanol; (B) Determination of the stoichiometry of complex**-**Cu^2+^ by using molar ratio method at 385 nM. The final concentration of **7k** was 37.5 μM, and the final concentration of Cu^2+^ ranged from 3.75 to 93.75 μM.

Furthermore, when adding CuCl_2_, AlCl_3_, FeSO_4_ and ZnCl_2_ to the solution of **7k**, respectively, the results were shown in Figure 9(A). The maximum absorption wavelength shifted from 330 nm to 385 nm after adding Cu^2+^, while, the maximum absorption wavelength did not show an obvious change after FeSO_4_, AlCl_3_ and ZnCl_2_ to the solution of **7k**, respectively, exhibiting the formation of the **7k-**Cu^2+^ complex. Further, the stoichiometry of the **7k-**Cu^2+^ complex was determined using the molar ratio method at 385 nm. As displayed in Figure 9(B), the absorbance linearly increased at first and then tended to be stable. The two straight lines intersected at a mole fraction of 1.1, revealing a 1:1 stoichiometry for complex **7k**-Cu^2+^. Therefore, the results showed that compounds **5f** and **7k** were selective metal chelation agents.

#### 2.2.11. Effects on Cu^2+^-induced Aβ_1–42_ aggregation by compounds 5f and 7k

**Figure 10.**
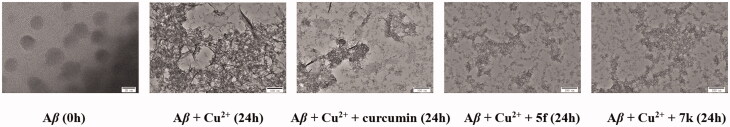
TEM images of A*β* species from inhibition experiments.

Studies indicated that Cu^2+^ could cause A*β* aggregation in solution and accelerated A*β* aggregation. Compounds **5f** and **7k** were selective metal chelators based on the above results, in order to evaluate the effect of compounds **5f** and **7k** on Cu^2+^-induced A*β*_1–42_ aggregation, the ThT fluorescence assay and transmission electron microscopy (TEM) method were employed^19^^,^^20^. Curcumin was also tested as a referenced compound. As listed in Table 7, compounds **5f** and **7k** significantly inhibited Cu^2+^-induced A*β*_1–42_ aggregation with 73.5% and 68.7% inhibition rates, respectively, which were better than that with curcumin (50.6% inhibition rate). Furthermore, the TEM images further supplemented the data of ThT assay. As shown in Figure 10, the fresh A*β*_1–42_ had aggregated into fibrils after adding Cu^2+^, while only small fibre aggregates were observed after treating with curcumin. A similar phenomenon was also observed after adding compounds **5f** and **7k**. Therefore, both the ThT assay and TEM images displayed that the metal chelators **5f** and **7k** could inhibit Cu^2+^-induced A*β*_1–42_ aggregation.

#### 2.2.12. Blood − brain barrier permeation assay *in vitro*

Blood brain barrier (BBB) permeability played an important role in the development of central nervous system disease drugs, especially AD. In order to investigate the BBB permeability of compounds **5f** and **7k**, the parallel artificial membrane permeation assay of the blood − brain barrier (PAMPA-BBB) was employed^30^^,^^31^. First of all, 11 reported drugs were collected to verify this assay, and the following ranges of permeability *P*_e_ (×10^−6 ^cm/s) were established as our previous work reported: *P*_e_ *<* 1.61 demonstrated weak BBB permeation; 1.61 *<* *P*_e_ < 3.44 exhibited uncertain BBB permeation; *P*_e_ > 3.44 displayed high BBB permeation. As listed in Table 8, compounds **5f** and **7k** showed good BBB permeation with 6.9 × 10^−6 ^cm/s and 11.7 × 10^−6 ^cm/s permeability, respectively, which were similar with testosterone and diazepam, exhibiting that compounds **5f** and **7k** could cross BBB through passive diffusion.

**Table 8.** The predictive permeation of compounds **5f** and **7k** by PAMPA-BBB assay.

Compound^*a*^	*P*_e_ (×10^−6^ cm/s)*^b^*	Prediction
Testosterone	17.8 ± 0.93	CNS+
Verapamil	16.3 ± 0.82	CNS+
Clonidine	5.8 ± 0.21	CNS+
Norfloxacin	0.13 ± 0.01	CNS-
**5f**	6.9 ± 0.36	CNS+
**7k**	11.7 ± 0.54	CNS+

*^a^*Compounds **5f** and **7k** were dissolved in DMSO at 5 mg/mL and diluted with PBS/EtOH (70:30). The final concentration of compounds was 100 μg/mL. *^b^*Values are expressed as the mean ± SD of three independent experiments.

#### 2.2.13. Neuroprotective effect

**Figure 11.**
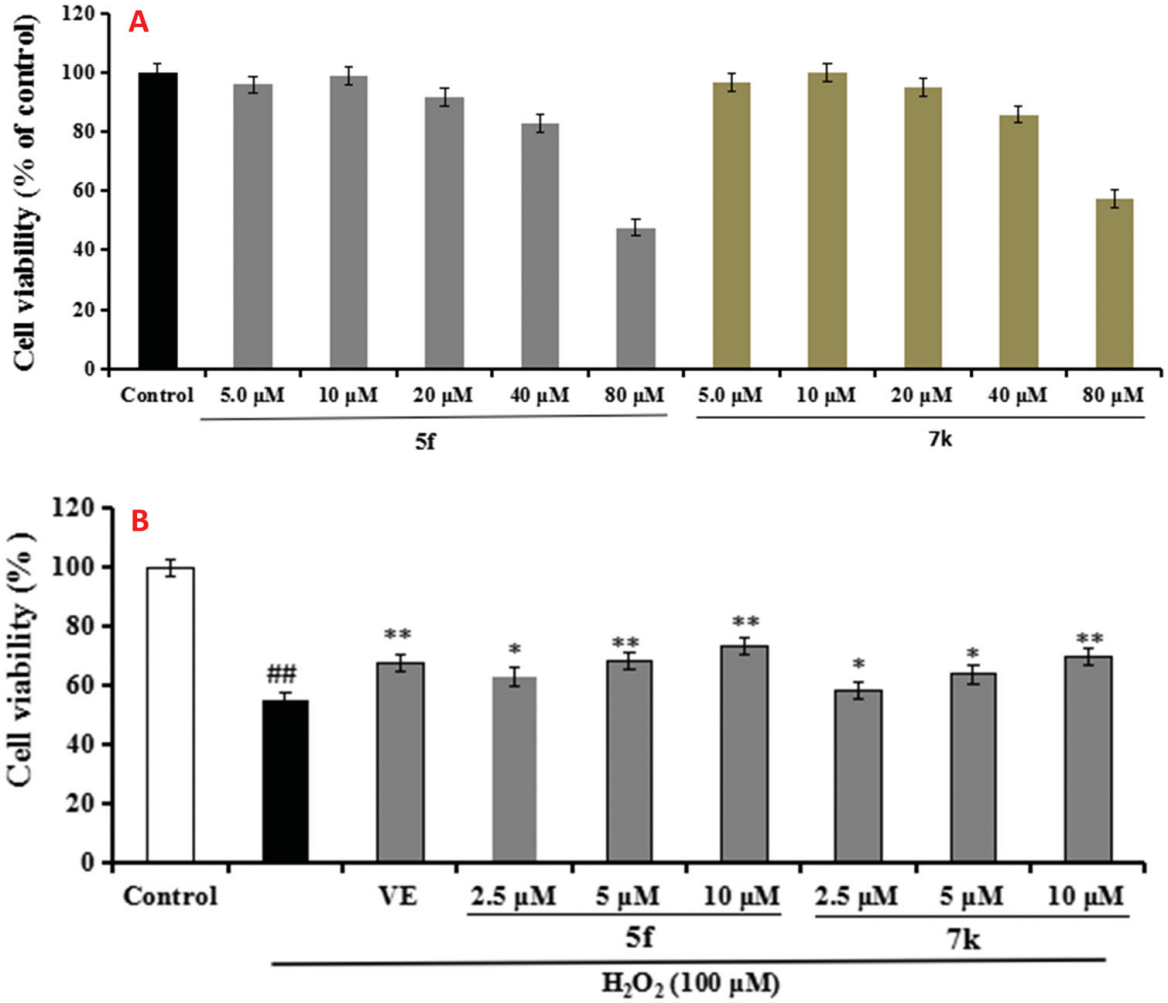
Cell viability was tested by MTT assay. (A) Cytotoxicity of compounds **5f** and **7k** on PC12 cells. (B) Attenuation of H_2_O_2_-induced PC12 cell injury by compounds **5f** and **7k**. values were expressed as mean ± SD by three independent experiments. ##*p* < 0.01 vs control; **p < 0.01, *p < 0.05 vs H_2_O_2_ group.

The neuroprotective effect of compounds **5f** and **7k** on H_2_O_2_-induced PC12 cells injury was evaluated using MTT assay^20^. As presented in Figure 11(A), compounds **5f** and **7k** did not show any cytotoxicity at 40 μM, respectively, indicating a widely safe range. Further, as shown in Figure 11(B), when PC12 cells were exposed to 100 μM H_2_O_2_, the cell viability sharply declined to 54.6% (*p* < 0.01) compared with the untreated group. When adding 100 μM Vitamine E (VE) into the PC12 cells, the cell viability increased to 67.6% (*p* < 0.01). Under the same conditions, when adding compound **5f** (2.5, 5.0 and 10.0 μM) into PC12 cells, the cell viability significantly increased to 62.9% (*p* < 0.05), 68.2% (*p* < 0.01) and 73.4% (*p* < 0.01), respectively, in a dose-dependant manner. Similarly, when treating with compound **7k** (2.5, 5.0 and 10.0 μM), the cell viability increased to 58.3% (*p* < 0.05), 63.7% (*p* < 0.05) and 69.8% (*p* < 0.01), respectively, in a dose-dependant manner. The results showed that compounds **5f** and **7k** showed significant neuroprotective effects on H_2_O_2_-induced PC12 cells injury, and compound **5f** displayed a better neuroprotective effect than **7k**.

#### 2.2.14. Anti-inflammatory property

Compounds **5f** and **7k** were selected to test the anti-inflammatory potency by measuring the production of inflammatory mediators TNF-α and NO in LPS-induced BV-2 cells^32^^,^^33^.

**Figure 12.**
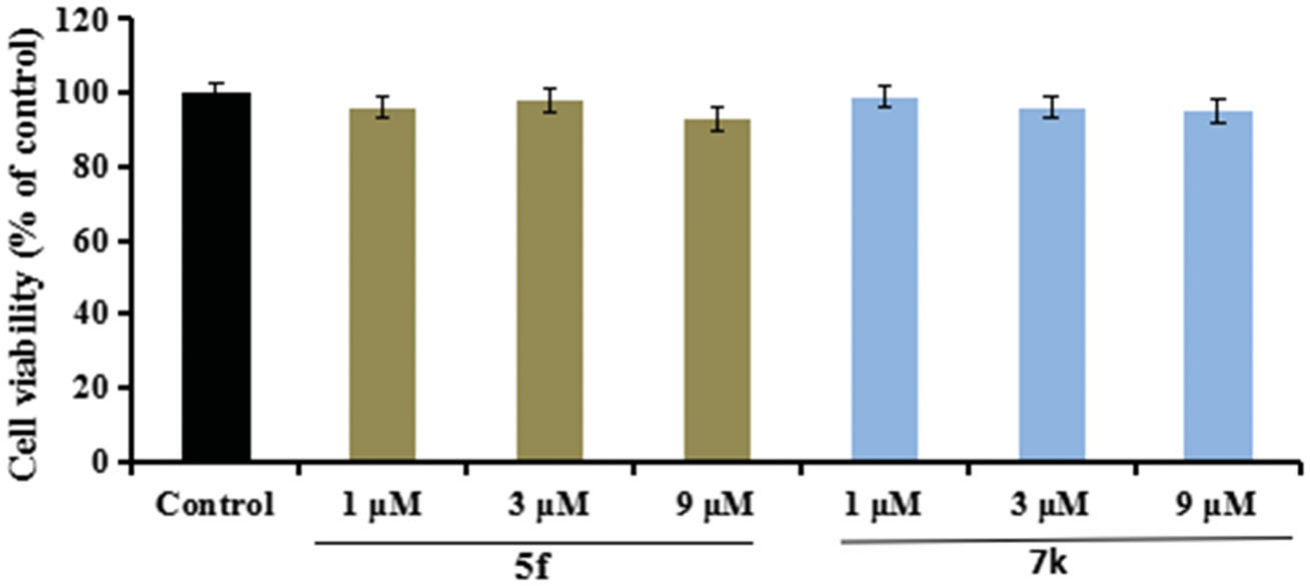
The cell viability of compounds **5f** and **7k** on the BV-2 cells was determined using MTT assay. The data are expressed as the mean ± SD by three independent experiments.

**Figure 13.**
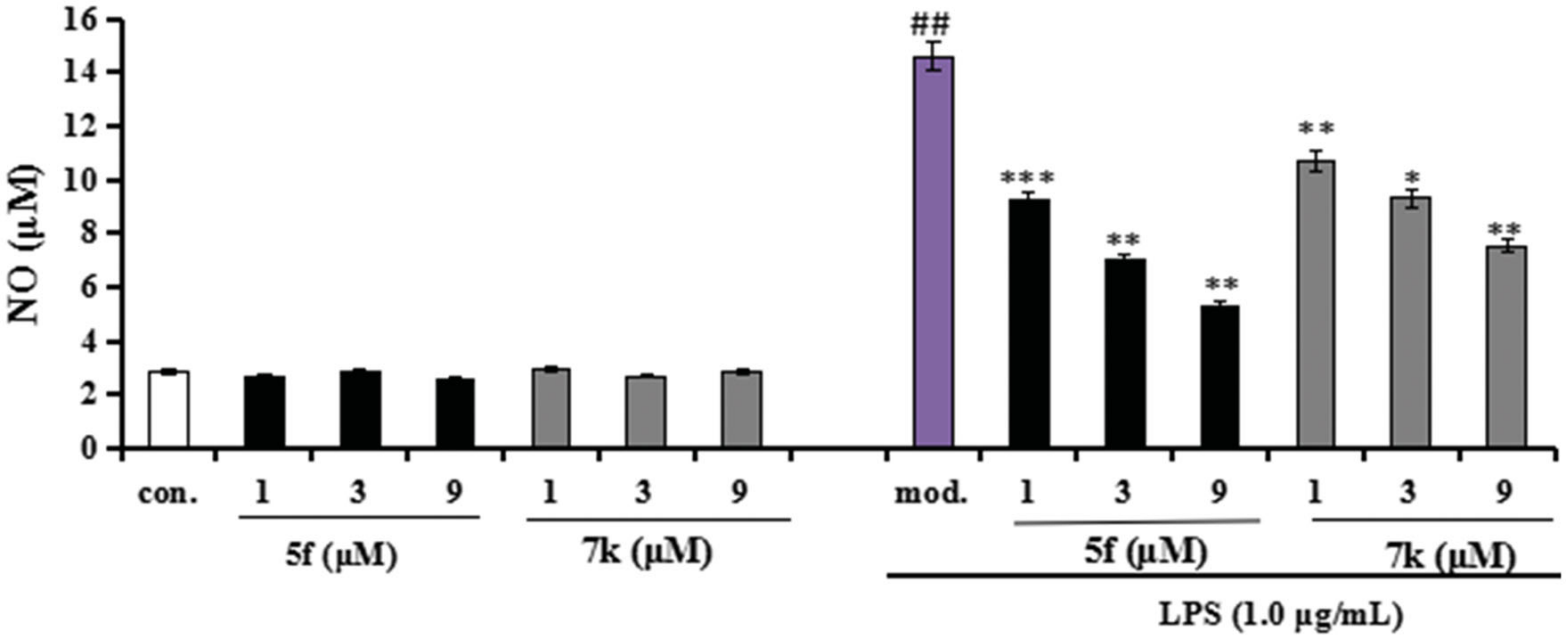
Effects of compounds **5f** and **7k** on NO release in BV-2 cells and LPS-stimulated BV-2 cells. Data were expressed as mean ± SD through three independent experiments. con. = control; mod. = model. ##*p* < 0.01 *vs* control; ***p < 0.01, **p < 0.01, *p < 0.05 *vs* LPS-induced group.

**Figure 14.**
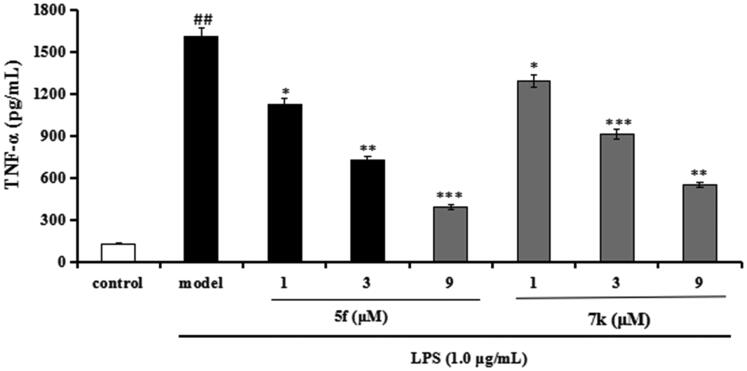
Effects of compounds **5f** and **7k** on TNF-α release in LPS-stimulated BV-2 cells. Data were expressed as mean ± SD through three independent experiments. ##*p* < 0.01 *vs* control; ***p < 0.01, **p < 0.01, *p < 0.05 *vs* LPS-induced group.

1. Cytotoxicity of compounds **5f** and **7k** on BV-2 cells. Firstly, the cytotoxicity of compounds **5f** and **7k** were tested using an MTT assay. As displayed in Figure 12, the cell viability did not show obvious change after adding compounds **5f** or **7k** (1 μM, 3 μM and 9 μM), showing that compounds **5f** and **7k** did not any toxic on BV-2 cells.
2. Evaluation of NO and TNF-α in LPS-stimulated BV-2 cells. In inhibition of LPS-induced, NO production was tested through the Griess reaction method. As shown in Figure 13, the release volume of NO did not produce significant change after adding compounds **5f** or **7k** (1 μM, 3 μM and 9 μM), showing that compounds **5f** and **7k** did not produce an effect on the release of NO in BV-2 cells. When BV-2 cells were exposed to 1 μg/mL LPS, the release volume of NO sharply increased. In addition, when pre-treatment with compound **5f** (1 μM, 3 μM and 9 μM), leading to a remarkable reduction of LPS-induced NO production with 37.6%, 52.1% and 63.8% inhibition rate, respectively, in a dose-dependent manner. Similarly, when pre-treatment with **7k** (1 μM, 3 μM and 9 μM), the percent inhibition rate was 26.7%, 36.2% and 48.5%, respectively, in a dose-dependent manner. In addition, to further investigate the effects of compounds **5f** and **7k** on LPS-induced TNF-α production in BV-2 cells, the enzyme-linked immunosorbent assay (ELISA) was used. As shown in Figure 14, when BV-2 cells were exposed to 1.0 μg/mL LPS, the levels of TNF-α significantly increased to 1615 pg/mL (*p* < 0.01) compared with the untreated group (130 pg/mL). When treatment with compound **5f** (1 μM, 3 μM and 9 μM), the TNF-α production decreased to 1130 pg/mL (*p* < 0.05), 728 pg/mL (*p* < 0.01), 394 pg/mL (*p* < 0.001), respectively, and the inhibitory rate were 33.5%, 57.2% and 76.8%, respectively, in a dose-dependent manner. When treatment with compound **7k** (1 μM, 3 μM and 9 μM), the TNF-α production decreased to 1295 pg/mL (*p* < 0.05), 915 pg/mL (*p* < 0.001), 553 pg/mL (*p* < 0.01), respectively, and the inhibition rates of **7k** were 23.8%, 46.2% and 67.5%, respectively, in a dose-dependent manner. Therefore, compounds **5f** and **7k** reduced the release of NO and suppressed TNF-α production in LPS-induced BV-2 cells, exhibiting that compounds **5f** and **7k** displayed good anti-neuroinflammatory potency *in vitro*.

#### 2.2.15. Theoretical prediction of the ADME properties

The Molinspiration property program was applied to predict the druglike properties of compounds **5f** and **7k**^34^. The items included Log P, MW, TPSA, n-ON, n-OHNH, n-violations n-rotb and volume. The data was listed in Table 9, compound **5f** did not break Lipinski’s rule of five, while compound **7k** broke Lipinski’s rule of five. Therefore, considering the biological activity *in vitro* and prediction of druglike property, compound **5f** was a promising candidate and deserving further investigation.

**Table 9.** Theoretical prediction of the ADME properties of compounds **5f** and **7k.**

Compound^*a*^	Log *P*	MW	TPSA (Å^2^)	n-ON	n-OHNH	n-violations	n-rotb	Volume (Å^3^)
**5f**	5.50	475.58	79.23	6	2	1	11	449.59
**7k**	8.64	678.91	71.48	7	1	2	21	668.93

*^a^*MW, Molecular weight; TPSA, topological polar surface area; n-ON, number of hydrogen acceptors; n-OHNH, number of hydrogen bond donors.

#### 2.2.16. Hepatotoxicity and hepatoprotective activity by compound 5f

**Figure 15.**
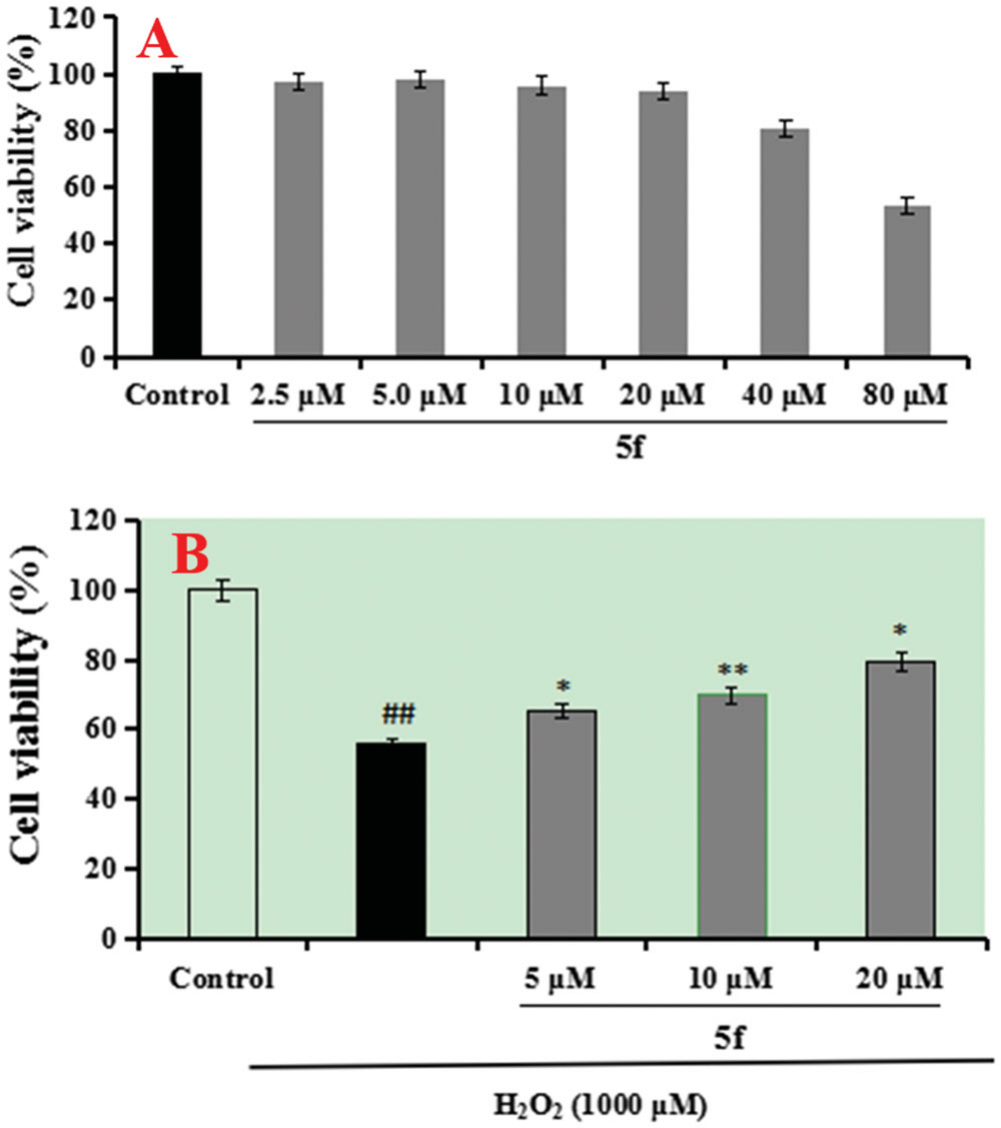
(A) The cell viability of LO2 cell by compound **5f**. (B) The protective effect of compound **5f** on H_2_O_2_-induced hepatic injury, ##*p* < 0.01 *vs* control; **p < 0.01, *p < 0.05 *vs* model group.

The anti-AD drug tacrine has been forced to withdraw due to severe hepatotoxicity. Thus, the hepatotoxicity of compounds **5f** on normal human hepatocytes cell line (LO2) was evaluated using MTT assay^22^. As displayed in Figure 15(A), LO2 cells were exposed to compound **5f** (2.5 μM, 5.0 μM, 10.0 μM, 20.0 μM, 40.0 μM and 80.0 μM), the cell viability did not show an obvious change until the concentration increased to 40.0 μM. Furthermore, the hepatoprotective activity of compound **5f** on H_2_O_2_-induced live injury was tested using an MTT assay. As presented in Figure 15(B), LO2 cells were exposed to 1000 μM H_2_O_2_ for 48 h, cell viability sharply decreased to 55.9% (*p* < 0.01) compared with the untreated group, when treating with compound **5f** (5.0 μM, 10.0 μM and 20.0 μM), the cell viability significantly increased to 65.2% (*p* < 0.05), 69.7% (*p* < 0.01) and 79.4% (*p* < 0.05), respectively, in a dose-dependent manner, exhibiting that compound **5f** displayed potent hepatoprotective activity through its antioxidant potency.

#### 2.2.17. *In vivo* assay

Scopolamine is a muscarinic receptor antagonist that inhibits central cholinergic neuronal activity. Scopolamine-induced *mice memory impairment* has been widely used to evaluate potential therapeutic agents for the treatment of AD. Herein, compound **5f** was selected to perform the experiments *in vivo*^20^^,^^22^.

**Figure 16.**
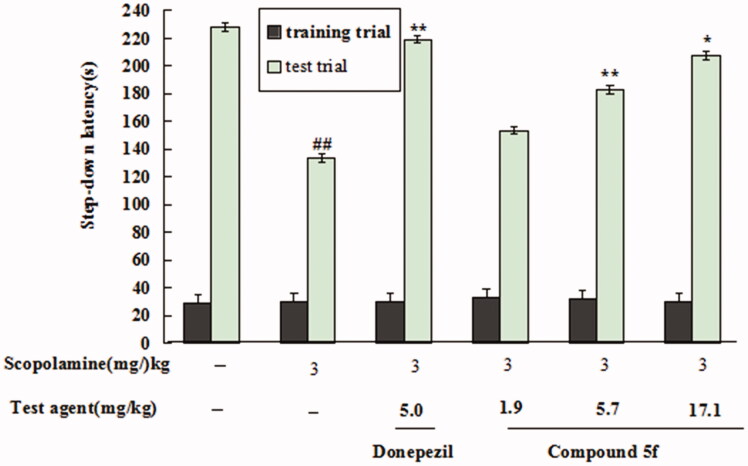
Effect of compound **5f** (1.9, 5.7 and 17.1 mg/kg) or donepezil (5.0 mg/kg) on scopolamine-induced memory impairment through the step-down passive avoidance assay. Values are expressed as the mean ± SEM (*n* = 6). ^##^*p* < 0.01 *vs* untreated group. *p < 0.05 and **p < 0.01 *vs* scopolamine-treated model group.

1. *Acute Toxicity.* The safety profile of compound **5f** was evaluated in SPF Kunming mice (half male and half female) at a body weight of 18**–**20g at doses of 1000, 500, 250 and 100 mg/kg (*n* = 6 per group) by intragastric administration of compound **5f**. After administration, mice were closely observed within 30 min, observation was performed every 15 min from 30 min to 2h, observation was performed every 30 min from 2h to 4h, observation was performed every 1h from 4h to 8h, after that, observation was performed every 1 day, continuous observation for 7 days. The mice were killed after 7 days, and the heart, liver, spleen, lung and kidney were observed. The results showed that the mice at the dose of 1000g/kg and 500 mg/kg presented decreased spontaneous activity and movement, drowsiness, dyspnoea and reduced breathing rate. After 24h, the mice returned to normal. After 7 days, the mice were killed and no abnormalities were observed on the heart, liver, spleen, lung and kidney.
2. *Effect of* ***5f*** *on Scopolamine-induced mice memory impairment.* The step-down passive avoidance task was employed to investigate the effects of **5f** on scopolamine-induced memory impairment. As shown in Figure 16, when mice were treated with 3 mg/kg scopolamine, the step-down latency sharply declined to133.7 sec (model group, *p* < 0.01) compared with saline solution-treated mice (228.3 sec, untreated group). When treating with 5 mg/kg donepezil, the step-down latency significantly improved to (219.4 sec, *p* < 0.01) compared with the model group, indicating that donepezil clearly reversed scopolamine-induced mice cognitive deficit. Moreover, when treating with compound **5f** at concentration of 1.9 mg/kg, 5.7 mg/kg and 17.1 mg/kg, the step-down latency gradually increased to 153.7 sec, 183.4 sec (*p* < 0.01) and 207.8 sec (*p* < 0.05), respectively, in a dose-dependent manner. The results showed that compound **5f** significantly improved scopolamine-induced memory impairment, and the high dose (17.1 mg/kg) presented a similar effect compared with donepezil.

## 3. Conclusion

In summary, a series of naringenin-*O*-alkylamine derivatives were rationally designed as multifunctional agents for treating AD by MTDLs strategy. The target compounds were synthesised and evaluated by antioxidant activity, AChE/BuChE inhibition, inhibition of A*β* aggregation, metal chelation, neuroprotective effect and anti-inflammatory property. The *in vitro* biological activity results revealed that compounds **5f** and **7k** showed good antioxidant activity with ORAC values of 2.3*eq* and 0.57*eq*, respectively. Compounds **5f** and **7k** displayed good *hu*AChE inhibitory potency with IC_50_ values of 0.91 μM and 0.57 μM, respectively, which were supported by molecular docking. Both the kinetic study and propidium iodide displacement assay showed that compounds **5f** and **7k** could simultaneously bind CAS and PAS of *hu*AChE. Moreover, compounds **5f** and **7k** could inhibit self-induced A*β*_1–42_ aggregation with 62.1% and 43.8% inhibition rate, respectively, and significantly inhibited *hu*AChE-A*β*_1–40_ aggregation with 51.7% and 43.4% inhibition rate, respectively. In addition, compounds **5f** and **7k** were selective metal chelation agents and remarkably inhibited Cu^2+^-induced A*β*_1–42_ aggregation with 73.5% and 68.7% inhibition rates, respectively. Furthermore, compounds **5f** and **7k** presented good BBB permeability *in vitro*, good neuroprotective effects and anti-inflammatory properties. Further investigation showed that compound **5f** did not show obvious hepatotoxicity and displayed a good hepatoprotective effect by its antioxidant activity. The *in vivo* study displayed that compound **5f** could improve the dyskinesia recovery rate and response efficiency of the AlCl_3_-induced zebrafish AD model, and further significantly improved scopolamine-induced mice memory impairment. Therefore, compound **5f** was a potential multifunctional candidate for the treatment of AD, deserving further investigation.

## 4. Experimental section

### 4.1. Chemistry

Unless otherwise noted, all the chemicals and solvents were bought from Sigma-Aldrich and Shanghai Titan Scientific Co., Ltd., and were used without purification. The ^1^H NMR (400 MHz) and ^13 ^C NMR (100 MHz) spectra were recorded on a Bruker Varian INOVA spectrometer in deuterated solvents (CDCl_3_) with tetramethylsilane (TMS) as an internal reference. The spectra were measured in chemical shift (δ, ppm) and coupling constant (*J*, Hz). The high-resolution mass spectra were obtained by Waters Xevo G2-XS-Qtof mass spectrometer. The purity of the final synthesised products was evaluated by HPLC analyses which were conducted with a Waters X-Bridge C18 column (4.6 mm × 150 mm, 5 μm) at a flow ratio of 0.8 ml/min. Mobile phase: A: 0.12%TFA in H_2_O, B: 0.1% TFA in CH_3_CN.

#### 4.1.1. Synthesis of intermediates 3a–3e

To a solution of naringenin (10 mmol) in CH_3_CN (30 ml), powdered K_2_CO_3_ (13.0 mmol) was added. After the mixture was stirred at room temperature for 30 min, 12.0 mmol dibromides (1,4-dibromobutane **2b** or 1,5-dibromopentane **2c**, 1,6-dibromohexane **2d**, 1,10-dibromodecane **2e**, and 1,12-dibromododecane **2f**, respectively) were added into the mixture, and then the reaction mixture was stirred at 65 °C for 8–12 h. The reaction was monitored through TLC, the solvent was evaporated under reduced pressure after reaction completion. The crude residue was dissolved in 50 ml ethyl acetate, washed with water (2 × 50 ml), saturated NaCl (80 ml) and passed through the anhydrous Na_2_SO_4_ to remove the residual water. The solvent was evaporated to obtain a crude mixture, which was purified by silica gel chromatography and petroleum ether/acetone = 50:1 as mobile phase to afford intermediates **3a–3e**.

7–(4-bromobutoxy)-5-hydroxy-2–(4-hydroxyphenyl)chroman-4-one (**3a**). The starting material **1** was treated with 1,4-dibromobutane **2b** based on the above procedure to get compound **3a**, colourless oily matter, 53.2% yield. ^1^H NMR (400 MHz, CDCl_3_) *δ* 12.02 (s, 1H, OH), 7.34 (d, *J* = 6.0 Hz, 2H, 2 × Ar-H), 6.89 (d, *J* = 8.0 Hz, 2H, 2 × Ar-H), 6.05 (d, *J* = 2.0 Hz, 1H, Ar-H), 6.02 (d, *J* = 2.4 Hz, 1H, Ar-H), 5.35 (dd, *J*_1_ = 10.4 Hz, *J*_2_ = 2.8 Hz, 1H, CH), 4.00 (t, *J* = 6.4 Hz, 2H, OCH_2_), 3.47 (t, *J* = 6.4 Hz, 2H, BrCH_2_), 3.09 (dd, *J*_1_ = 12.8 Hz, *J*_2_ = 12.8 Hz, 1H, 1/2 CH_2_), 2.79 (dd, *J*_1_ = 10.4 Hz, *J*_2_ = 2.8 Hz, 1H, 1/2 CH_2_), 2.07–2.00 (m, 2H, CH_2_), 1.97–1.91 (m, 2H, CH_2_).

7-((5-bromopentyl)oxy)-5-hydroxy-2–(4-hydroxyphenyl)chroman-4-one (**3b**). The starting material **1** was treated with 1,5-dibromopentane **2c** based on the above procedure to get compound **3b**, colourless oily matter, 46.2% yield. ^1^H NMR (400 MHz, CDCl_3_) *δ* 12.02 (s, 1H, OH), 7.33 (d, *J* = 8.4 Hz, 2H, 2 × Ar-H), 6.89 (d, *J* = 8.8 Hz, 2H, 2 × Ar-H), 6.04 (dd, *J*_1_ = 8.4 Hz, *J*_2_ = 2.4 Hz, 2H, 2 × Ar-H), 5.35 (dd, *J*_1_ = 10.0 Hz, *J*_2_ = 2.8 Hz, 1H, CH), 3.98 (t, *J* = 6.4 Hz, 2H, OCH_2_), 3.43 (t, *J* = 6.8 Hz, 2H, BrCH_2_), 3.09 (dd, *J*_1_ = 12.8 Hz, *J*_2_ = 4.8 Hz, 1H, 1/2 CH_2_), 2.78 (dd, *J*_1_ = 14.0 Hz, *J*_2_ = 3.2 Hz, 1H, 1/2 CH_2_), 1.96–1.88 (m, 2H, CH_2_), 1.84–1.76 (m, 2H, CH_2_), 1.65–1.55 (m, 2H, CH_2_).

7-((6-bromohexyl)oxy)-5-hydroxy-2–(4-hydroxyphenyl)chroman-4-one (**3c**). The starting material **1** was treated with 1,6-dibromohexane **2d** based on the above procedure to get compound **3c**, colourless oily matter, 43.5% yield. ^1^H NMR (400 MHz, CDCl_3_) *δ* 12.01 (s, 1H, OH), 7.32 (d, *J* = 8.0 Hz, 2H, 2 × Ar-H), 6.88 (d, *J* = 8.4 Hz, 2H, 2 × Ar-H), 6.04 (d, *J* = 10.0 Hz, 2H, 2 × Ar-H), 5.35 (dd, *J*_1_ = 10.8 Hz, *J*_2_ = 2.0 Hz, 1H, CH), 3.97 (t, *J* = 6.4 Hz, 2H, OCH_2_), 3.42 (t, *J* = 6.8 Hz, 2H, OCH_2_), 3.08 (dd, *J*_1_ = 13.2 Hz, *J*_2_ = 4.0 Hz, 1H, 1/2 COCH_2_), 2.78 (dd, *J*_1_ = 14.8 Hz, *J*_2_ = 2.4 Hz, 1H, 1/2 COCH_2_), 1.92–1.85 (m, 2H, CH_2_), 1.82–1.75 (m, 2H, CH_2_), 1.49–1.47 (m, 4H, 2 × CH_2_).

7-((10-bromodecyl)oxy)-5-hydroxy-2–(4-hydroxyphenyl)chroman-4-one (**3d**). The starting material **1** was treated with 1,10-dibromodecane **2e** based on the above procedure to get compound **3d**, colourless oily matter, 41.1% yield. ^1^H NMR (400 MHz, CDCl_3_) *δ* 12.02 (s, 1H, OH), 7.34 (d, *J* = 8.0 Hz, 2H, 2 × Ar-H), 6.89 (d, *J* = 8.0 Hz, 2H, 2 × Ar-H), 6.04 (d, *J* = 9.2 Hz, 2H, 2 × Ar-H), 5.35 (dd, *J*_1_ = 10.4 Hz, *J*_2_ = 2.4 Hz, 1H, CH), 3.96 (t, *J* = 6.0 Hz, 2H, OCH_2_), 3.41 (t, *J* = 6.4 Hz, 2H, BrCH_2_), 3.09 (d, *J*_1_ = 13.2 Hz, *J*_2_ = 4.0 Hz, 1H, 1H, 1/2 CH_2_), 2.78 (dd, *J*_1_ = 14.4 Hz, *J*_2_ = 2.4 Hz, 1H, 1/2 CH_2_), 1.89–1.81 (m, 2H, CH_2_), 1.79–1.72 (m, 2H, CH_2_), 1.43–1.37 (m, 4H, 2 × CH_2_), 1.31–1.27 (m, 8H, 4 × CH_2_).

7-((12-bromododecyl)oxy)-5-hydroxy-2–(4-hydroxyphenyl)chroman-4-one (**3e**). The starting material **1** was treated with 1,12-dibromododecane **2f** based on the above procedure to get compound **3e**, colourless oily matter, 33.7% yield. ^1^H NMR (400 MHz, CDCl_3_) *δ* 12.01 (s, 1H, OH), 7.32 (d, *J* = 8.0 Hz, 2H, 2 × Ar-H), 6.88 (d, *J* = 8.4 Hz, 2H, 2 × Ar-H), 6.04 (d, *J* = 9.6 Hz, 2H, 2 × Ar-H), 5.34 (dd, *J*_1_ = 10.8 Hz, *J*_2_ = 2.0 Hz, 1H, CH), 3.95 (t, *J* = 6.4 Hz, 2H, OCH_2_), 3.40 (t, *J* = 6.8 Hz, 2H, BrCH_2_), 3.08 (d, *J*_1_ = 5.2 Hz, *J*_2_ = 4.0 Hz, 1H, 1H, 1/2 CH_2_), 2.78 (dd, *J*_1_ = 14.8 Hz, *J*_2_ = 2.4 Hz, 1H, 1/2 CH_2_), 1.88–1.80 (m, 2H, CH_2_), 1.77–1.72 (m, 2H, CH_2_), 1.43–1.38 (m, 4H, 2 × CH_2_), 1.29–1.27 (m, 12H, 6 × CH_2_).

#### 4.1.2. Synthesis of target 7-*O*-modified naringenin derivatives 5a–5j

To a mixture of the corresponding secondary amines **4a–4f** (1.3 mmol), anhydrous K_2_CO_3_ (1.5 mmol) in CH_3_CN (6 ml) were added the appropriate intermediates **3a–3e** (1.0 mmol). The reaction mixture was heated to 65 °C and stirred for 6–10 h under an argon atmosphere. After complete reaction, the solvent was evaporated under reduced pressure to afford the crude product, which was dissolved in ethyl acetate, washed with water (2 × 30 ml). The combined organic phases were washed with saturated aqueous NaCl (50 ml) and dried over sodium sulphate to remove the residual water. The solvent was evaporated to obtain a crude mixture under reduced pressure, which was purified on a silica gel chromatography using mixtures of CH_2_Cl_2_/acetone = 50:1 as mobile phase to afford the target 7-*O*-modified naringenin derivatives **5a–5j**.

5-hydroxy-2–(4-hydroxyphenyl)-7–(4-(piperidin-1-yl)butoxy)chroman-4-one (**5a**). Compound **3a** was treated with piperidine **4a** based on the above procedure to obtain target compound **5a**, light yellow oily matter, 76.2% yield. ^1^H NMR (400 MHz, CDCl_3_) *δ* 12.03 (s, 1H, OH), 7.34–7.24 (m, 2H, 2 × Ar-H), 6.80 (d, *J* = 8.0 Hz, 2H, 2 × Ar-H), 6.00 (d, *J* = 2.0 Hz, 1H, Ar-H), 5.94 (d, *J* = 2.0 Hz, 1H, Ar-H), 5.27 (dd, *J*_1_ = 10.4 Hz, *J*_2_ = 2.8 Hz, 1H, OCH), 3.91 (t, *J* = 6.0 Hz, 2H, OCH_2_), 3.06 (dd, *J*_1_ = 10.8 Hz, *J*_2_ = 4.0 Hz, 1H, 1/2 COCH_2_), 2.72 (dd, *J*_1_ = 14.4 Hz, *J*_2_ = 2.8 Hz, 1H, 1/2 COCH_2_), 2.54–2.47 (m, 4H, 2 × NCH_2_), 2.44 (t, *J* = 7.2 Hz, 2H, NCH_2_), 1.74–1.62 (m, 8H, 4 × CH_2_), 1.47–1.44 (m, 2H, CH_2_). HR-ESI-MS: Calcd. for C_24_H_29_NO_5_ [M + H]^+^: 412.2079, found: 412.2095.

7–(4-(3,4-dihydroisoquinolin-2(1H)-yl)butoxy)-5-hydroxy-2–(4-hydroxyphenyl)chroman-4-one (**5b**). Compound **3a** was treated with 1,2,3,4-tetrahydroisoquinoline **4b** based on the above procedure to obtain target compound **5b**, light yellow oily matter, 70.1% yield. ^1^H NMR (400 MHz, CDCl_3_) *δ* 12.02 (s, 1H, OH), 7.18 (d, *J* = 8.4 Hz, 2H, 2 × Ar-H), 7.14–7.11 (m, 2H, 2 × Ar-H), 7.08 (t, *J* = 4.0 Hz, Ar-H), 7.03 (t, *J* = 3.6 Hz, 1H, Ar-H), 6.70 (d, *J* = 8.4 Hz, 2H, 2 × Ar-H), 6.01 (d, *J* = 2.0 Hz, 1H, Ar-H), 5.95 (d, *J* = 2.0 Hz, 1H, Ar-H), 5.23 (dd, *J*_1_ = 10.4 Hz, *J*_2_ = 2.8 Hz, 1H, CH), 3.94 (t, *J* = 5.2 Hz, 2H, OCH_2_), 3.73 (s, 2H, phCH_2_), 3.02 (dd, *J*_1_ = 13.2 Hz, *J*_2_ = 4.0 Hz, 1H, 1/2 COCH_2_), 2.94 (t, *J* = 5.6 Hz, 2H, NCH_2_), 2.84 (t, *J* = 5.6 Hz, 2H, NCH_2_), 2.69 (dd, *J*_1_ = 14.4 Hz, *J*_2_ = 2.8 Hz, 1H, 1/2 COCH_2_), 2.62 (t, *J* = 6.8 Hz, 2H, NCH_2_), 1.81–1.76 (m, 4H, 2 × CH_2_). HR-ESI-MS: Calcd. for C_28_H_29_NO_5_ [M + H]^+^: 460.2079, found: 460.2095.

7–(4-(benzyl(ethyl)amino)butoxy)-5-hydroxy-2–(4-hydroxyphenyl)chroman-4-one (**5c**). Compound **3a** was treated with *N*-ethylbenzylamine **4c** based on the above procedure to obtain target compound **5c**, light yellow oily matter, 73.9% yield. ^1^H NMR (400 MHz, CDCl_3_) *δ* 12.02 (s, 1H, OH), 7.34–7.25 (m, 7H, 7 × Ar-H), 6.90 (t, *J* = 8.0 Hz, 2H, 2 × Ar-H), 5.99 (d, *J* = 2.0 Hz, 1H, Ar-H), 5.95 (d, *J* = 2.0 Hz, 1H, Ar-H), 5.27–5.25 (m, 1H, OCH), 3.90–3.87 (m, 2H, OCH_2_), 3.59 (s, 2H, phCH_2_), 3.06 (dd, *J*_1_ = 13.2 Hz, *J*_2_ = 4.0 Hz, 1H, 1/2 COCH_2_), 2.72 (d, *J* = 2.8 Hz, 1H, 1/2 COCH_2_), 2.61–2.54 (m, 4H, 2 × NCH_2_), 1.79–1.66 (m, 4H, 2 × CH_2_), 1.09–1.05 (m, 3H, CH_3_). HR-ESI-MS: Calcd. for C_28_H_31_NO_5_ [M + H]^+^: 462.2236, found: 462.2251.

7–(4-(ethyl(2-methoxybenzyl)amino)butoxy)-5-hydroxy-2–(4-hydroxyphenyl)chroman-4-one (**5d**). Compound **3a** was treated with *N*-(2-methoxybenzyl)ethanamine **4d** based on the above procedure to obtain target compound **5d**, light yellow oily matter, 74.6% yield. ^1^H NMR (400 MHz, CDCl_3_) *δ* 12.03 (s, 1H, OH), 7.38 (d, *J*_1_ = 6.4 Hz, *J*_2_ = 1.2 Hz, 1H, Ar-H), 7.28–7.23 (m, 1H, Ar-H), 7.22 (d, *J* = 8.4 Hz, 2H, 2 × Ar-H), 6.92 (t, *J* = 7.6 Hz, 1H, Ar-H), 6.85 (d, *J* = 8.4 Hz, 1H, Ar-H), 6.82 (d, *J* = 8.8 Hz, 2H, 2 × Ar-H), 5.97 (dd, *J*_1_ = 18.4 Hz, *J*_2_ = 2.0 Hz, 2H, 2 × Ar-H), 5.26 (dd, *J*_1_ = 10.4 Hz, *J*_2_ = 2.8 Hz, 1H, CH), 3.88 (t, *J* = 6.4 Hz, 2H, OCH_2_), 3.78 (s, 3H, OCH_3_), 3.05 (dd, *J*_1_ = 13.2 Hz, *J*_2_ = 4.0 Hz, 1H, 1/2 COCH_2_), 2.74 (d, *J* = 2.8 Hz, 1H, 1/2 COCH_2_), 2.71–2.67 (m, 2H, NCH_2_), 2.66–2.63 (m, 2H, NCH_2_), 1.75–1.71 (m, 4H, 2 × CH_2_), 1.16 (t, *J* = 7.2 Hz, 3H, CH_3_). HR-ESI-MS: Calcd. for C_29_H_33_NO_6_ [M + H]^+^: 492.2341, found: 492.2373.

7–(4-(4-benzylpiperazin-1-yl)butoxy)-5-hydroxy-2–(4-hydroxyphenyl)chroman-4-one (**5e**). Compound **3a** was treated with benzylpiperazine **4e** based on the above procedure to obtain target compound **5e**, light yellow oily matter, 70.3% yield. ^1^H NMR (400 MHz, CDCl_3_) *δ* 12.03 (s, 1H, OH), 7.30–7.24 (m, 7H, 7 × Ar-H), 6.79 (d, *J* = 8.4 Hz, 2H, 2 × Ar-H), 6.00 (d, *J* = 2.0 Hz, 1H, Ar-H), 5.94 (d, *J* = 2.0 Hz, 1H), 5.28 (dd, *J*_1_ = 10.8 Hz, *J*_2_ = 2.4 Hz, 1H, CH), 3.91 (t, *J* = 6.0 Hz, 2H, OCH_2_), 3.53 (s, 2H, phCH_2_), 3.06 (dd, *J*_1_ = 13.2 Hz, *J*_2_ = 4.0 Hz, 1H, 1/2 COCH_2_), 2.73 (dd, *J*_1_ = 14.4 Hz, 1H, 1/2 COCH_2_), 2.68–2.50 (m, 8H, 4 × NCH_2_), 2.46 (t, *J* = 7.2 Hz, 2H, NCH_2_), 1.76–1.71 (m, 2H, CH_2_), 1.70–1.65 (m, 2H, CH_2_). HR-ESI-MS: Calcd. for C_30_H_34_N_2_O_5_ [M + H]^+^: 503.2501, found: 503.2534.

7-((5-(benzyl(ethyl)amino)pentyl)oxy)-5-hydroxy-2–(4-hydroxyphenyl)chroman-4-one (**5f**). Compound **3b** was treated with *N*-ethylbenzylamine **4c** based on the above procedure to afford target compound **5f**, light yellow oily matter, 68.7% yield. ^1^H NMR 12.01 (s, 1H, OH), 7.36–7.27 (m, 7H, 7 × Ar-H), 6.90–6.87 (m, 2H, 2 × Ar-H), 5.99 (d, *J* = 2.0 Hz, 1H, Ar-H), 5.94 (d, *J* = 2.0 Hz, 1H, Ar-H), 5.27 (dd, *J*_1_ = 10.8 Hz, *J*_2_ = 2.4 Hz, 1H, CH), 3.92–3.88 (m, 2H, OCH_2_), 3.55 (s, 2H, phCH_2_), 3.06 (dd, *J*_1_ = 13.2 Hz, *J*_2_ = 4.0 Hz, 1H, 1/2 COCH_2_), 2.71 (d, *J* = 2.8 Hz, 1H, 1/2 COCH_2_), 2.59–2.53 (m, 4H, 2 × NCH_2_), 1.77–1.65 (m, 4H, 2 × CH_2_), 1.51–1.46 (m, 2H, CH_2_), 1.14–1.09 (m, 3H, CH_3_). HR-ESI-MS: Calcd. for C_29_H_33_NO_5_ [M + H]^+^: 476.2393, found: 476.2417.

7-((5–(4-benzylpiperidin-1-yl)pentyl)oxy)-5-hydroxy-2–(4-hydroxyphenyl)chroman-4-one (**5g**). Compound **3b** was treated with benzylpiperidine **4f** based on the above procedure to afford target compound **5g**, light yellow oily matter, 60.7% yield. ^1^H NMR 12.00 (s, 1H, OH), 7.26–7.16 (m, 4H, 4 × Ar-H), 7.20–7.16 (m, 1H, Ar-H), 7.11 (d, *J* = 7.2 Hz, 2H, 2 × Ar-H), 6.87 (d, *J* = 8.4 Hz, 2H, 2 × Ar-H), 5.98 (d, *J* = 2.0 Hz, 1H, Ar-H), 5.93 (d, *J* = 2.0 Hz, 1H, Ar-H), 5.25 (dd, *J*_1_ = 10.4 Hz, *J*_2_ = 2.8 Hz, 1H, OCH), 3.85 (t, *J* = 6.4 Hz, 2H, OCH_2_), 3.19 (d, *J* = 11.6 Hz, 2H, phCH_2_), 3.05 (dd, *J*_1_ = 13.2 Hz, *J*_2_ = 4.0 Hz, 1H, 1/2 COCH_2_), 2.71 (dd, *J*_1_ = 14.0 Hz, *J*_2_ = 2.8 Hz, 1H, 1/2 COCH_2_), 2.58–2.52 (m, 4H, 2 × NCH_2_), 2.18 (t, *J* = 7.2 Hz, 2H, NCH_2_), 1.73–1.64 (m, 6H, 3 × CH_2_), 1.58–1.42 (m, 2H, CH_2_), 1.43–1.35 (m, 2H, CH_2_). HR-ESI-MS: Calcd. for C_32_H_37_NO_5_ [M + H]^+^: 516.2705, found: 516.2741.

7-((6-(benzyl(ethyl)amino)hexyl)oxy)-5-hydroxy-2–(4-hydroxyphenyl)chroman-4-one (**5h**). Compound **3c** was treated with *N*-ethylbenzylamine **4c** based on the above procedure to afford target compound **5h**, light yellow oily matter, 62.4% yield. ^1^H NMR (400 MHz, CDCl_3_) *δ* 12.01 (s, 1H, OH), 7.35–7.23 (m, 7H, 7 × Ar-H), 6.88 (d, *J* = 8.4 Hz, 2H, 2 × Ar-H), 6.00 (d, *J* = 2.0 Hz, 1H, Ar-H), 5.94 (d, *J* = 2.0 Hz, 1H, Ar-H), 5.27 (dd, *J*_1_ = 10.4 Hz, *J*_2_ = 2.8 Hz, 1H, CH), 3.91 (t, *J* = 6.4 Hz, 2H, OCH_2_), 3.78–3.73 (m, 2H, phCH_2_), 3.05 (dd, *J*_1_ = 13.2 Hz, *J*_2_ = 4.0 Hz, 1H, 1/2 COCH_2_), 2.72 (d, *J* = 2.8 Hz, 1H, 1/2 COCH_2_), 2.60–2.54 (m, 4H, 2 × NCH_2_), 1.87–1.73 (m, 4H, 2 × CH_2_), 1.50–1.43 (m, 4H, 2 × CH_2_), 1.13–1.07 (m, 3H, CH_3_). HR-ESI-MS: Calcd. for C_30_H_35_NO_5_ [M + H]^+^: 490.2549, found: 490.2573.

7-((10-(benzyl(ethyl)amino)decyl)oxy)-5-hydroxy-2–(4-hydroxyphenyl)chroman-4-one (**5i**). Compound **3d** was treated with *N*-ethylbenzylamine **4c** based on the above procedure to afford target compound **5i**, light yellow oily matter, 52.6% yield. ^1^H NMR (400 MHz, CDCl_3_) *δ* 12.01 (s, 1H, OH), 7.37–7.25 (m, 7H, 7 × Ar-H), 6.88 (d, *J* = 8.4 Hz, 2H, 2 × Ar-H), 5.99 (d, *J* = 2.0 Hz, 1H, Ar-H), 5.94 (d, *J* = 2.0 Hz, 1H, Ar-H), 5.28 (dd, *J*_1_ = 10.4 Hz, *J*_2_ = 2.8 Hz, 1H, CH), 3.91 (t, *J* = 6.4 Hz, 2H, OCH_2_), 3.80–3.75 (m, 2H, phCH_2_), 3.06 (dd, *J*_1_ = 13.2 Hz, *J*_2_ = 4.0 Hz, 1H, 1/2 COCH_2_), 2.70 (d, *J* = 2.8 Hz, 1H, 1/2 COCH_2_), 2.61–2.52 (m, 4H, 2 × NCH_2_), 1.76–1.70 (m, 4H, 2 × CH_2_), 1.45–1.39 (m, 4H, 2 × CH_2_), 1.33–1.25 (m, 8H, 4 × CH_2_), 1.14–1.06 (m, 3H, CH_3_). HR-ESI-MS: Calcd. for C_34_H_43_NO_5_ [M + H]^+^: 546.3175, found: 546.3198.

7-((12-(benzyl(ethyl)amino)dodecyl)oxy)-5-hydroxy-2–(4-hydroxyphenyl)chroman-4-one (**5j**). Compound **3e** was treated with *N*-ethylbenzylamine **4c** based on the above procedure to afford target compound **5j**, light yellow oily matter, 51.7% yield. ^1^H NMR (400 MHz, CDCl_3_) *δ* 12.03 (s, 1H, OH), 7.38–7.24 (m, 7H, 7 × Ar-H), 6.90–6.88 (m, 2H, 2 × Ar-H), 6.00 (d, *J* = 2.0 Hz, 1H, Ar-H), 5.94 (d, *J* = 2.0 Hz, 1H, Ar-H), 5.29 (dd, *J*_1_ = 10.4 Hz, *J*_2_ = 2.8 Hz, 1H, CH), 3.93 (t, *J* = 6.4 Hz, 2H, OCH_2_), 3.81–3.78 (brs, 2H, phCH_2_), 3.06 (dd, *J*_1_ = 13.2 Hz, *J*_2_ = 4.0 Hz, 1H, 1/2 COCH_2_), 2.71 (d, *J* = 2.8 Hz, 1H, 1/2 COCH_2_), 2.60–2.49 (m, 4H, 2 × NCH_2_), 1.75–1.67 (m, 4H, 2 × CH_2_), 1.46–1.36 (m, 4H, 2 × CH_2_), 1.34–1.20 (m, 12H, 6 × CH_2_), 1.11–1.07 (m, 3H, CH_3_). HR-ESI-MS: Calcd. for C_36_H_47_NO_5_ [M + H]^+^: 574.3488, found: 574.3521.

#### 4.1.3. Synthesis of intermediates 6a–6c

The synthesis of intermediates **6a–6c** could reference our previous work. Briefly, the starting material **1** (10.0 mmol) was reacted with 55.0 mmol dibromides (**2a, 2b** and **2d**, respectively) in the presence of 30.0 mmol K_2_CO_3_ in CH_3_CN at 65 °C for 10–15 h under an argon atmosphere. After complete reaction, the solvent was treated under universal method to afford the crude product, which was dissolved in CH_2_Cl_2_, washed with water (2 × 30 ml). The combined organic phases were washed with saturated aqueous NaCl (50 ml) and dried over Na_2_SO_4_. The solvent was evaporated to obtain a crude mixture, which was further purified on a silica gel chromatography using mixtures of petroleum/acetone = 50:1 as mobile phase to get the key intermediates **6a–6c**.

7–(3-bromopropoxy)-2–(4-(3-bromopropoxy)phenyl)-5-hydroxychroman-4-one (**6a**). The starting material **1** was treated with 1,3-dibromopropane **2a** based on the above procedure to obtain compound **6a**, white solid, 32.8% yield. ^1^H NMR (400 MHz, CDCl_3_) δ ^1^H NMR (400 MHz, CDCl_3_) δ 12.01 (s, 1H, OH), 7.38 (d, *J* = 8.6 Hz, 2H, 2 × Ar-H), 6.96 (d, *J* = 8.7 Hz, 2H, 2 × Ar-H), 6.05 (dd, *J* = 11.4, 2.2 Hz, 2H, 2 × Ar-H), 5.37 (dd, *J* = 12.9, 2.8 Hz, 1H, OCH), 4.12 (dd, *J* = 12.6, 5.9 Hz, 4H, 2 ×OCH_2_), 3.59 (dt, *J* = 20.1, 6.4 Hz, 4H, 2 ×BrCH_2_), 3.09 (dd, *J* = 17.1, 13.0 Hz, 1H, 1/2COCH_2_), 2.79 (dd, *J* = 17.1, 3.0 Hz, 1H, 1/2COCH_2_), 2.38–2.25 (m, 4H, 2 ×CH_2_). ^13 ^C NMR (100 MHz, CDCl_3_) δ 196.1, 167.1, 164.2, 163.0, 159.3, 130.8, 127.9 (2 C), 115.0 (2 C), 103.4, 95.7, 94.7, 79.1, 65.9, 65.5, 43.3, 32.4, 32.0, 30.0, 29.6.

7–(4-bromobutoxy)-2–(4-(4-bromobutoxy)phenyl)-5-hydroxychroman-4-one (**6b**). The starting material **1** was treated with 1,4-dibromobutane **2b** based on the above procedure to obtain compound **6b**, colourless oily matter, 43.9% yield. ^1^H NMR (400 MHz, CDCl_3_) *δ* 12.02 (s, 1H, OH), 7.37 (d, *J* = 8.4 Hz, 2H, 2 × Ar-H), 6.94 (d, *J* = 8.8 Hz, 2H, 2 × Ar-H), 6.04 (d, *J* = 2.4 Hz, 1H, Ar-H), 6.02 (d, *J* = 2.4 Hz, 1H, Ar-H), 5.36 (dd, *J*_1_ = 10.0 Hz, *J*_2_ = 2.8 Hz, 1H, CH), 4.02 (t, *J* = 6.0 Hz, 2H, OCH_2_), 4.00 (t, *J* = 6.0 Hz, 2H, OCH_2_), 3.50 (t, *J* = 6.4 Hz, 2H, BrCH_2_), 3.47 (t, *J* = 6.8 Hz, 2H, BrCH_2_), 3.10 (dd, *J*_1_ = 12.8 Hz, *J*_2_ = 8.0 Hz, 1H, 1/2 CH_2_), 2.79 (dd, *J*_1_ = 14.4 Hz, *J*_2_ = 2.8 Hz, 1H, 1/2 CH_2_), 2.10–2.01 (m, 4H, 2 × CH_2_), 1.99–1.91 (m, 4H, 2 × CH_2_).

7-((6-bromohexyl)oxy)-2–(4-((6-bromohexyl)oxy)phenyl)-5-hydroxychroman-4-one (**6c**). The starting material **1** was treated with 1,6-dibromohexane **2d** based on the above procedure to obtain compound **6c**, colourless oily matter, 30.4% yield. ^1^H NMR (400 MHz, CDCl_3_) *δ* ^1^H NMR 12.02 (s, 1H, OH), 7.36 (d, *J* = 8.4 Hz, 2H, 2 × Ar-H), 6.94 (d, *J* = 8.4 Hz, 2H, 2 × Ar-H), 6.03 (d, *J* = 9.2 Hz, 2H, 2 × Ar-H), 5.36 (dd, *J*_1_ = 11.2 Hz, *J*_2_ = 1.6 Hz, 1H, OCH), 4.00–3.94 (m, 4H, 2 × OCH_2_), 3.45–3.40 (m, 4H, 2 × BrCH_2_), 3.09 (dd, *J*_1_ = 13.2 Hz, *J*_2_ = 4.0 Hz, 1H, 1/2 COCH_2_), 2.78 (dd, *J*_1_ = 14.8 Hz, *J*_2_ = 2.4 Hz, 1H, 1/2 COCH_2_), 1.92–1.86 (m, 4H, 2 × CH_2_), 1.83–1.76 (m, 4H, 2 × CH_2_), 1.52–1.49 (m, 8H, 4 × CH_2_).

#### 4.1.4. Synthesis of 7,4’-*O*-modified naringenin derivatives 7a–7k

The synthesis of 7,4′-*O*-modified naringenin derivatives **7a–7k** could reference our previous work. Briefly, the key intermediates **6a–6c** (1.0 mmol) were reacted with secondary amines **4a–4h** (3.0 mmol), respectively, in the presence of K_2_CO_3_ (3.5 mmol) at 65 °C for 8–12 h. After complete reaction, the solvent was treated by universal method to afford the crude product, which was further purified on a silica gel chromatography using mixtures of petroleum/acetone = 30:1 as mobile phase to get the target 7,4′-*O*-modified naringenin derivatives **7a–7k**.

7–(3-(3,4-dihydroisoquinolin-2(1H)-yl)propoxy)-2–(4-(3–(3,4-dihydroisoquinolin-2(1H)-yl)propoxy)phenyl)-5-hydroxychroman-4-one (**7a**). Compound **6a** was treated with 1,2,3,4-tetrahydroisoquinoline **4b** based on the above procedure to afford target compound **7a**, light yellow oily matter, 40.9% yield. ^1^H NMR (400 MHz, CDCl_3_) δ 12.04 (s, 1H, OH), 7.36 (d, *J* = 8.3 Hz, 2H, 2 × Ar-H), 7.17 − 7.03 (m, 8H, 8 × Ar-H), 6.96 (d, *J* = 8.4 Hz, 2H, 2 × Ar-H), 6.07 (d, *J* = 9.3 Hz, 2H, 2 × Ar-H), 5.43–5.31 (m, 1H, OCH), 4.10–4.07 (m, 4H, 2 × OCH_2_), 3.09–3.06 (m, 4H, 2 × NCH_2_), 2.87–2.65 (m, 10H, COCH_2_ + 2 × CH_2_ + 2 × NCH_2_), 2.13–1.95 (m, 4H, 2 × NCH_2_), 1.34 (d, *J* = 8.0 Hz, 4H, 2 × CH_2_). HR-ESI-MS: Calcd. for C_39_H_42_N_2_O_5_ [M + H]^+^: 619.3127, found: 619.3159.

7–(3-(benzyl(ethyl)amino)propoxy)-2–(4-(3-(benzyl(ethyl)amino)propoxy)phenyl)-5-hydroxychroman-4-one (**7b**). Compound **6a** was treated with *N*-ethylbenzylamine **4c** based on the above procedure to afford target compound **7b**, light yellow oily matter, 57.2% yield. ^1^H NMR (400 MHz, CDCl_3_) δ 12.06 (s, 1H, OH), 7.33–7.28 (m, 12H, 12 × Ar-H), 6.90 (dd, *J* = 8.2, 4.4 Hz, 2H, 2 × Ar-H), 6.04–5.91 (m, 2H, 2 × Ar-H), 5.36 (d, *J* = 15.3 Hz, 1H, OCH), 4.09–3.91 (m, 4H, 2 × OCH_2_), 3.60 (s, 2H, phCH_2_), 3.58 (s, 2H, phCH_2_), 2.77 (dd, *J*_1_ = 14.8 Hz, *J*_2_ = 2.4 Hz, 1H, 1/2 COCH_2_), 2.59–2.56 (m, 8H, 4 × NCH_2_), 2.00–1.91 (m, 4H, 2 × CH_2_), 1.07–1.04 (m, 6H, 2 × CH_3_). HR-ESI-MS: Calcd. for C_39_H_42_N_2_O_5_ [M + H]^+^: 623.3440, found: 623.3466.

7–(3-(4-benzylpiperidin-1-yl)propoxy)-2–(4-(3–(4-benzylpiperidin-1-yl)propoxy)phenyl)-5-hydroxychroman-4-one (**7c**). Compound **6a** was treated with benzylpiperidine **4f** based on the above procedure to afford target compound **7c**, light yellow oily matter, 47.1% yield. ^1^H NMR (400 MHz, CDCl_3_) δ 12.02 (s, 1H, OH), 7.35 (d, *J* = 8.2 Hz, 2H, 2 × Ar-H), 7.26 (s, 4H, 4 × Ar-H), 7.17 (dd, *J* = 22.2, 5.7 Hz, 6H, 6 × Ar-H), 6.93 (d, *J* = 8.1 Hz, 2H, 2 × Ar-H), 6.04 (d, *J* = 8.8 Hz, 2H, 2 × Ar-H), 5.40–5.33 (m, 1H, OCH), 4.03–4.00 (m, 4H, 2 × OCH_2_), 3.09–3.07 (m, 1H, CH), 3.00–2.73 (m, 6H, 2 × CH_2_ + NCH_2_), 2.54 (t, *J* = 6.8 Hz, 6H, 3 × NCH_2_), 2.50–2.43 (m, 2H, NCH_2_), 1.98–1.95 (m, 8H, NCH_2_ + 3 × CH_2_), 1.67–1.65 (m, 4H, 2 × CH_2_), 1.54 (d, *J* = 7.4 Hz, 2H, 2 × CH), 1.42 − 1.21 (m, 6H, 3 × CH_2_). ^13 ^C NMR (100 MHz, CDCl_3_) δ 196.1, 167.5, 164.2, 163.0, 159.5, 140.7, 140.6, 130.5, 129.2 (4 C), 128.3 (2 C), 128.3 (2 C), 127.8 (2 C), 125.9, 125.9, 114.9 (2 C), 103.2, 95.7, 94.6, 67.0, 66.6, 55.6, 55.3, 54.0 (4 C), 43.3, 43.2 (2 C), 43.2 (2 C), 38.0, 37.9, 32.1 (2 C), 32.0 (2 C), 26.8, 26.6. HR-ESI-MS: Calcd. for C_45_H_54_N_2_O_5_ [M + H]^+^: 703.4066, found: 703.4092.

5-hydroxy-7–(3-(4-phenylpiperidin-1-yl)propoxy)-2–(4-(3–(4-phenylpiperidin-1-yl)propoxy)phenyl)chroman-4-one (**7d**). Compound **6a** was treated with 4-phenylpiperidine **4g** based on the above procedure to afford target compound **7d**, light yellow oily matter, 56.8% yield. ^1^H NMR (400 MHz, CDCl_3_) δ 12.03 (s, 1H, OH), 7.37 (d, *J* = 8.4 Hz, 2H, 2 × Ar-H), 7.29 (d, *J* = 7.0 Hz, 4H, 4 × Ar-H), 7.26 − 7.20 (m, 6H, 6 × Ar-H), 6.95 (d, *J* = 8.4 Hz, 2H, 2 × Ar-H), 6.07 (d, *J* = 7.2 Hz, 2H, 2 × Ar-H), 5.33 (t, *J* = 16.5 Hz, 1H, CH), 4.11 − 3.99 (m, 4H, 2 × OCH_2_), 3.10–3.08 (m, 6H, COCH_2_, 2 × CH + NCH_2_), 2.80–2.78 (m, 2H, NCH_2_), 2.63–2.47 (m, 6H, 3 × NCH_2_), 2.15–2.04 (m, 6H, NCH_2_ + 2 × CH_2_), 1.84 (d, *J* = 8.6 Hz, 8H, 4 × CH_2_). ^13 ^C NMR (100 MHz, CDCl_3_) δ 196.1, 167.6, 164.2, 163.0, 159.6, 146.3, 146.3, 130.5, 128.6 (2 C), 128.5 (2 C), 127.8 (2 C), 127.0 (4 C), 126.3, 126.3, 114.9 (2 C), 103.2, 95.7, 94.7, 79.1, 67.0, 66.6, 55.6, 55.3, 54.5 (4 C), 43.3, 42.7, 42.7, 33.5 (2 C), 33.4 (2 C), 26.9, 26.7. HR-ESI-MS: Calcd. for C_43_H_50_N_2_O_5_ [M + H]^+^: 675.3753, found: 675.3769.

7–(3-(4-ethylpiperazin-1-yl)propoxy)-2–(4-(3–(4-ethylpiperazin-1-yl)propoxy)phenyl)-5-hydroxychroman-4-one (**7e**). Compound **6a** was treated with 1-ethylpiperazine **4h** based on the above procedure to afford target compound **7e**, light yellow oily matter, 52.3% yield. ^1^H NMR (400 MHz, CDCl_3_) δ 12.04 (s, 1H, OH), 7.31 (d, *J* = 8.4 Hz, 2H, 2 × Ar-H), 6.88 (d, *J* = 8.4 Hz, 2H, 2 × Ar-H), 5.98 (d, *J* = 6.0 Hz, 2H, 2 × Ar-H), 5.30 (d, *J* = 10.8 Hz, 1H, OCH), 3.96 (d, *J* = 4.6 Hz, 4H, 2 × OCH_2_), 3.54 (s, 1H, 1/2CH_2_), 3.04 (d, *J* = 3.9 Hz, 1H, 1/2CH_2_), 2.80 − 2.22 (m, 24H, 12 × NCH_2_), 1.93 (dd, *J* = 13.6, 7.0 Hz, 4H, 2 × CH_2_), 1.07–1.05 (m, 6H, 2 × CH_3_). HR-ESI-MS: Calcd. for C_33_H_48_N_4_O_5_ [M + H]^+^: 581.3658, found: 581.3679.

7–(4-(3,4-dihydroisoquinolin-2(1H)-yl)butoxy)-2–(4-(4–(3,4-dihydroisoquinolin-2(1H)-yl)butoxy)phenyl)-5-hydroxychroman-4-one (**7f**). Compound **6b** was treated with 1,2,3,4-tetrahydroisoquinoline **4b** based on the above procedure to afford target compound **7f**, light yellow oily matter, 61.3% yield. ^1^H NMR (400 MHz, CDCl_3_) *δ* 12.04 (s, 1H, OH), 7.36 (d, *J* = 8.8 Hz, 2H, 2 × Ar-H), 7.13–7.10 (m, 6H, 6 × Ar-H), 7.04–7.00 (m, 2H, 2 × Ar-H), 6.95 (d, *J* = 8.4 Hz, 2H, 2 × Ar-H), 6.04 (d, *J* = 10.0 Hz, 2H, 2 × Ar-H), 5.36 (dd, *J*_1_ = 10.8 Hz, *J*_2_ = 2.0 Hz, 1H, OCH), 4.04–3.99 (m, 4H, 2 × OCH_2_), 3.67 (s, 2H, phCH_2_), 3.65 (s, 2H, phCH_2_), 3.10 (dd, *J*_1_ = 13.2 Hz, *J*_2_ = 3.6 Hz, 1H, 1/2 COCH_2_), 2.93–2.90 (m, 4H, 2 × NCH_2_), 2.80–2.74 (m, 5H, 2 × NCH_2_ + 1/2 COCH_2_), 2.63–2.56 (m, 4H, 2 × NCH_2_), 1.93–1.69 (m, 8H, 4 × CH_2_). HR-ESI-MS: Calcd. for C_41_H_46_N_2_O_5_ [M + H]^+^: 647.3440, found: 647.3461.

7–(4-(ethyl(2-methoxybenzyl)amino)butoxy)-2–(4-(4-(ethyl(2-methoxybenzyl)amino)butoxy)phenyl)-5-hydroxychroman-4-one (**7h**). Compound **6b** was treated with *N*-(2-methoxybenzyl)ethanamine **4d** based on the above procedure to afford target compound **7h**, light yellow oily matter, 43.9% yield. ^1^H NMR (400 MHz, CDCl_3_) *δ* ^1^H NMR 12.02 (s, 1H, OH), 7.52 (d, *J* = 8.0 Hz, 1H, Ar-H), 7.48 (d, *J* = 7.2 Hz, 1H, Ar-H), 7.36 (d, *J* = 8.4 Hz, 2H, 2 × Ar-H), 7.31–7.24 (m, 2H, 2 × Ar-H), 6.96 (t, *J* = 7.6 Hz, 2H, 2 × Ar-H), 6.91 (d, *J* = 8.8 Hz, 2H, 2 × Ar-H), 6.89 (d, *J* = 3.2 Hz, 1H, Ar-H), 6.87 (d, *J* = 3.2 Hz, 1H, Ar-H), 6.01 (dd, *J*_1_ = 6.4 Hz, *J*_2_ = 2.4 Hz, 2H, 2 × Ar-H), 5.36 (dd, *J*_1_ = 10.0 Hz, *J*_2_ = 2.8 Hz, 1H, OCH), 3.99–3.93 (m, 4H, 2 × OCH_2_), 3.87 (s, 2H, phCH_2_), 3.84 (s, 3H, OCH_3_), 3.83 (s, 3H, OCH_3_), 3.81 (s, 2H, phCH_2_), 3.09 (dd, *J*_1_ = 13.2 Hz, *J*_2_ = 4.0 Hz, 1H, 1/2 COCH_2_), 2.79 (dd, *J*_1_ = 14.0 Hz, *J*_2_ = 3.2 Hz, 1H, 1/2 COCH_2_), 2.78–2.63 (m, 8H, 4 × NCH_2_)1.84–1.81 (m, 4H, 2 × CH_2_), 1.78–1.76 (m, 4H, 2 × CH_2_), 1.24–1.16 (m, 6H, 2 × CH_3_). HR-ESI-MS: Calcd. for C_43_H_54_N_2_O_7_ [M + H]^+^: 711.3965, found: 711.3987.

7–(4-(4-benzylpiperazin-1-yl)butoxy)-2–(4–(4-(4-benzylpiperazin-1-yl)butoxy)phenyl)-5-hydroxychroman-4-one (**7i**). Compound **6b** was treated with benzyl piperazine **4e** based on the above procedure to afford target compound **7i**, light yellow oily matter, 51.9% yield. ^1^H NMR (400 MHz, CDCl_3_) *δ* 12.03 (s, 1H, OH), 7.35–7.22 (m, 12H, 12 × Ar-H), 6.92 (d, *J* = 8.4 Hz, 2H, 2 × Ar-H), 6.02 (dd, *J*_1_ = 9.2 Hz, *J*_2_ = 2.4 Hz, 2H, 2 × Ar-H), 5.33 (dd, *J*_1_ = 10.4 Hz, *J*_2_ = 2.8 Hz, 1H, OCH), 3.99–3.94 (m, 4H, 2 × OCH_2_), 3.51 (s, 2H, phCH_2_), 3.50 (s, 2H, phCH_2_), 3.08 (dd, *J*_1_ = 12.8 Hz, *J*_2_ = 4.4 Hz, 1H, 1/2 COCH_2_), 2.76 (dd, *J*_1_ = 14.4 Hz, *J*_2_ = 2.8 Hz, 1H, 1/2 COCH_2_), 2.49–2.30 (m, 20H, 10 × NCH_2_), 1.83–1.73 (m, 4H, 2 × CH_2_), 1.71–1.58 (m, 4H, 2 × CH_2_). HR-ESI-MS: Calcd. for C_45_H_56_N_4_O_5_ [M + H]^+^: 733.4284, found: 733.4297.

7-((6-(benzyl(ethyl)amino)hexyl)oxy)-2–(4-((6-(benzyl(ethyl)amino)hexyl)oxy)phenyl)-5-hydroxychroman-4-one (**7k**). Compound **6c** was treated with *N*-ethylbenzylamine **4c** based on the above procedure to afford target compound **7k**, light yellow oily matter, 40.1% yield. ^1^H NMR (400 MHz, CDCl_3_) *δ* ^1^H NMR 12.02 (s, 1H, OH), 7.34–7.25 (m, 12H,12 × Ar-H), 6.92 (d, *J* = 8.4 Hz, 2H, 2 × Ar-H), 6.04 (d, *J* = 10.0 Hz, 2H, 2 × Ar-H), 5.36 (dd, *J*_1_ = 11.2 Hz, *J*_2_ = 2.0 Hz, 1H, OCH), 3.98–3.93 (m, 4H, 2 × OCH_2_), 3.62 (s, 2H, phCH_2_), 3.59 (s, 2H, phCH_2_), 3.09 (dd, *J*_1_ = 13.2 Hz, *J*_2_ = 4.0 Hz, 1H, 1/2 COCH_2_), 2.78 (dd, *J*_1_ = 14.8 Hz, *J*_2_ = 2.4 Hz, 1H, 1/2 COCH_2_), 2.58–2.54 (m, 8H, 4 × NCH_2_), 1.95–1.84 (m, 4H, 2 × CH_2_), 1.81–1.73 (m, 4H, 2 × CH_2_), 1.50–1.43 (m, 8H, 4 × CH_2_), 1.09–1.03 (m, 6H, 2 × CH_3_). HR-ESI-MS: Calcd. for C_45_H_58_N_2_O_5_ [M + H]^+^: 707.4379, found: 707.4413.

#### 4.1.5. Synthesis of intermediates 9a–9d

The synthesis of intermediates **9a–9d** could reference our previous work. Briefly, the starting material **8** (10.0 mmol) was treated with 55.0 mmol dibromides (**2a–2d**) in the presence of 30.0 mmol K_2_CO_3_ in CH_3_CN at 65 °C for 10–15 h. The reaction was monitored using TLC, after the reaction completed, the mixture was treated through under universal method to afford the crude product, which was further purified on a silica gel chromatography using mixtures of petroleum/ethyl acetate = 50:1 as mobile phase to get the key intermediates **9a–9d**.

7–(3-bromopropoxy)-2–(4-(3-bromopropoxy)phenyl)-5-hydroxy-4H-chromen-4-one (**9a**). The starting material **8** was treated with 1,3-dibromopropane **2a** based on the above procedure to obtain compound **9a**, colourless oily matter, 56.9% yield. ^1^H NMR (400 MHz, CDCl_3_) *δ* 12.70 (s, 1H, OH), 7.75 (d, *J* = 8.8 Hz, 2H, 2 × Ar-H), 6.94 (d, *J* = 8.8 Hz, 2H, 2 × Ar-H), 6.50 (s, 1H, Ar-H), 6.42 (d, *J* = 2.4 Hz, 1H, Ar-H), 6.29 (d, *J* = 2.0 Hz, 1H, Ar-H), 4.13–4.10 (m, 4H, 2 × BrCH_2_), 3.57–3.52 (m, 4H, 2 × OCH_2_), 2.31–2.71 (m, 4H, 2 × CH_2_).

7-((5-bromopentyl)oxy)-2–(4-((5-bromopentyl)oxy)phenyl)-5-hydroxy-4H-chromen-4-one (**9c**). The starting material **8** was treated with 1,5-dibromopentane **2c** based on the above procedure to obtain compound **9c**, colourless oily matter, 52.7% yield. ^1^H NMR (400 MHz, CDCl_3_) *δ* 12.81 (s, 1H, OH), 7.82 (d, *J* = 8.8 Hz, 2H, 2 × Ar-H), 6.99 (d, *J* = 8.8 Hz, 2H, 2 × Ar-H), 6.57 (s, 1H, Ar-H), 6.47 (d, *J* = 2.0 Hz, 1H, Ar-H), 6.34 (d, *J* = 2.0 Hz, 1H), 4.07–4.02 (m, 4H, 2 × OCH_2_), 3.46 (t, *J* = 6.8 Hz, 4H, 2 × BrCH_2_), 1.98–1.93 (m, 4H, 2 × CH_2_), 1.88–1.83 (m, 4H, 2 × CH_2_), 1.68–1.64 (m, 4H, 2 × CH_2_).

#### 4.1.6. Synthesis of 7,4’-*O*-modified naringenin derivatives 10a–10v

The synthesis of 7,4′-*O*-modified naringenin derivatives **7a–7k** could reference our previous work. Briefly, the key intermediates **9a–9d** (1.0 mmol) were reacted with secondary amines **4a–4j** (3.0 mmol), respectively, in the presence of K_2_CO_3_ (3.5 mmol) at 65 °C for 8–12 h. After complete reaction, the mixture was treated through the universal method to afford the crude product, which was further purified on a silica gel chromatography using mixtures of CH_2_Cl_2_/acetone = 50:1 as mobile phase to get the target 7,4′-O-modified naringenin derivatives **10a–10v**.

7–(3-(3,4-dihydroisoquinolin-2(1H)-yl)propoxy)-2–(4-(3–(3,4-dihydroisoquinolin-2(1H)-yl)propoxy)phenyl)-5-hydroxy-4H-chromen-4-one (**10a**). Compound **9a** was treated with 1,2,3,4-tetrahydroisoquinoline **4b** based on the above procedure to afford target compound **10a**, light yellow oily matter, 41.3% yield. ^1^H NMR (400 MHz, CDCl_3_) δ 12.72 (s, 1H, OH), 7.74 (d, *J* = 8.8 Hz, 2H, 2 × Ar-H), 7.21 (t, *J* = 5.2 Hz, 4H, 4 × Ar-H), 7.13 (d, *J* = 7.0 Hz, 2H, 2 × Ar-H), 7.06 (d, *J* = 7.3 Hz, 4H, 4 × Ar-H), 6.91 (d, *J* = 8.8 Hz, 2H, 2 × Ar-H), 6.49 (s, 1H, Ar-H), 6.40 (d, *J* = 1.9 Hz, 1H, C = CH), 6.26 (d, *J* = 1.9 Hz, 1H, Ar-H), 4.02 (t, *J* = 5.3 Hz, 4H, 2 × OCH_2_), 3.00 (s, 4H, 2 × NCH_2_), 2.56 (dd, *J* = 15.6, 9.2 Hz, 4H, 2 × NCH_2_), 2.03–2.00 (m, 8H, 2 × CH_2_, 2 × NCH_2_), 1.53–1.49 (m, 4H, 2 × CH_2_). HR-ESI-MS: Calcd. for C_39_H_40_N_2_O_5_ [M + H]^+^: 617.2971, found: 617.2986.

7–(3-(benzyl(ethyl)amino)propoxy)-2–(4-(3-(benzyl(ethyl)amino)propoxy)phenyl)-5-hydroxy-4H-chromen-4-one (**10b**). Compound **9a** was treated with *N*-ethylbenzylamine **4c** based on the above procedure to afford target compound **10b**, light yellow oily matter, 56.8% yield. ^1^H NMR (400 MHz, CDCl_3_) δ ^1^H NMR (400 MHz, CDCl_3_) δ 12.80 (d, *J* = 7.8 Hz, 1H, OH), 7.80 (d, *J* = 8.8 Hz, 2H, 2 × Ar-H), 7.29 (dt, *J* = 7.7, 5.8 Hz, 8H, 8 × Ar-H), 7.24–7.18 (m, 2H, 2 × Ar-H), 6.95 (d, *J* = 8.8 Hz, 2H, 2 × Ar-H), 6.56 (s, 1H, Ar-H), 6.42 (d, *J* = 2.0 Hz, 1H, C = CH), 6.30 (d, *J* = 1.9 Hz, 1H, Ar-H), 4.06 (d, *J* = 3.2 Hz, 4H, 2 × OCH_2_), 3.61–3.58 (m, 4H, 2 × NCH_2_), 2.64–2.48 (m, 8H, 4 × NCH_2_), 1.97–1.94 (m, 4H, 2 × CH_2_), 1.06 (t, *J* = 7.0 Hz, 6H, 2 × CH_3_). ^13 ^C NMR (100 MHz, CDCl_3_) δ 182.5, 165.0, 164.1, 162.2, 162.1, 157.8, 139.9, 139.9, 128.9 (2 C), 128.9 (2 C), 128.3 (2 C), 128.3 (2 C), 128.0 (2 C), 126.9 (2 C), 123.4, 115.1 (2 C), 105.5, 104.3, 98.6, 93.1, 77.5, 77.2, 76.8, 66.8, 66.5, 58.4, 58.3, 49.5, 49.4, 47.6, 47.6, 27.0, 27.0, 12.0, 12.0. HR-ESI-MS: Calcd. for C_39_H_44_N_2_O_5_ [M + H]^+^: 621.3284, found: 621.3297.

7–(3-(4-benzylpiperidin-1-yl)propoxy)-2–(4-(3–(4-benzylpiperidin-1-yl)propoxy)phenyl)-5-hydroxy-4H-chromen-4-one (**10c**). Compound **9a** was treated with benzylpiperidine **4f** based on the above procedure to afford target compound **10c**, light yellow oily matter, 43.6% yield. ^1^H NMR (400 MHz, CDCl_3_) δ 12.71 (s, 1H, OH), 7.73 (d, *J* = 8.3 Hz, 2H, 2 × Ar-H), 7.21 − 7.17 (m, 4H, 4 × Ar-H), 7.12 (d, *J* = 7.2 Hz, 2H, 2 × Ar-H), 7.06 (d, *J* = 7.4 Hz, 4H, 4 × Ar-H), 6.90 (d, *J* = 8.3 Hz, 2H, 2 × Ar-H), 6.47 (s, 1H, Ar-H), 6.39 (s, 1H, C = CH), 6.25 (s, 1H, Ar-H), 4.01 (s, 4H, 2 × OCH_2_), 3.01 (s, 4H, 2 × NCH_2_), 2.56 (t, *J* = 13.8 Hz, 4H, 2 × NCH_2_), 2.11–2.08 (m, 10H, 2 × NCH_2_ + 2 × CH_2_ + 2 × CH), 1.51–1.47 (m, 4H, 2 × CH_2_), 1.22–1.19 (m, 8H, 4 × CH_2_). HR-ESI-MS: Calcd. for C_45_H_52_N_2_O_5_ [M + H]^+^: 701.3910, found: 701.3947.

5-hydroxy-7–(3-(4-phenylpiperidin-1-yl)propoxy)-2–(4-(3–(4-phenylpiperidin-1-yl)propoxy)phenyl)-4H-chromen-4-one (**10d**). Compound **9a** was treated with 4-phenylpiperidine **4g** based on the above procedure to afford target compound **10d**, light yellow oily matter, 50.8% yield. ^1^H NMR (400 MHz, CDCl_3_) δ 12.80 (s, 1H, OH), 7.83 (d, *J* = 8.7 Hz, 2H, 2 × Ar-H), 7.33 − 7.28 (m, 4H, 2 × Ar-H), 7.22 (dd, *J* = 17.9, 7.5 Hz, 6H, 2 × Ar-H), 7.00 (d, *J* = 8.7 Hz, 2H, 2 × Ar-H), 6.57 (s, 1H, Ar-H), 6.51 (d, *J* = 1.6 Hz, 1H, C = CH), 6.36 (d, *J* = 1.6 Hz, 1H, Ar-H), 4.13 (d, *J* = 1.8 Hz, 4H, 2 × OCH_2_), 3.16 (d, *J* = 6.1 Hz, 4H, 2 × NCH_2_), 2.67–2.64 (m, 4H, 2 × NCH_2_), 2.56 (dd, *J* = 14.9, 7.0 Hz, 2H, 2 × CH), 2.17–2.12 (m, 8H, 2 × NCH_2_ + 2 × CH_2_), 1.91–1.87 (m, 8H, 4 × CH_2_). HR-ESI-MS: Calcd. for C_43_H_48_N_2_O_5_ [M + H]^+^: 673.3597, found: 673.3618.

5-hydroxy-7–(3-(4–(3-phenylpropyl)piperidin-1-yl)propoxy)-2–(4-(3–(4–(3-phenylpropyl)piperidin-1-yl)propoxy)phenyl)-4H-chromen-4-one (**10e**). Compound **9a** was treated with 4-(3-phenylpropyl)piperidine **4i** based on the above procedure to afford target compound **10e**, light yellow oily matter, 45.7% yield. ^1^H NMR (400 MHz, CDCl_3_) δ 12.75 (s, 1H, OH), 7.76 (d, *J* = 8.3 Hz, 2H, 2 × Ar-H), 7.23 (d, *J* = 7.0 Hz, 5H, 5 × Ar-H), 7.13 (d, *J* = 7.6 Hz, 5H, 5 × Ar-H), 6.94 (d, *J* = 8.4 Hz, 2H, 2 × Ar-H), 6.51 (s, 1H, Ar-H), 6.42 (s, 1H, C = CH), 6.27 (s, 1H, Ar-H), 4.06 (d, *J* = 5.2 Hz, 4H, 2 × OCH_2_), 3.20 (s, 4H, 2 × NCH_2_), 2.78 (s, 4H, 2 × NCH_2_), 2.56 (t, *J* = 7.5 Hz, 4H, 2 × NCH_2_), 2.32–2.15 (m, 8H, 4 × CH_2_), 1.76 (d, *J* = 13.5 Hz, 4H, 2 × CH_2_), 1.62–1.57 (m, 10H, 2 × CH + 4 × CH_2_), 1.30 (d, *J* = 6.0 Hz, 4H, 2 × CH_2_). ^13 ^C NMR (100 MHz, CDCl_3_) δ 182.4, 164.5, 164.0, 162.1, 161.7, 157.7, 142.4 (2 C), 128.4 (4 C), 128.4 (4 C), 128.1 (2 C), 125.8 (2 C), 123.6, 115.0 (2 C), 105.7, 104.3, 98.6, 92.9, 66.5, 66.2, 55.1, 55.0, 53.6 (2 C), 53.6 (2 C), 36.1 (2 C), 35.6, 35.6, 30.7, 30.6, 29.8, 29.7, 28.6 (4 C), 25.4, 25.4. HR-ESI-MS: Calcd. for C_49_H_60_N_2_O_5_ [M + H]^+^: 757.4536, found: 757.4571.

7–(3-(3,5-dimethylpiperidin-1-yl)propoxy)-2–(4-(3–(3,5-dimethylpiperidin-1-yl)propoxy)phenyl)-5-hydroxy-4H-chromen-4-one (**10f**). Compound **9a** was treated with 3,5-dimethylpiperidine **4j** based on the above procedure to afford target compound **10f**, light yellow oily matter, 43.4% yield. ^1^H NMR (400 MHz, CDCl_3_) δ 12.78 (s, 1H, OH), 7.80 (d, *J* = 8.6 Hz, 2H, 2 × Ar-H), 6.99 (d, *J* = 8.7 Hz, 2H, 2 × Ar-H), 6.55 (s, 1H, Ar-H), 6.47 (d, *J* = 1.7 Hz, 1H, C = CH), 6.33 (s, 1H, Ar-H), 4.08 (t, *J* = 5.3 Hz, 4H, 2 × OCH_2_), 3.01 − 2.85 (m, 4H, 2 × NCH_2_), 2.57–2.54 (m, 4H, 2 × NCH_2_), 2.07 (dd, *J* = 11.4, 8.0 Hz, 4H, 2 × NCH_2_), 1.73 (d, *J* = 12.3 Hz, 4H, 2 × CH_2_), 1.60–1.48 (m, 4H, 2 × CH_2_), 1.30–1.20 (m, 4H, 4 × CH), 0.87 (d, *J* = 6.2 Hz, 12H, 4 × CH_3_). ^13 ^C NMR (100 MHz, CDCl_3_) δ 182.5, 164.9, 164.1, 162.2, 162.1, 157.8, 128.1 (2 C), 123.6, 115.1 (2 C), 105.6, 104.4, 98.7, 93.1, 67.1, 66.7, 61.5 (2 C), 61.4 (2 C), 55.4, 55.2, 42.0, 42.0, 30.9 (2 C), 30.8 (2 C), 26.4, 26.4, 19.7 (4 C). HR-ESI-MS: Calcd. for C_35_H_48_N_2_O_5_ [M + H]^+^: 577.3597, found: 577.3622.

7–(4-(3,4-dihydroisoquinolin-2(1*H*)-yl)butoxy)-2–(4-(4–(3,4-dihydroisoquinolin-2(1H)-yl)butoxy)phenyl)-5-hydroxy-4*H*-chromen-4-one **(10g).** Compound **9b** was treated with 1,2,3,4-tetrahydroisoquinoline **4b** based on the above procedure to afford target compound **10g**, light yellow oil, 78.2% yield, 98.0% HPLC purity. ^1^H NMR (400 MHz, CDCl_3_) *δ* 12.86 (s, 1H, OH), 7.79 (d, *J* = 8.4 Hz, 2H, 2 × Ar-H), 7.17–7.10 (m, 8H, 8 × Ar-H), 6.96 (d, *J* = 8.0 Hz, 2H, 2 × Ar-H), 6.56 (s, 1H, Ar-H), 6.44 (s, 1H, Ar-H), 6.32 (s, 1H, Ar-H), 4.08–4.02 (m, 4H, 2 × OCH_2_), 3.81 (s, 2H, phCH_2_), 3.77 (s, 2H, phCH_2_), 2.97–2.95 (m, 4H, 2 × phCH_2_), 2.93–2.86 (m, 4H, 2 × NCH_2_), 2.72–2.67 (m, 4H, 2 × NCH_2_), 1.87–1.83 (m, 8H, 4 × CH_2_). HR-ESI-MS: Calcd. for C_41_H_44_N_2_O_5_ [M + H]^+^: 645.3284, found: 645.3306.

7–(4-(benzyl(ethyl)amino)butoxy)-2–(4-(4-(benzyl(ethyl)amino)butoxy)phenyl)-5-hydroxy-4H-chromen-4-one **(10h).** Compound **9b** was treated with *N*-ethylbenzylamine **4c** based on the above procedure to afford target compound **10h**, light yellow oil, 83.7% yield, 99.5% HPLC purity. ^1^H NMR (400 MHz, CDCl_3_) *δ* 12.80 (s, 1H, OH), 7.79 (d, *J* = 8.4 Hz, 2H, 2 × Ar-H), 7.35 (d, *J* = 7.2 Hz, 4H, 4 × Ar-H), 7.30 (t, *J* = 7.6 Hz, 4H, 4 × Ar-H), 7.23 (t, *J* = 7.6 Hz, 2H, 2 × Ar-H), 6.94 (d, *J* = 8.4 Hz, 2H, 2 × Ar-H), 6.54 (s, 1H, Ar-H), 6.42 (s, 1H, Ar-H), 6.30 (s, 1H, Ar-H), 3.99–3.95 (m, 4H, 2 × OCH_2_), 3.61 (s, 4H, 2 × phCH_2_), 2.59–2.50 (m, 8H, 4 × NCH_2_), 1.84–1.80 (m, 4H, 2 × CH_2_), 1.70–1.64 (m, 4H, 2 × CH_2_), 1.08 (t, *J* = 7.2 Hz, 6H, 2 × CH_3_). ^13 ^C NMR (100 MHz, CDCl_3_) *δ* 182.4, 164.9, 164.0, 162.1, 157.7, 139.3, 129.0 (5 C), 128.2 (5 C), 128.0 (2 C), 127.0 (2 C), 123.3, 115.0 (2 C), 105.4, 98.5, 93.0 (2 C), 68.4, 68.0, 58.0 (2 C), 52.5, 52.4, 47.3 (2 C), 26.9, 26.7, 23.4, 23.3, 11.6 (2 C). HR-ESI-MS: Calcd. for C_41_H_48_N_2_O_5_ [M + H]^+^: 649.3597, found: 649.3612.

7–(4-(4-Benzylpiperazin-1-yl)butoxy)-2–(4–(4-(4-benzylpiperazin-1-yl)butoxy)phenyl)-5-hydroxy-4*H*-chromen-4-one **(10i)**. Compound **9b** was treated with benzylpiperazine **4e** based on the above procedure to afford target compound **10i**, light yellow oil, 65.2% yield, 98.1% HPLC purity. ^1^H NMR (400 MHz, CDCl_3_) *δ* 12.80 (s, 1H, OH), 7.80 (d, *J* = 8.8 Hz, 2H, 2 × Ar-H), 7.31–7.24 (m, 10H, 10 × Ar-H), 6.97 (d, *J* = 8.8 Hz, 2H, 2 × Ar-H), 6.52 (s, 1H, Ar-H), 6.45 (d, *J* = 2.0 Hz, 1H, Ar-H), 6.33 (d, *J* = 2.4 Hz, 1H, Ar-H), 4.06–4.01 (m, 4H, 2 × OCH_2_), 3.51 (s, 4H, 2 × phCH_2_), 2.53–3.40 (m, 20 H, 10 × NCH_2_), 1.86–1.80 (m, 4H, 2 × CH_2_), 1.73–1.64 (m, 4H, 2 × CH_2_). HR-ESI-MS: Calcd. for C_45_H_54_N_4_O_5_ [M + H]^+^: 731.4128, found: 731.4143.

7–(4-(4-Benzylpiperidin-1-yl)butoxy)-2–(4–(4-(4-benzylpiperidin-1-yl)butoxy)phenyl)-5-hydroxy-4*H*-chromen-4-one **(10j).** Compound **9b** was treated with benzylpiperidine **4f** based on the above procedure to afford target compound **10j**, light yellow oil, 71.8% yield, 98.2% HPLC purity. ^1^H NMR (400 MHz, CDCl_3_) *δ* 12.79 (s, 1H, OH), 7.80 (d, *J* = 8.4 Hz, 2H, 2 × Ar-H), 7.29–7.25 (m, 4H, 4 × Ar-H), 7.20–7.12 (m, 6H, 6 × Ar-H), 6.97 (d, *J* = 8.4 Hz, 2H, 2 × Ar-H), 6.55 (s, 1H, Ar-H), 6.45 (s, 1H, Ar-H), 6.32 (s, 1H, Ar-H), 4.06–4.02 (m, 4H, 2 × OCH_2_), 2.98 (d, *J* = 10.8 Hz, 4H, 2 × phCH_2_), 2.54 (d, *J* = 6.8 Hz, 4H, 2 × NCH_2_), 2.43 (t, *J* = 7.2 Hz, 4H, 2 × NCH_2_), 1.95 (t, *J* = 11.2 Hz, 4H, 2 × NCH_2_), 1.84–1.80 (m, 4H, 2 × CH_2_), 1.72–1.64 (m, 8H, 4 × CH_2_), 1.57–1.53 (m, 2H, 2 × CH), 1.42–1.31 (m, 4H, 2 × CH_2_). HR-ESI-MS: Calcd. for C_47_H_56_N_2_O_5_ [M + H]^+^: 729.4223, found: 729.4267.

7-((5-(benzyl(ethyl)amino)pentyl)oxy)-2–(4-((5-(benzyl(ethyl)amino)pentyl)oxy)phenyl)-5-hydroxy-4H-chromen-4-one (**10k**). Compound **9c** was treated with *N*-ethylbenzylamine **4c** based on the above procedure to afford target compound **10k**, light yellow oily matter, 49.3% yield. ^1^H NMR (400 MHz, CDCl_3_) δ 12.81 (s, 1H, OH), 7.82 (d, *J* = 8.6 Hz, 2H, 2 × Ar-H), 7.32 (q, *J* = 7.9 Hz, 7H, 7 × Ar-H), 7.25 (d, *J* = 10.6 Hz, 3H, 3 × Ar-H), 6.98 (d, *J* = 8.6 Hz, 2H, 2 × Ar-H), 6.57 (s, 1H, Ar-H), 6.46 (s, 1H, C = CH), 6.34 (s, 1H, Ar-H), 4.00 (dd, *J* = 10.1, 6.0 Hz, 4H, 2 × OCH_2_), 3.57 (s, 4H, 2 × NCH_2_), 2.56 − 2.42 (m, 8H, 4 × NCH_2_), 1.79 (s, 4H, 2 × CH_2_), 1.60–1.51 (m, 4H, 2 × CH_2_), 1.47 (d, *J* = 5.9 Hz, 4H, 2 × CH_2_), 1.05 (t, *J* = 7.0 Hz, 6H, 2 × CH_3_). ^13 ^C NMR (100 MHz, CDCl_3_) δ 182.6, 165.1, 164.2, 162.3, 162.2, 157.8, 129.0 (4 C), 128.3 (6 C), 128.1 (2 C), 126.9 (2 C), 123.5, 115.1 (2 C), 105.6, 104.3, 98.6, 93.1, 68.7, 68.4, 58.2 (2 C), 53.1 (2 C), 47.5 (2 C), 29.1, 29.0, 26.9, 26.9, 24.0, 23.9, 11.8 (2 C). HR-ESI-MS: Calcd. for C_43_H_52_N_2_O_5_ [M + H]^+^: 677.3910, found: 677.3943.

7-((5-(ethyl(2-methoxybenzyl)amino)pentyl)oxy)-2–(4-((5-(ethyl(2-methoxybenzyl)amino)pentyl)oxy)phenyl)-5-hydroxy-4H-chromen-4-one (**10l**). Compound **9c** was treated with *N*-(2-methoxybenzyl)ethanamine **4d** based on the above procedure to afford target compound **10l**, light yellow oily matter, 38.1% yield. ^1^H NMR (400 MHz, CDCl_3_) δ 12.82 (s, 1H, OH), 7.83 (d, *J* = 8.0 Hz, 2H, 2 × Ar-H), 7.50 (d, *J* = 7.0 Hz, 2H, 2 × Ar-H), 7.28 (s, 2H, 2 × Ar-H), 6.98 (t, *J* = 8.8 Hz, 4H, 4 × Ar-H), 6.89 (d, *J* = 8.0 Hz, 2H, 2 × Ar-H), 6.57 (s, 1H, Ar-H), 6.47 (s, 1H, C = CH,), 6.34 (s, 1H, Ar-H), 4.03 (s, 4H, 2 × OCH_2_), 3.85 (s, 6H, 2 × OCH_3_), 3.80 (d, *J* = 8.8 Hz, 4H, 2 × NCH_2_), 2.78 − 2.55 (m, 8H, 4 × NCH_2_), 1.83 (s, 4H, 2 × CH_2_), 1.71 (s, 4H, 2 × CH_2_), 1.50 (d, *J* = 5.8 Hz, 4H, 2 × CH_2_), 1.18 (t, *J* = 6.2 Hz, 6H, 2 × CH_3_). ^13 ^C NMR (100 MHz, CDCl_3_) δ 182.5, 165.0, 164.1, 162.2, 162.1, 157.9 (2 C), 157.8, 131.0, 131.0 (2 C), 128.9, 128.8, 128.7, 128.1 (2 C), 123.4, 120.6 (2 C), 115.0 (2 C), 110.5 (2 C), 105.5, 104.3, 98.6, 93.0, 68.5, 68.2, 65.6, 55.5 (2 C), 53.0, 51.1 (2 C), 47.6 (2 C), 30.6, 29.0, 28.8, 23.9, 23.9, 19.3, 13.8, 11.2. HR-ESI-MS: Calcd. for C_45_H_56_N_2_O_7_ [M + H]^+^: 737.4121, found: 737.4143.

7-((5–(4-benzylpiperidin-1-yl)pentyl)oxy)-2–(4-((5–(4-benzylpiperidin-1-yl)pentyl)oxy)phenyl)-5-hydroxy-4H-chromen-4-one (**10m**). Compound **9c** was treated with benzylpiperidine **4f** based on the above procedure to afford target compound **10m**, light yellow oily matter, 45.2% yield. ^1^H NMR (400 MHz, CDCl_3_) δ 12.76 (s, 1H, OH), 7.77 (d, *J* = 8.6 Hz, 2H, 2 × Ar-H), 7.23 (d, *J* = 4.1 Hz, 4H, 4 × Ar-H), 7.17 (d, *J* = 7.1 Hz, 2H, 2 × Ar-H), 7.10 (d, *J* = 7.3 Hz, 4H, 4 × Ar-H), 6.94 (d, *J* = 8.6 Hz, 2H, 2 × Ar-H), 6.52 (s, 1H, Ar-H), 6.42 (s, 1H, C = CH), 6.28 (s, 1H, Ar-H), 3.99 (d, *J* = 2.3 Hz, 4H, 2 × OCH_2_), 3.12 (d, *J* = 10.8 Hz, 4H, 2 × NCH_2_), 2.53 (s, 8H, 4 × NCH_2_), 2.11 (s, 4H, 2 × CH_2_), 1.81 (s, 4H, 2 × CH_2_), 1.68 (s, 8H, 4 × CH_2_), 1.56 (s, 6H, 2 × CH, 2 × CH_2_), 1.47 (d, *J* = 5.8 Hz, 4H, 2 × CH_2_). ^13 ^C NMR (100 MHz, CDCl_3_) δ 182.5, 164.9, 164.1, 162.1 (2 C), 157.8, 140.2 (2 C), 129.2 (4 C), 128.4 (4 C), 128.1 (2 C), 126.1 (2 C), 123.4, 115.0 (2 C), 105.6, 104.3, 98.6, 93.0, 68.3, 68.0, 58.3, 58.3, 53.6 (4 C), 42.7 (2 C), 37.4 (2 C), 30.8 (4 C), 28.9, 28.8, 25.6, 25.6, 24.0, 23.9. HR-ESI-MS: Calcd. for C_49_H_60_N_2_O_5_ [M + H]^+^: 757.4536, found: 757.4559.

5-hydroxy-7-((5–(4-phenylpiperidin-1-yl)pentyl)oxy)-2–(4-((5–(4-phenylpiperidin-1-yl)pentyl)oxy)phenyl)-4H-chromen-4-one (**10n**). Compound **9c** was treated with 4-phenylpiperidine **4g** based on the above procedure to afford target compound **10n**, light yellow oily matter, 80.2% yield. ^1^H NMR (400 MHz, CDCl_3_) δ 12.83 (s, 1H, OH), 7.83 (d, *J* = 8.6 Hz, 2H, 2 × Ar-H), 7.31 (dd, *J* = 14.1, 6.8 Hz, 5H, 5 × Ar-H), 7.25 − 7.14 (m, 5H, 5 × Ar-H), 7.00 (d, *J* = 8.6 Hz, 2H, 2 × Ar-H), 6.57 (s, 1H, Ar-H), 6.48 (s, 1H, C = CH), 6.35 (s, 1H, Ar-H), 4.05 (s, 4H, 2 × OCH_2_), 3.14 (d, *J* = 11.1 Hz, 4H, 2 × NCH_2_), 2.55 (dd, *J* = 15.0, 6.9 Hz, 2H, 2 × CH), 2.51 − 2.41 (m, 4H, 2 × NCH_2_), 2.13 (t, *J* = 12.7 Hz, 4H, 2 × NCH_2_), 1.88 (s, 12H, 6 × CH_2_), 1.67 (d, *J* = 6.1 Hz, 4H, 2 × CH_2_), 1.54 (d, *J* = 6.2 Hz, 4H, 2 × CH_2_). ^13 ^C NMR (100 MHz, CDCl_3_) δ 182.4, 165.0, 164.1, 162.2, 162.1, 157.7, 146.1, 146.1, 128.5 (4 C), 128.1 (2 C), 126.9 (4 C), 126.3 (2 C), 123.4, 115.0 (2 C), 105.5, 104.2, 98.5, 93.0, 68.5, 68.1, 58.9, 58.9, 54.4 (4 C), 42.6 (2 C), 33.2 (4 C), 29.1, 28.9, 26.6, 26.6, 24.2, 24.1. HR-ESI-MS: Calcd. for C_47_H_56_N_2_O_5_ [M + H]^+^: 729.4223, found: 729.4249.

5-hydroxy-7-((5–(4-(3-phenylpropyl)piperidin-1-yl)pentyl)oxy)-2–(4-((5–(4–(3-phenylpropyl)piperidin-1-yl)pentyl)oxy)phenyl)-4H-chromen-4-one (**10o**). Compound **9c** was treated with 4-(3-phenylpropyl)piperidine **4i** based on the above procedure to afford target compound **10o**, light yellow oily matter, 73.8% yield. ^1^H NMR (400 MHz, CDCl_3_) *δ* 12.80 (s, 1H, OH), 7.82 (d, *J* = 8.5 Hz, 2H, 2 × Ar-H), 7.26 (s, 5H, 5 × Ar-H), 7.17 (t, *J* = 7.2 Hz, 5H, 5 × Ar-H), 6.98 (d, *J* = 8.6 Hz, 2H, 2 × Ar-H), 6.56 (s, 1H, Ar-H), 6.46 (s, 1H, C = CH), 6.33 (s, 1H, Ar-H), 4.02 (s, 4H, 2 × OCH_2_), 3.05 (d, *J* = 10.8 Hz, 4H, 2 × NCH_2_), 2.58 (t, *J* = 7.7 Hz, 4H, 2 × NCH_2_), 2.50–2.39 (m, 4H, 2 × NCH_2_), 2.04 (t, *J* = 10.3 Hz, 4H, 2 × CH_2_), 1.84 (s, 4H, 2 × CH_2_), 1.76–1.55 (m, 12H, 6 × CH_2_), 1.49 (d, *J* = 5.3 Hz, 4H, 2 × CH_2_), 1.32–1.25 (m, 10H, 2 × CH + 4 × CH_2_). ^13 ^C NMR (100 MHz, CDCl_3_) δ 182.6 165.0, 164.2, 162.2 (2 C), 157.8, 142.7 (2 C), 128.5 (4 C), 128.4 (4 C), 128.1 (2 C), 125.8 (2 C), 123.5, 115.1 (2 C), 105.6, 104.4, 98.6, 93.1, 68.4, 68.1, 58.7, 58.7, 53.9 (4 C), 36.2 (4 C), 36.0, 35.5, 31.6, 31.6, 29.1, 28.9, 28.8 (4 C), 26.2, 26.1, 24.1, 24.1. HR-ESI-MS: Calcd. for C_53_H_68_N_2_O_5_ [M + H]^+^: 813.5162, found: 813.5187.

7-((5–(3,5-dimethylpiperidin-1-yl)pentyl)oxy)-2–(4-((5–(3,5-dimethylpiperidin-1-yl)pentyl)oxy)phenyl)-5-hydroxy-4H-chromen-4-one (**10p**). Compound **9c** was treated with 3,5-dimethylpiperidine **4j** based on the above procedure to afford target compound **10p**, light yellow oily matter, 80.2% yield. ^1^H NMR (400 MHz, CDCl_3_) δ 12.80 (s, 1H, OH), 7.82 (d, *J* = 8.4 Hz, 2H, 2 × Ar-H), 6.99 (d, *J* = 8.5 Hz, 2H, 2 × Ar-H), 6.57 (s, 1H, Ar-H), 6.47 (s, 1H, C = CH), 6.34 (s, 1H, Ar-H), 4.03 (s, 4H, 2 × OCH_2_), 2.95 (s, 4H, 2 × NCH_2_), 2.46 (s, 4H, 2 × NCH_2_), 1.85 (s, 8H, 2 × NCH_2_, 2 × CH_2_), 1.68 (s, 12H, 4 × CH, 4 × CH_2_), 1.50 (s, 4H, 2 × CH_2_), 0.87 (d, *J* = 6.5 Hz, 12H, 4 × CH_3_). HR-ESI-MS: Calcd. for C_39_H_56_N_2_O_5_ [M + H]^+^: 633.4223, found: 633.4251.

7-((6–(3,4-dihydroisoquinolin-2(1H)-yl)hexyl)oxy)-2–(4-((6–(3,4-dihydroisoquinolin-2(1H)-yl)hexyl)oxy)phenyl)-5-hydroxy-4H-chromen-4-one (**10q**). Compound **9d** was treated with 1,2,3,4-tetrahydroisoquinoline **4b** based on the above procedure to afford target compound **10q**, light yellow oily matter, 78.9% yield. ^1^H NMR (400 MHz, CDCl_3_) δ 12.81 (s, 1H, OH), 7.82 (d, *J* = 8.7 Hz, 2H, 2 × Ar-H), 7.11 (d, *J* = 4.1 Hz, 6H, 6 × Ar-H), 7.01 (dd, *J* = 12.1, 7.2 Hz, 4H, 4 × Ar-H), 6.57 (s, 1H, Ar-H), 6.47 (d, *J* = 1.8 Hz, 1H, C = CH), 6.35 (d, *J* = 1.8 Hz, 1H, Ar-H), 4.08–4.01 (m, 4H, 2 × OCH_2_), 3.66–3.63 (m, 4H, 2 × NCH_2_), 2.92 (t, *J* = 5.6 Hz, 4H, 2 × NCH_2_), 2.75 (t, *J* = 5.8 Hz, 4H, 2 × CH_2_), 2.60–2.51 (m, 4H, 2 × NCH_2_), 1.87 (d, *J* = 5.7 Hz, 8H, 4 × CH_2_), 1.73–1.69 (m, 4H, 2 × CH_2_), 1.58–1.55 (m, 4H, 2 × CH_2_). ^13 ^C NMR (100 MHz, CDCl_3_) δ 182.6, 165.1, 164.2, 162.3 (2 C), 157.8, 134.8 (2 C), 134.4 (2 C), 128.8 (2 C), 128.1 (2 C), 126.7 (2 C), 126.2 (2 C), 125.7 (2 C), 123.5, 115.1 (2 C), 105.6, 104.4, 98.6, 93.2, 68.6, 68.3, 58.4 (2 C), 56.3 (2 C), 51.1 (2 C), 29.2 (4 C), 29.0 (2 C), 27.0 (2 C), 24.1 (2 C). HR-ESI-MS: Calcd. for C_45_H_52_N_2_O_5_ [M + H]^+^: 701.3910, found: 701.3938.

7-((6-(benzyl(ethyl)amino)hexyl)oxy)-2–(4-((6-(benzyl(ethyl)amino)hexyl)oxy)phenyl)-5-hydroxy-4H-chromen-4-one (**10r**). Compound **9d** was treated with *N*-ethylbenzylamine **4c** based on the above procedure to afford target compound **10r**, light yellow oily matter, 76.2% yield. ^1^H NMR (400 MHz, CDCl_3_) δ 12.74 (s, 1H, OH), 7.74 (d, *J* = 8.2 Hz, 2H, 2 × Ar-H), 7.21 − 7.18 (m, 3H, 3 × Ar-H), 7.13 (dd, *J* = 17.8, 7.1 Hz, 7H, 7 × Ar-H), 6.91 (d, *J* = 8.2 Hz, 2H, 2 × Ar-H), 6.48 (s, 1H, Ar-H), 6.39 (s, 1H, C = CH), 6.26 (s, 1H, Ar-H), 3.97–3.95 (m, 4H, 2 × OCH_2_), 3.06 (d, *J* = 10.6 Hz, 4H, 2 × NCH_2_), 2.40 − 2.30 (m, 4H, 2 × NCH_2_), 2.04 (t, *J* = 9.7 Hz, 4H, 2 × NCH_2_), 1.81–1.77 (m, 10H, 2 × CH_2_ + 2 × CH_3_), 1.58–1.55 (m, 4H, 2 × CH_3_), 1.46–1.42 (m, 4H, 2 × CH_3_), 1.34 (d, *J* = 6.1 Hz, 4H, 2 × CH_3_). ^13 ^C NMR (100 MHz, CDCl_3_) δ 182.5, 165.1, 164.1, 162.3, 162.2, 157.8, 129.2 (4 C), 128.3 (6 C), 128.1 (2 C), 127.1 (2 C), 123.4, 115.0 (2 C), 105.5, 104.3, 98.6, 93.1, 68.6, 68.3, 57.9 (2 C), 52.9 (2 C), 47.3 (2 C), 29.2, 29.0, 27.2, 27.2, 26.7, 26.6, 26.0, 25.9, 11.5 (2 C). HR-ESI-MS: Calcd. for C_45_H_56_N_2_O_5_ [M + H]^+^: 705.4223, found: 705.4250.

7-((6–(4-benzylpiperidin-1-yl)hexyl)oxy)-2–(4-((6–(4-benzylpiperidin-1-yl)hexyl)oxy)phenyl)-5-hydroxy-4H-chromen-4-one (**10s**). Compound **9d** was treated with benzylpiperidine **4f** based on the above procedure to afford target compound **10s**, light yellow oily matter, 83.4% yield. ^1^H NMR (400 MHz, CDCl_3_) δ 12.74 (s, 1H, OH), 7.74 (d, *J* = 8.5 Hz, 2H, 2 × Ar-H), 7.20 (s, 4H, 4 × Ar-H), 7.11 (dd, *J* = 13.5, 6.5 Hz, 2H, 2 × Ar-H), 7.06 (d, *J* = 7.2 Hz, 4H, 4 × Ar-H), 6.91 (d, *J* = 8.4 Hz, 2H, 2 × Ar-H), 6.49 (s, 1H, Ar-H), 6.39 (s, 1H, C = CH), 6.25 (s, 1H, Ar-H), 3.95–3.92 (m, 4H, 2 × OCH_2_), 3.09 (d, *J* = 10.5 Hz, 4H, 2 × NCH_2_), 2.50–2.47 (m, 8H, 4 × NCH_2_), 2.09–2.06 (m, 4H, 2 × CH_2_), 1.76–1.73 (m, 4H, 2 × CH_2_), 1.64–1.62 (m, 8H, 4 × CH_2_), 1.55–1.51 (m, 6H, 2 × CH_2_ + 2 × CH), 1.43–1.41 (m, 4H, 2 × CH_2_), 1.36–1.28 (m, 4H, 2 × CH_2_). ^13 ^C NMR (100 MHz, CDCl_3_) δ 182.5, 165.0, 164.1, 162.2, 162.2 157.8, 140.2 (2 C), 129.2 (4 C), 128.4 (4 C), 128.1 (2 C), 126.1 (2 C), 123.4, 115.0 (2 C), 105.5, 104.3, 98.6, 93.1, 68.5, 68.2, 58.3, 58.3, 53.5 (4 C), 42.8 (2 C), 37.5 (2 C), 30.8 (2 C), 29.8, 29.8, 29.0, 28.9, 27.2, 27.2, 25.9, 25.8, 25.7, 25.6. HR-ESI-MS: Calcd. for C_51_H_64_N_2_O_5_ [M + H]^+^: 785.4849, found: 785.4871.

5-hydroxy-7-((6–(4-phenylpiperidin-1-yl)hexyl)oxy)-2–(4-((6–(4-phenylpiperidin-1-yl)hexyl)oxy)phenyl)-4H-chromen-4-one (**10t**). Compound **9d** was treated with 4-phenylpiperidine **4g** based on the above procedure to afford target compound **10t**, light yellow oily matter, 80.7% yield. ^1^H NMR (400 MHz, CDCl_3_) δ 12.81 (s, 1H, OH), 7.82 (d, *J* = 8.3 Hz, 2H, 2H, 2 × Ar-H), 7.31 (dd, *J* = 20.5, 13.4 Hz, 10H, 10 × Ar-H), 6.98 (d, *J* = 8.3 Hz, 2H, 2 × Ar-H), 6.56 (s, 1H, Ar-H), 6.46 (s, 1H, C = CH), 6.34 (s, 1H, Ar-H), 4.01–3.99 (m, 4H, 2 × OCH_2_), 3.64–3.61 (m, 4H, 2 × NCH_2_), 2.56 (d, *J* = 6.7 Hz, 4H, 2 × NCH_2_), 2.52 − 2.45 (m, 4H, 2 × NCH_2_), 1.82–1.78 (m, 4H, 2 × CH_2_), 1.56–1.54 (m, 4H, 2 × CH_2_), 1.46–1.44 (m, 4H, 2 × CH_2_), 1.37–1.34 (m, 4H, 2 × CH_2_), 1.26–1.24 (m, 4H, 2 × CH_2_), 1.08 (t, *J* = 6.7 Hz, 6H, 2 × CH, 2 × CH_2_). ^13 ^C NMR (100 MHz, CDCl_3_) δ 182.5, 165.0, 164.1, 162.2, 162.1, 157.7, 146.0 (2 C), 128.5 (4 C), 128.0 (2 C), 126.9 (4 C), 126.3 (2 C), 123.3, 115.0 (2 C), 105.5, 104.2, 98.5, 93.0, 68.6, 68.2, 58.9 (2 C), 54.3 (4 C), 42.6 (2 C), 33.1 (4 C), 29.1, 28.9, 27.4, 27.4, 26.7, 26.6, 26.0, 25.9. HR-ESI-MS: Calcd. for C_49_H_60_N_2_O_5_ [M + H]^+^: 757.4536, found: 757.4555.

5-hydroxy-7-((6–(4-(3-phenylpropyl)piperidin-1-yl)hexyl)oxy)-2–(4-((6–(4–(3-phenylpropyl)piperidin-1-yl)hexyl)oxy)phenyl)-4H-chromen-4-one (**10u**). Compound **9d** was treated with 4-(3-phenylpropyl)piperidine **4i** based on the above procedure to afford target compound **10u**, light yellow oily matter, 60.6% yield. ^1^H NMR (400 MHz, CDCl_3_) δ 12.81 (s, 1H, OH), 7.82 (d, *J* = 8.4 Hz, 2H, 2 × Ar-H), 7.27 (s, 3H, 3 × Ar-H), 7.17 (d, *J* = 7.2 Hz, 7H, 7 × Ar-H), 6.99 (d, *J* = 8.4 Hz, 2H, 2 × Ar-H), 6.56 (s, 1H, Ar-H), 6.46 (s, 1H, C = CH), 6.33 (s, 1H, Ar-H), 4.03–4.01 (m, 4H, 2 × OCH_2_), 3.05–3.04 (m, 4H, 2 × NCH_2_), 2.59 (t, *J* = 7.4 Hz, 4H, 2 × phCH_2_), 2.46–2.35 (m, 4H, 2 × NCH_2_), 2.00 (t, *J* = 10.4 Hz, 4H, 2 × NCH_2_), 1.84–1.82 (m, 4H, 2 × CH_2_), 1.71 (d, *J* = 11.5 Hz, 4H, 2 × CH_2_), 1.63–1.60 (m, 8H, 4 × CH_2_), 1.51–1.47 (m, 4H, 2 × CH_2_), 1.41–1.36 (m, 6H, 2 × CH_2_, 2 × CH), 1.332–1.28 (m, 8H, 4 × CH_2_). ^13 ^C NMR (100 MHz, CDCl_3_) δ 182.5, 165.0, 164.1, 162.2, 162.1, 157.7, 142.7 (2 C), 128.4 (4 C), 128.3 (4 C), 128.1 (2 C), 125.7 (2 C), 123.4, 115.0 (2 C), 105.5, 104.2, 98.5, 93.1, 68.5, 68.2, 58.8 (2 C), 53.9 (4 C), 36.2 (2 C), 36.1, 35.5, 31.7 (4 C), 29.1, 28.9, 28.8 (4 C), 27.4, 27.3, 26.4, 26.4, 26.0, 25.9. HR-ESI-MS: Calcd. for C_55_H_72_N_2_O_5_ [M + H]^+^: 841.5475, found: 841.5498.

7-((6–(3,5-dimethylpiperidin-1-yl)hexyl)oxy)-2–(4-((6–(3,5-dimethylpiperidin-1-yl)hexyl)oxy)phenyl)-5-hydroxy-4H-chromen-4-one (**10v**). Compound **9d** was treated with 3,5-dimethylpiperidine **4j** based on the above procedure to afford target compound **10v**, light yellow oily matter, 67.9% yield. ^1^H NMR (400 MHz, CDCl_3_) δ 12.79 (s, 1H, OH), 7.80 (d, *J* = 8.2 Hz, 2H, 2 × Ar-H), 6.97 (d, *J* = 8.2 Hz, 2H, 2 × Ar-H), 6.55 (s, 1H, Ar-H), 6.45 (s, 1H, C = CH), 6.32 (s, 1H, Ar-H), 4.01–3.99 (m, 4H, 2 × OCH_2_), 2.99 (d, *J* = 10.3 Hz, 4H, 2 × NCH_2_), 2.50 − 2.37 (m, 4H, 2 × NCH_2_), 1.80–1.77 (m, 8H, 2 × NCH_2_, 2 × CH_2_), 1.63–1.57 (m, 8H, 4 × CH, 2 × CH_2_), 1.50–1.46 (m, 4H, 2 × CH_2_), 1.38–1.34 (m, 4H, 2 × CH_2_), 1.25–1.21 (m, 4H, 2 × CH_2_), 0.86 (d, *J* = 6.2 Hz, 12H, 4 × CH_3_). ^13 ^C NMR (100 MHz, CDCl_3_) δ 182.5, 165.0, 164.1, 162.2, 162.1, 157.8, 128.1 (2 C), 123.4, 115.0 (2 C), 105.5, 104.2, 98.6, 93.1, 68.5, 68.2, 60.8 (4 C), 58.6 (2 C), 41.6 (2 C), 30.3 (4 C), 29.8, 29.0, 28.9, 27.3, 27.3, 25.9 (2 C), 25.9, 19.5 (4 C). HR-ESI-MS: Calcd. for C_41_H_60_N_2_O_5_ [M + H]^+^: 661.4536, found: 661.4572.

### 4.2. Biological activity

#### 4.2.1. Antioxidant activity

The antioxidant potency of target compounds was tested using an oxygen radical absorbance capacity fluorescein (ORAC-FL) assay. The detailed procedure was reported in our previous work^17^^,^^22^.

#### 4.2.2. Inhibition experiments of AChE and BuChE

The AChE and BuChE inhibitory activities of the synthesised compounds were evaluated using the Ellman assay with slight modification. AChE from 5% rat cortex homogenate or human erythrocytes (Sigma Co.). BuChE from rat serum or human serum (Sigma Co.). The detailed procedure referenced our previous work^17^^,^^20^.

#### 4.2.3. Inhibitory effect on self-induced/Cu^2+^-induced Aβ_1-42_ aggregation

The Thioflavin T-based flurometric assay was employed to test the inhibition potency on self-induced*/Cu^2+^-induced* A*β*_1-42_ aggregation. The detailed procedure referenced our previous work^20^^,^^22^^,^^28^.

#### 4.2.4. Metal chelation property

The metal chelation property was investigated by UV absorption in a Shimadzu UV-2450 spectrophotometer. The detailed procedure referenced our previous work^20^^,^^22^.

#### 4.2.5. Transmission electron microscopy (TEM) assay

For the Cu^2+^-induced experiment, the Aβ stock solution was diluted with HEPES buffer (20 mM, pH 6.6, 150 mM NaCl). The sample preparation was the same as for the ThT assay. Aliquots (10 μL) of the samples were placed on a carbon-coated copper/rhodium grid for 2 min at room temperature. The excess sample was removed using filter paper followed by washing twice with ddH_2_O. Each grid was incubated with uranyl acetate (1% w/v ddH_2_O). Upon removal of excess uranyl acetate, the grids were dried for 15 min at room temperature. Images from each sample were taken on a transmission electron microscope^20^^,^^22^.

#### 4.2.6. Neuroprotective effects on H_2_O_2_-induced PC12 cell injury

The neuroprotective effects of **5f** and **7k** were evaluated on H_2_O_2_-induced PC12 cell injury. The cell viability was measured with MTT assay and the detailed procedure referenced our previous work^20^^,^^22^.

#### 4.2.7. *In vitro* anti-neuroinflammatory activity evaluation

The anti-neuroinflammatory property of compounds **5f** and **7k** was assessed using BV-2 cells by evaluating the level of NO production and TNF-α production^32^^,^^33^. Firstly, the cytotoxicity of compounds **5f** and **7k** was tested on BV-2 cells using an MTT assay. BV-2 cells were seeded in DMEM (100 μL) and cultured in a 96-well cell culture microplate and incubated in a humidified incubator containing 5% CO_2_ at 37 °C for 24 h. Then, 10 μL of **5f** or **7k** were added to cells in triplicate wells, followed by incubation for another 30 min. Subsequently, the treated cells were exposed to LPS (1.0 μg/mL, 10 μ L) and continued the culture for 24 h, and MTT solution was added to each well and incubated for 4 h at 37 °C. Finally, the crystals obtained were dissolved in DMSO (200 μ L) and OD values were measured at 490 nm. The results were recorded and expressed as the percent cell viability compared to the control group.

Griess reagent system was used to evaluate the inhibition of LPS-induced NO production. The methods of culturing BV-2 cells, setting blank control and adding tested compounds **5f** or **7k** (1 μM, 3 μM and 9 μM) and LPS were the same as those in the cytotoxicity assay. After that, the cell culture supernatants and different concentrations of NaNO_2_ as a standard were added to a 96-well plate since the NaNO_2_ concentration in the supernatant could act as an indicator of NO production. Then, to each well was added the same volume of Griess reagent and incubated at room temperature for 10 min. Finally, the absorbance was monitored at 540 nm using an ELISA plate reader to measure the results of NO production.

Inhibition of LPS-induced TNF-α production was evaluated with an enzyme-linked immunosorbent assay (ELISA). The methods of BV-2 cells culture, adding different concentrations of tested compounds **5f** or **7k** (1 μM, 3 μM and 9 μM) (10 μL) and LPS (1.0 μg/mL, 10 μL) and setting blank control group were the same as above in cytotoxicity assay. Then, the supernatant of the cell culture solution (50 μL) was added to a 96-well plate and the release of cytokine TNF-α was measured by ELISA according to the instructions of the ELISA kit.

#### 4.2.8. Hepatoprotective activity^20^^,^^22^

1. *Materials.* LO2 cells purchased from Wuhan Punosi Life Technology Co., Ltd. 30% H_2_O_2_, Trichloroacetic acid (TCA), SRB, DMEM medium (HyClone), Foetal bovine serum (LNSER, article number: S711-001S), Penicillin streptomycin mixture (double antibiotic) (Solarbio), 0.25% trypsin-EDTA digestive juice (Solarbio), DMSO (Solarbio), Potassium dihydrogen phosphate (Xiyu Science Co., Ltd., CAS: 7778-77-0); Ultra-clean workbench (Wuxi Easy Purification Equipment Co., Ltd., model: SW-CJ-VS2); Carbon dioxide incubator (Esco, model: 2014-88759); Full-wavelength microplate reader (ThermoFisher, USA, model: 1510-01871);
2. *Experiment method.* (a) Cell culture: cultured in DMEM medium containing 10% FBS and 1% double-antibody, placed in 37 °C, 5% CO_2_ constant temperature and humidity incubator, changed every 2 days, subcultured when cells were 90% confluent. (b) Effect of compounds on LO2 proliferation by SRB assay: Discard the culture medium, fix the cells with 10% (m/v) trichloroacetic acid (TCA) 100 μL, and place in a refrigerator at 4 °C for 1 h. The fixing solution was discarded, washed 5 times with distilled water, and dried in an oven. Adding 100 μL SRB solution to each well after drying, and leave it at room temperature for 10-30 min. Discard the supernatant, wash it 5 times with 1% glacial acetic acid water, air dry. Finally, add 100 μL/well of 10 mmol/L Tris-based buffer solution and oscillate on a plate shaker. The OD value was measured at 515 nm using a microplate reader. Cell viability (%) = OD test well/OD blank control well x 100%. (c) Effect of compounds on H_2_O_2_-induced LO2 cell injury by SRB assay. The LO2 cells in the logarithmic growth phase were passaged, and the single-cell suspension with a cell density of 4 × 10^5^/mL was adjusted, and the cells were seeded on a 96-well plate at 100 μL per well, and a blank control group, H_2_O_2_ was set. Group, 10 micromolar compounds group, each group of 3 duplicate wells, after 24h of culture, the old culture solution was discarded, the blank well and H_2_O_2_ group were replaced with complete medium, and the monomer compound group was replaced with a drug containing 10 micromolar. Complete medium. After 48h of culture, the H_2_O_2_ group and the monomer compound group were added with hydrogen peroxide prepared in a complete medium, and the final concentration of hydrogen peroxide was 1 mmol. The blank group and the H_2_O_2_ group were added with an equal volume of complete medium, and the culture was continued for 6h. After treatment with H_2_O_2_ for 6h, the survival rate was measured by the SRB method.

#### 4.2.9. *In vivo* assay

##### 4.2.9.1. Acute toxicity

Kunming mice at a body weight of 18**–**22 g (six weeks old, either gender) were supplied by the Centre of Experimental Animals of Sichuan Academy of Chinese Medicine Sciences (eligibility certification no. SCXK-Chuan2018-19). Mice were maintained under standard conditions with a 12 h:12 h light**-**dark cycle at 20−22 °C with a relative humidity of 60 − 70%. Sterile food and water were provided according to institutional guidelines. Prior to each experiment, mice were fasted overnight and allowed free access to water. Compound **5f** at doses of 1000, 500, 250 and 100 mg/kg (*n* = 6 per group) by intragastric administration. After the administration of the compound **5f**, the mice were observed continuously for the first 4 h for any abnormal behaviour and mortality changes, intermittently for the next 24 h, and occasionally thereafter for 14 days for the onset of any delayed effects. All animals were sacrificed on the 14th day after drug administration and were macroscopically examined for possible damage to the heart, liver, and kidneys.

#### 4.2.10. Assay method

The step-down passive avoidance task was employed to investigate the effects of **5f** on scopolamine-induced memory impairment^17^^,^^20^. Sixty mice were randomly divided into six groups. They were compound **5f** group (1.9 mg/kg, 5.7 mg/kg and 17.1 mg/kg), same volume of water (untreated group), model group (3 mg/kg scopolamine), 5 mg/kg donepezil. After 30 min, memory impairment was induced by administering scopolamine (3 mg/kg). Then 30 min later, the learning and memory capacity of the mouse were measured by the Y-maze test. The maze was made of black-colored acryl and positioned at equal angles. Rats were placed at the end of the arm and allowed to move freely through the maze during 8 min sessions. Arm entry sessions were recorded when the hind paws of the rat were completely placed in the arm. Consecutive entry into three arms in alternative order was defined as successive entries on overlapping triplet sets and the alternation percentage was calculated as the ratio of actual to possible alternations (defined as the total number of arm entries minus 2), multiplied by 100.

The representative ^1^H and ^13 ^C NMR spectra for the synthesised compounds are available as Supplementary material.

## Supplementary Material

Supplemental Material
