# Supplementary material for: Development of naringenin-O-alkylamine derivatives as multifunctional agents for the treatment of Alzheimer’s disease
Source: J Enzyme Inhib Med Chem. 2022 Feb 22;37(1):792–816. doi: 10.1080/14756366.2022.2041627 (PMC8881077; doi:10.1080/14756366.2022.2041627)
Supplement: Supplemental Material [file IENZ_A_2041627_SM5200.pdf]

Supplementary material

Development of naringenin-*O*-alkylamine derivatives as multifunctional agents for the treatment of Alzheimer's disease

Jing Yang[#,1], Yi Zhou[#,1], Yujuan Ban[#, 2], Jing Mi[1], Ying He[1], Xinjuan Li[1], Zhengwei Liu[1], Keren Wang[1], Gaofeng Zhu[2], Wenmin Liu[1], Zhenghuai Tan[*,3], Zhipei Sang[*,1,2].

[1]College of Chemistry and Pharmaceutical Engineering, Nanyang Normal University, Nanyang, 473061, China

[2]State Key Laboratory of Functions and Applications of Medicinal Plants, Guizhou Provincial Engineering Technology Research Center for Chemical Drug R&D, Guizhou Medical University, Guiyang, 550004, China

[3]Institute of Traditional Chinese Medicine Pharmacology and Toxicology, Sichuan Academy of Chinese Medicine Sciences, Chengdu, 610041, China

**Corresponding Author*.

E-mail: sangzhipei@126.com (Zhipei Sang)

E-mail: tanzhh616@163.com (Zhenghuai Tan)

#These authors contributed equally.

Contents

A. Representative ^{1}H, ^{13}C NMR, HR-ESI-MS and HPLC spectra for the synthesized compounds

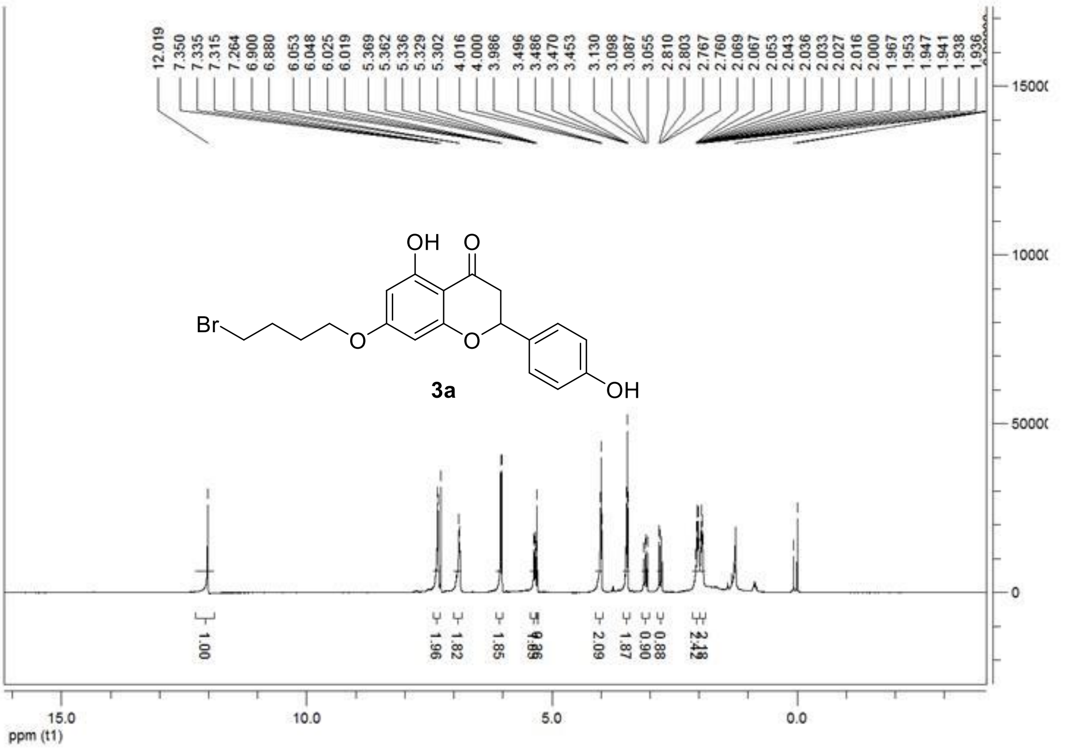
12.019
7.350
7.335
7.315
7.264
6.900
6.880
6.053
6.048
6.025
6.019
5.369
5.362
5.336
5.329
5.302
4.016
4.000
3.986
3.496
3.486
3.470
3.453
3.130
3.098
3.087
3.055
2.810
2.803
2.767
2.760
2.069
2.067
2.053
2.043
2.036
2.033
2.027
2.016
2.000
1.967
1.953
1.947
1.941
1.938
1.936
OH
O
Br
O
O
OH
3a
1.00
1.96
1.82
1.85
2.09
1.87
0.90
0.88
15.0
10.0
5.0
0.0
ppm (t1)

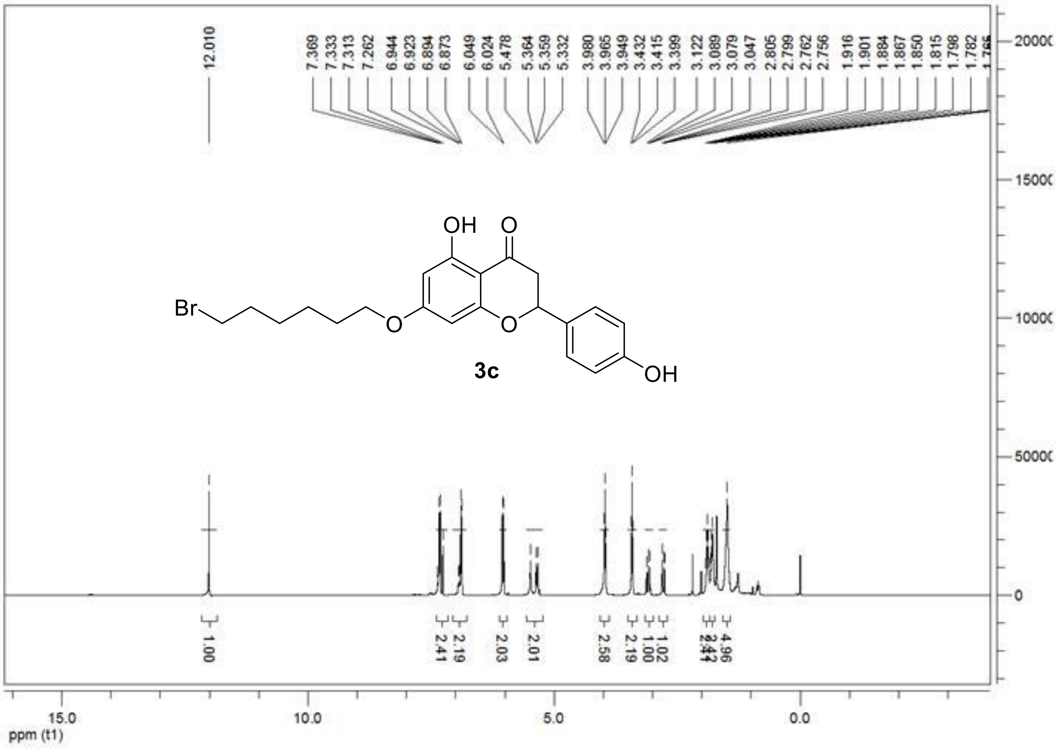
12.010
7.369
7.333
7.313
7.262
6.944
6.923
6.894
6.873
6.049
6.024
5.478
5.364
5.359
5.332
3.980
3.965
3.949
3.432
3.415
3.399
3.122
3.089
3.079
3.047
2.805
2.799
2.762
2.756
1.916
1.901
1.884
1.867
1.850
1.815
1.798
1.782
OH
O
Br
O
O
OH
3c
1.00
2.41
2.19
2.03
2.01
2.58
2.19
1.00
1.02
4.96
15.0
10.0
5.0
0.0
ppm (t1)

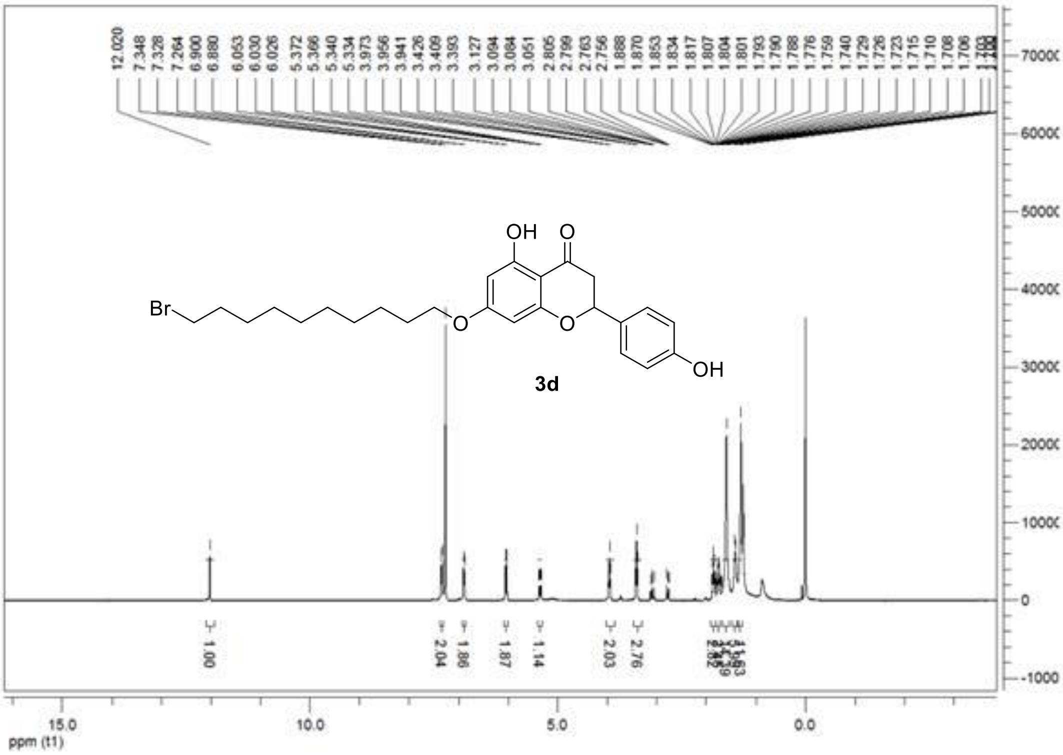
Br
OH
O
OH
3d
15.0
10.0
5.0
0.0
ppm (t1)
1.00
2.04
1.86
1.87
1.14
2.03
2.76

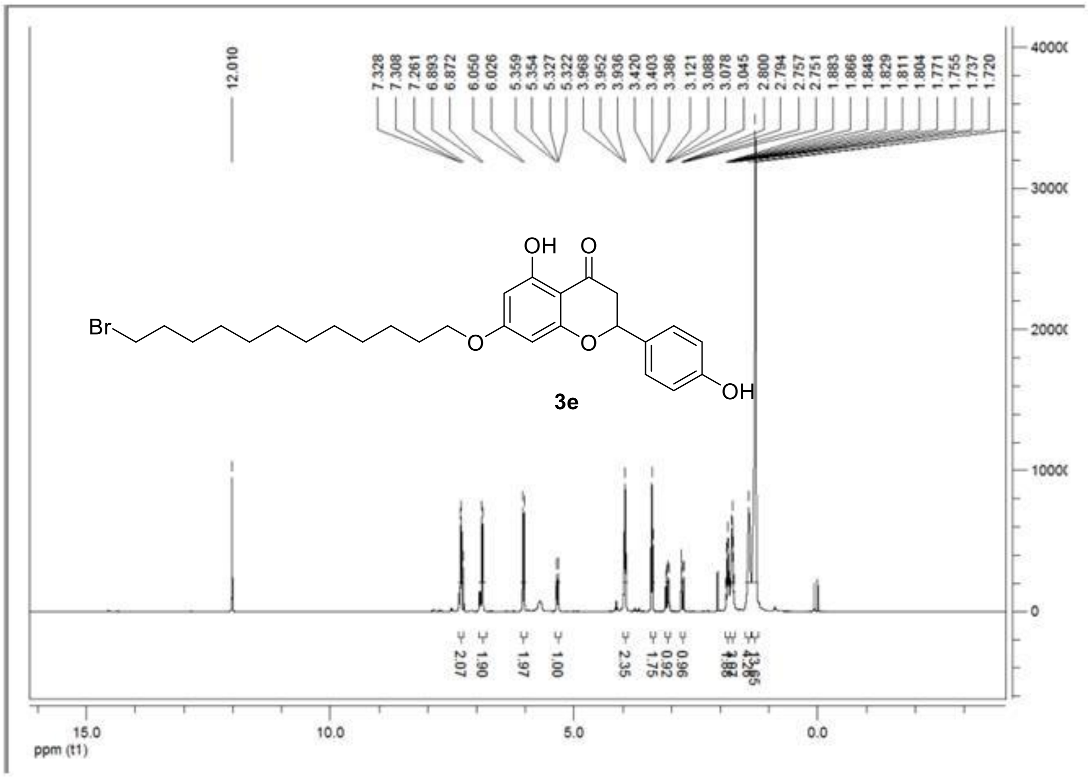
Br
OH
O
OH
3e
12.010
15.0
10.0
5.0
0.0
ppm (t1)
2.07
1.90
1.97
1.00
2.35
1.75
0.92
0.96

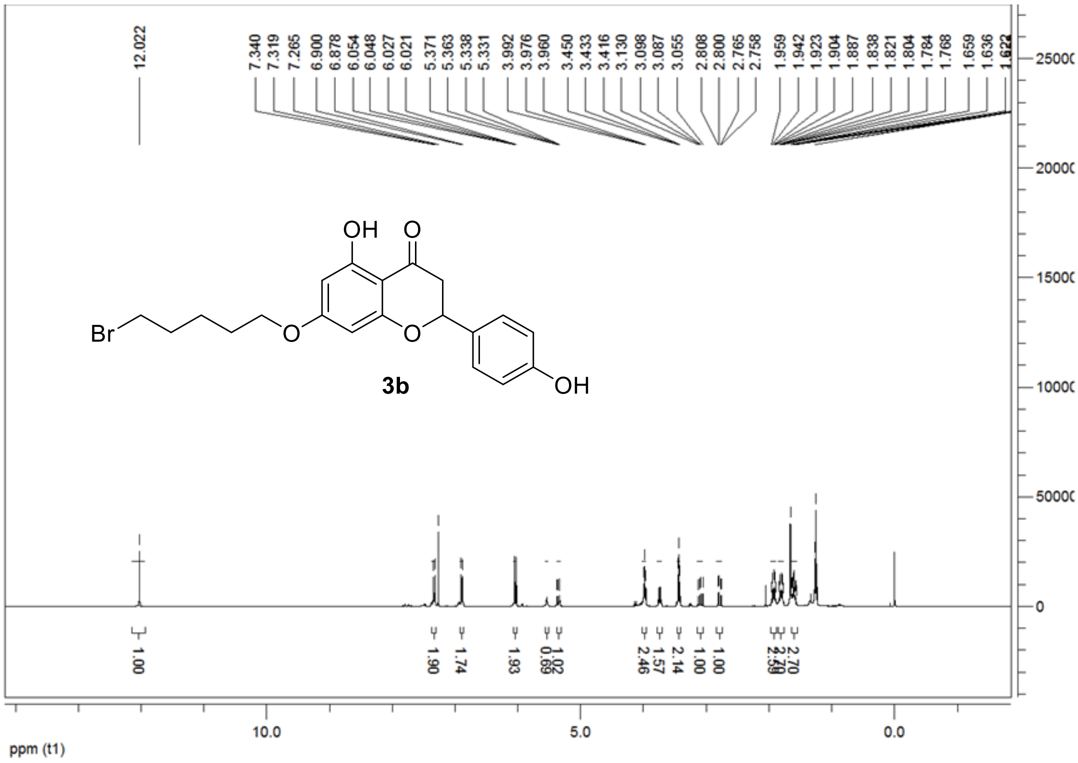
OH
O
Br
O
O
OH
3b
ppm (t1)

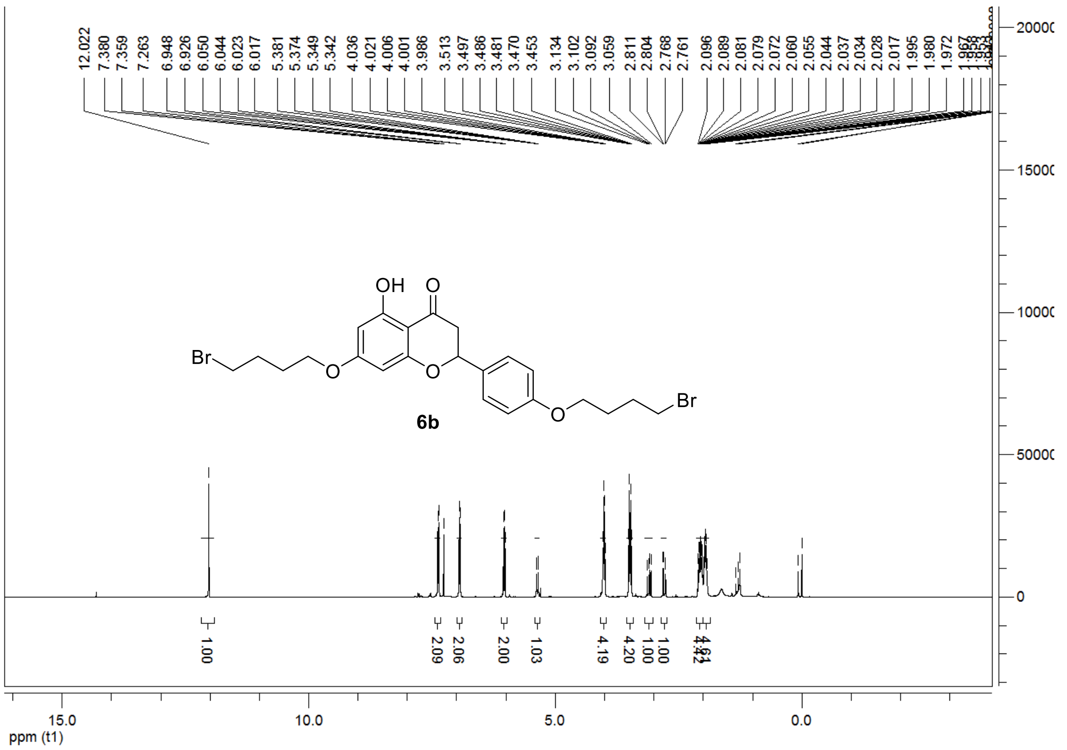
OH
O
Br
O
O
O
Br
6b
ppm (t1)

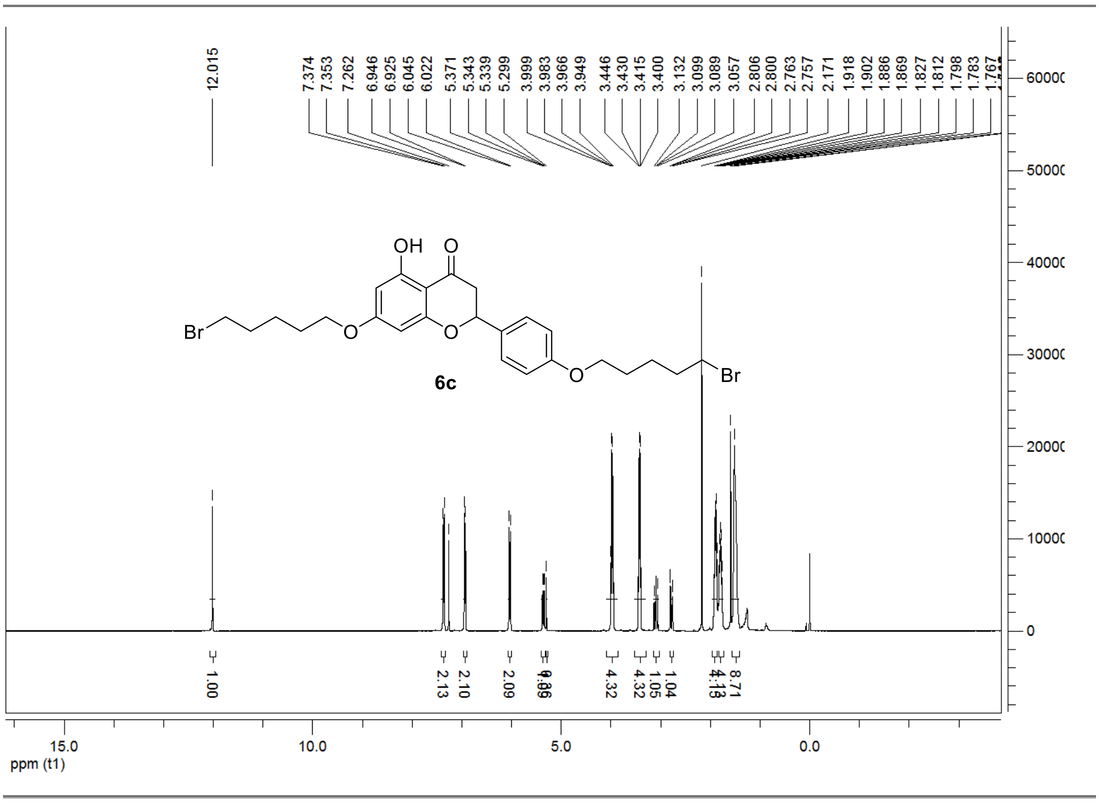
12.015
7.374
7.353
7.262
6.946
6.925
6.045
6.022
5.371
5.343
5.339
5.299
3.999
3.983
3.966
3.949
3.446
3.430
3.415
3.400
3.132
3.099
3.089
3.057
2.806
2.800
2.763
2.757
2.171
1.918
1.902
1.886
1.869
1.827
1.812
1.798
1.783
OH
O
Br
O
O
O
Br
6c
1.00
2.13
2.10
2.09
4.32
4.32
1.05
1.04
8.71
15.0
10.0
5.0
0.0
ppm (t1)

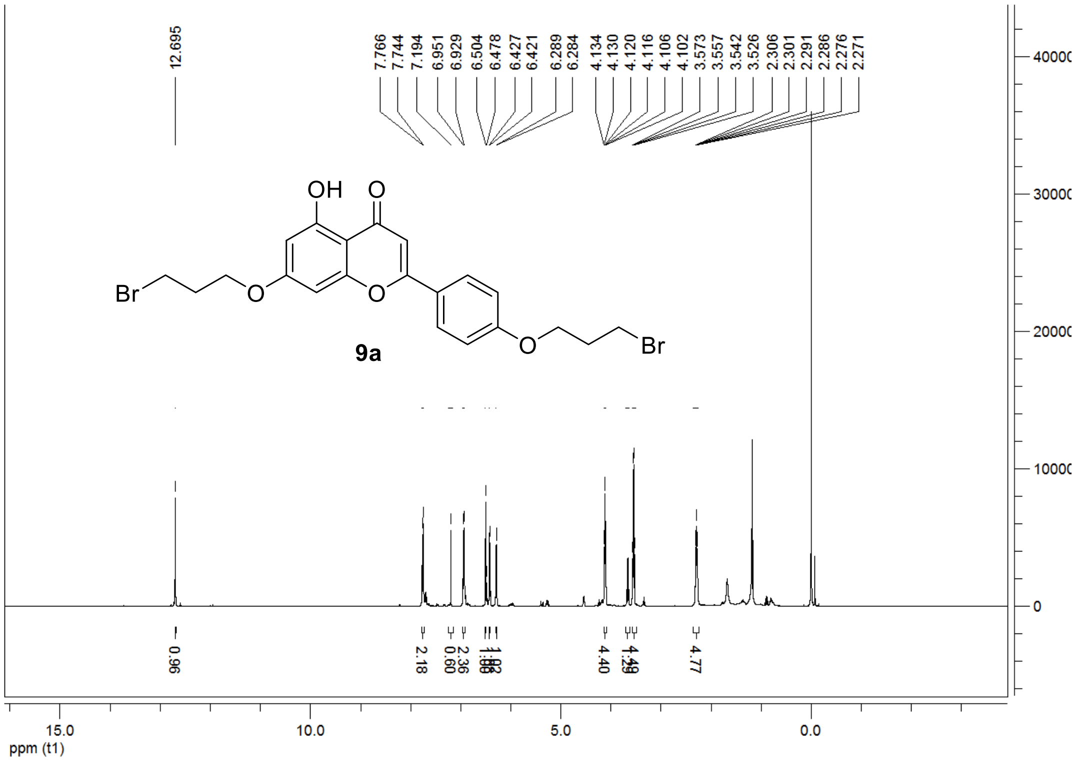
12.695
7.766
7.744
7.194
6.951
6.929
6.504
6.478
6.427
6.421
6.289
6.284
4.134
4.130
4.120
4.116
4.106
4.102
3.573
3.557
3.542
3.526
2.306
2.301
2.291
2.286
2.276
2.271
OH
O
Br
O
O
O
Br
9a
0.96
2.18
0.60
2.36
4.40
4.77
15.0
10.0
5.0
0.0
ppm (t1)

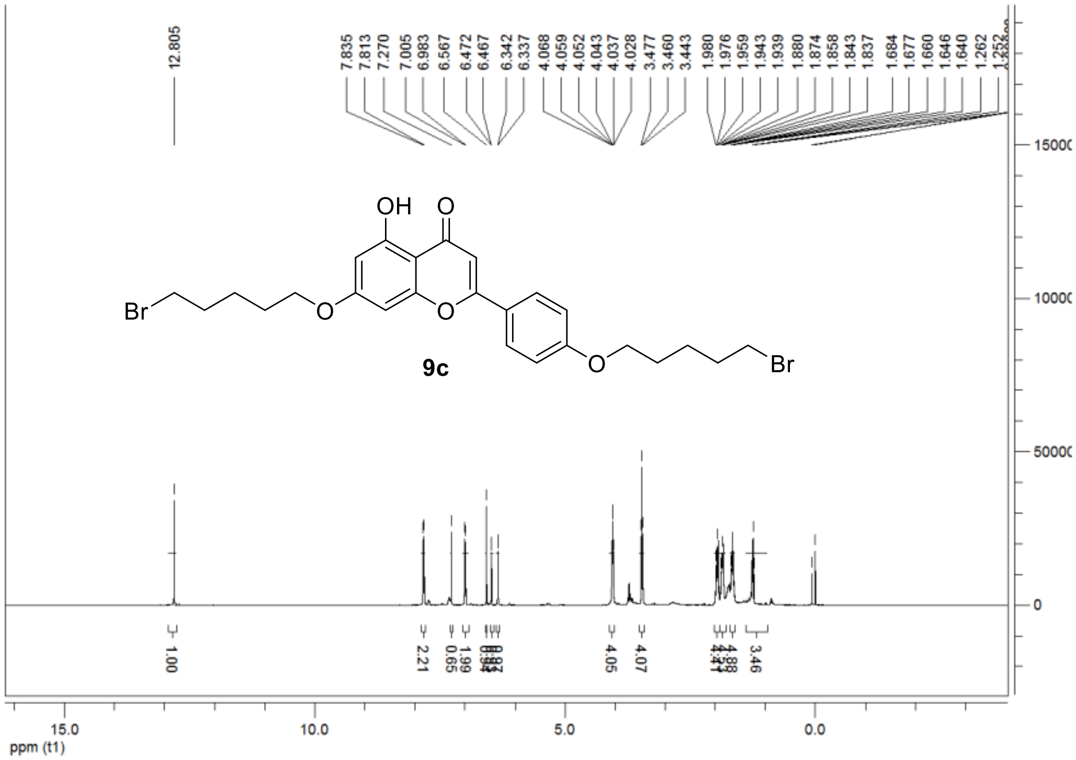
12.805
7.835 7.813 7.270 7.005 6.983 6.567 6.472 6.467 6.342 6.337 4.068 4.059 4.052 4.043 4.037 4.028 3.477 3.460 3.443 1.980 1.976 1.959 1.943 1.939 1.880 1.874 1.858 1.843 1.837 1.684 1.677 1.660 1.646 1.640 1.262 1.252
OH
O
Br
9c
1.00
2.21
0.65
1.99
4.05
4.07
4.88
3.46
15.0
10.0
5.0
0.0
ppm (t1)
15000
10000
50000
0

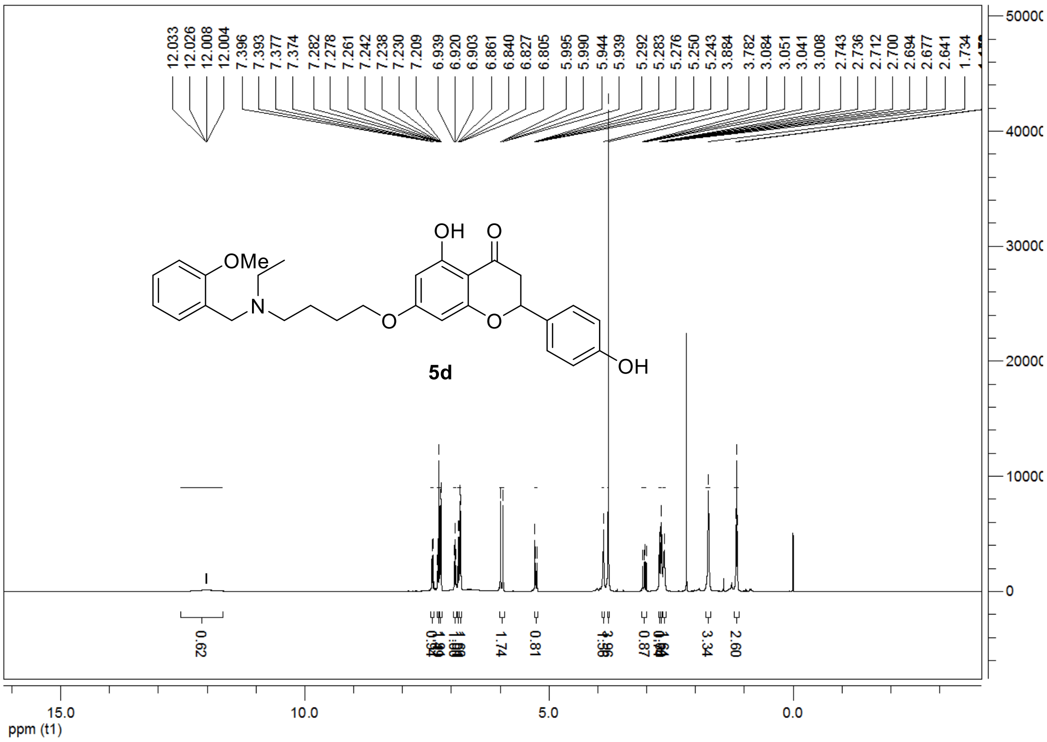
12.033 12.026 12.008 12.004 7.396 7.383 7.377 7.374 7.282 7.278 7.261 7.242 7.238 7.230 7.209 6.939 6.920 6.903 6.861 6.840 6.827 6.805 5.995 5.990 5.944 5.939 5.292 5.283 5.276 5.250 5.243 3.884 3.782 3.084 3.051 3.041 3.008 2.743 2.736 2.712 2.700 2.694 2.677 2.641 1.734
OH
O
OMe
N
OH
5d
0.62
1.74
0.81
0.87
3.34
2.60
15.0
10.0
5.0
0.0
ppm (t1)
50000
40000
30000
20000
10000
0

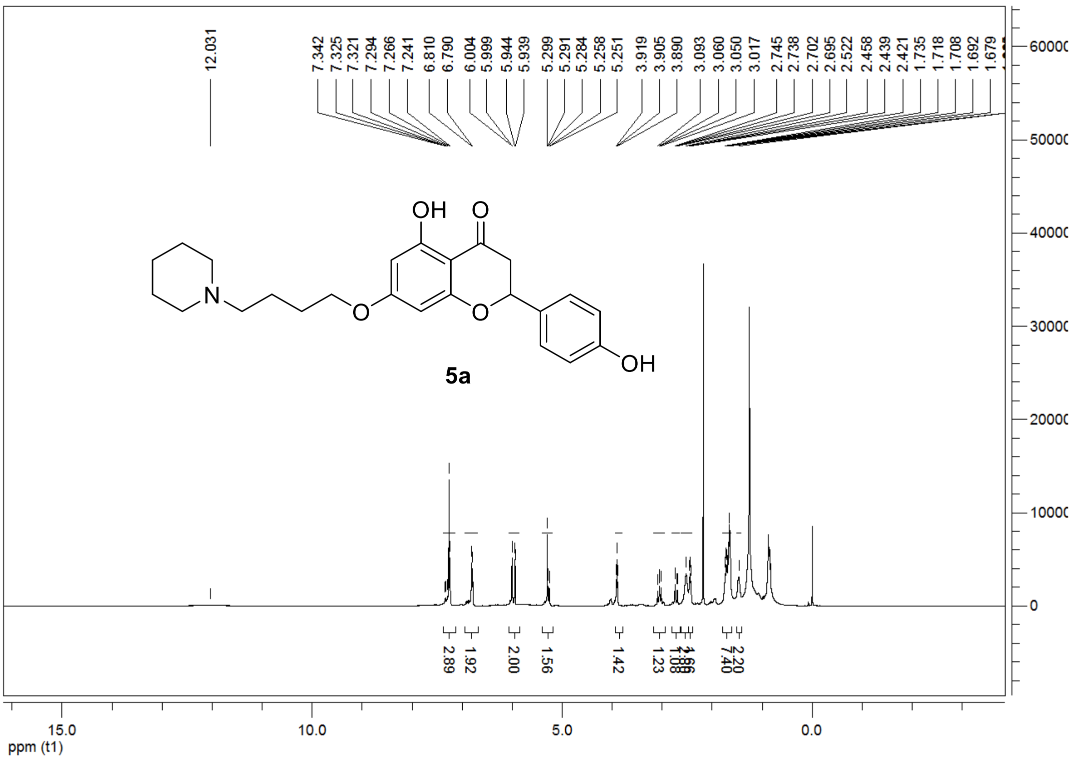
5a
ppm (t1)

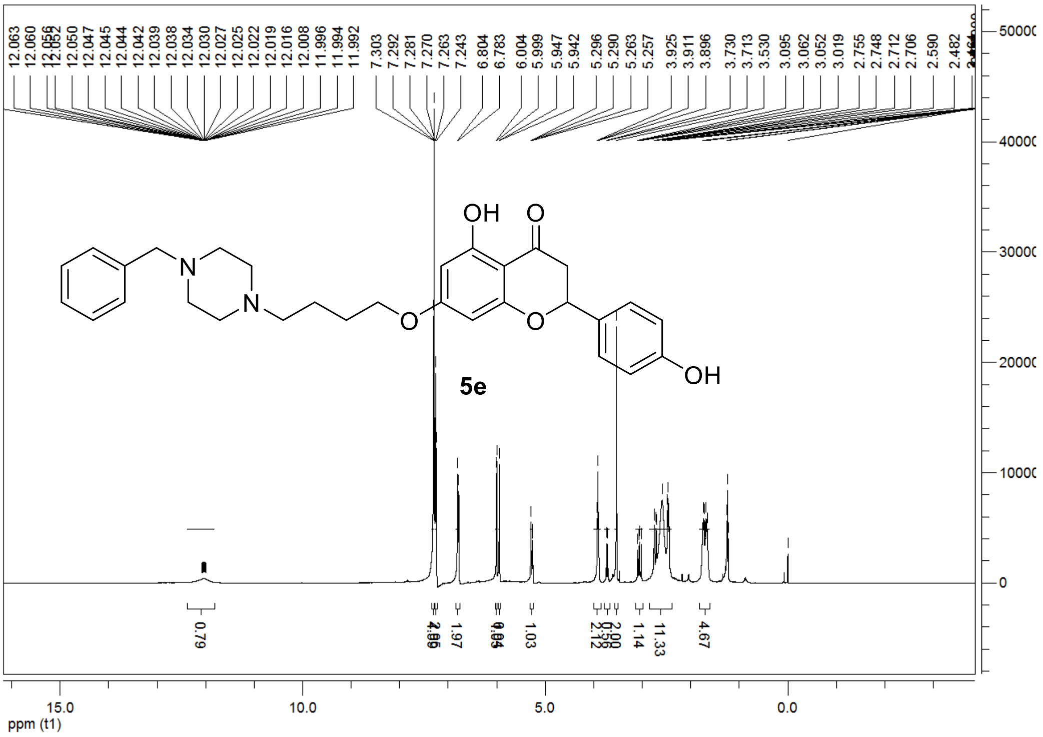
5e
ppm (t1)

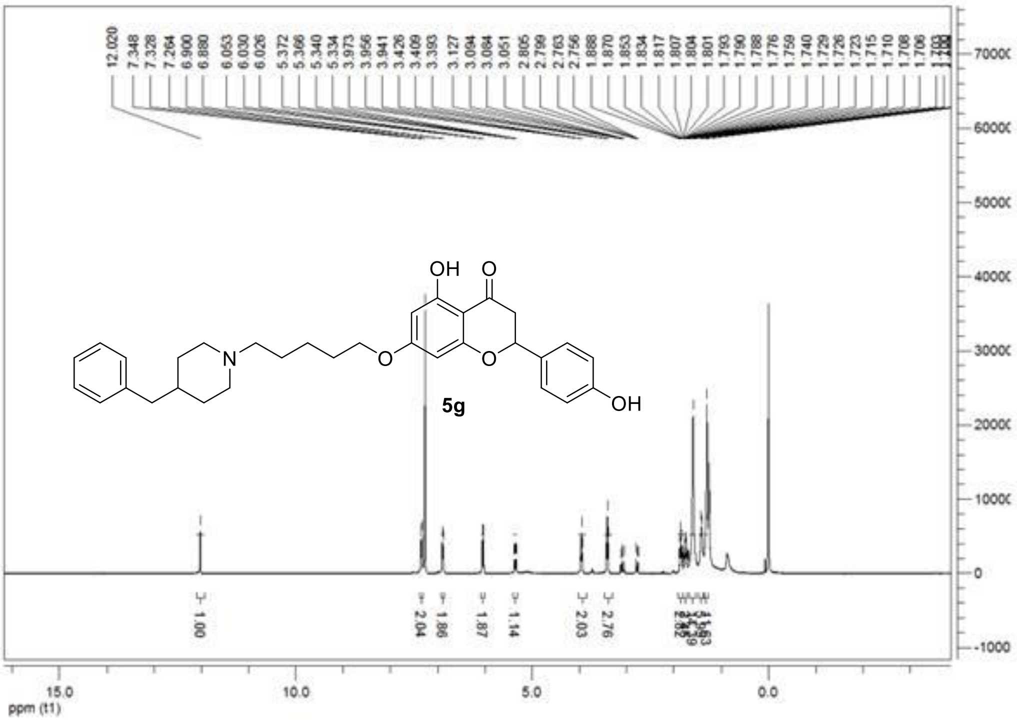

5g

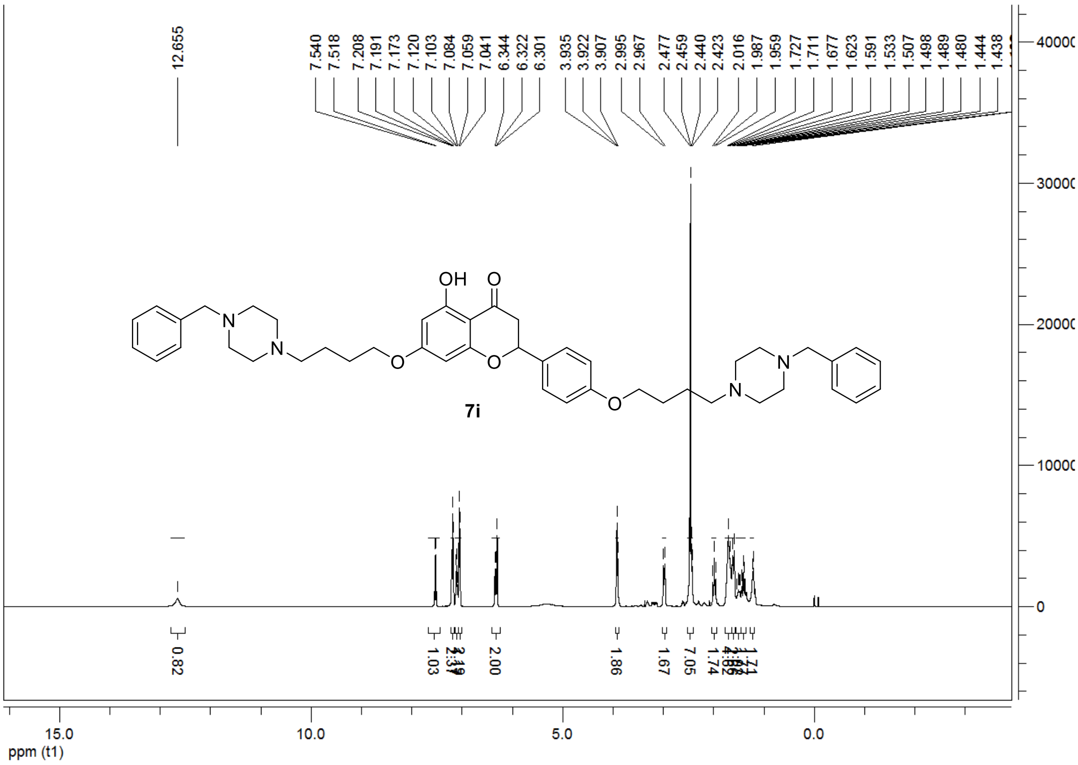

7i

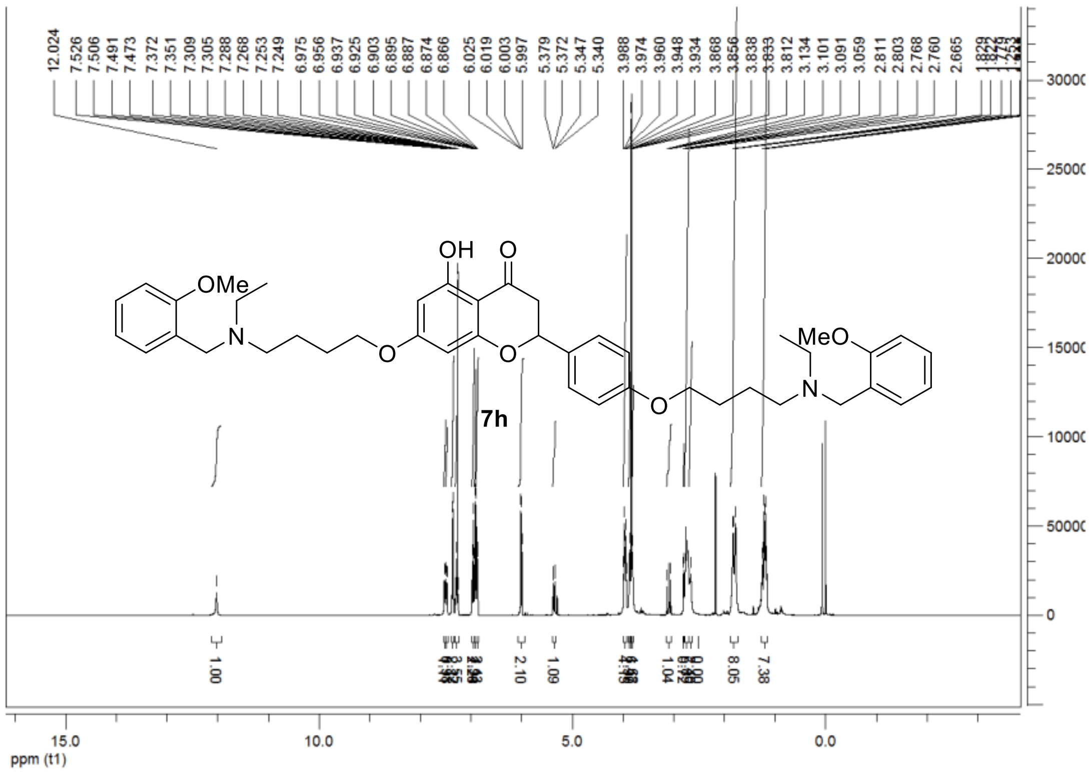
OH
O
OMe
N
O
O
7h
MeO
O
N
15.0
10.0
5.0
0.0
ppm (t1)

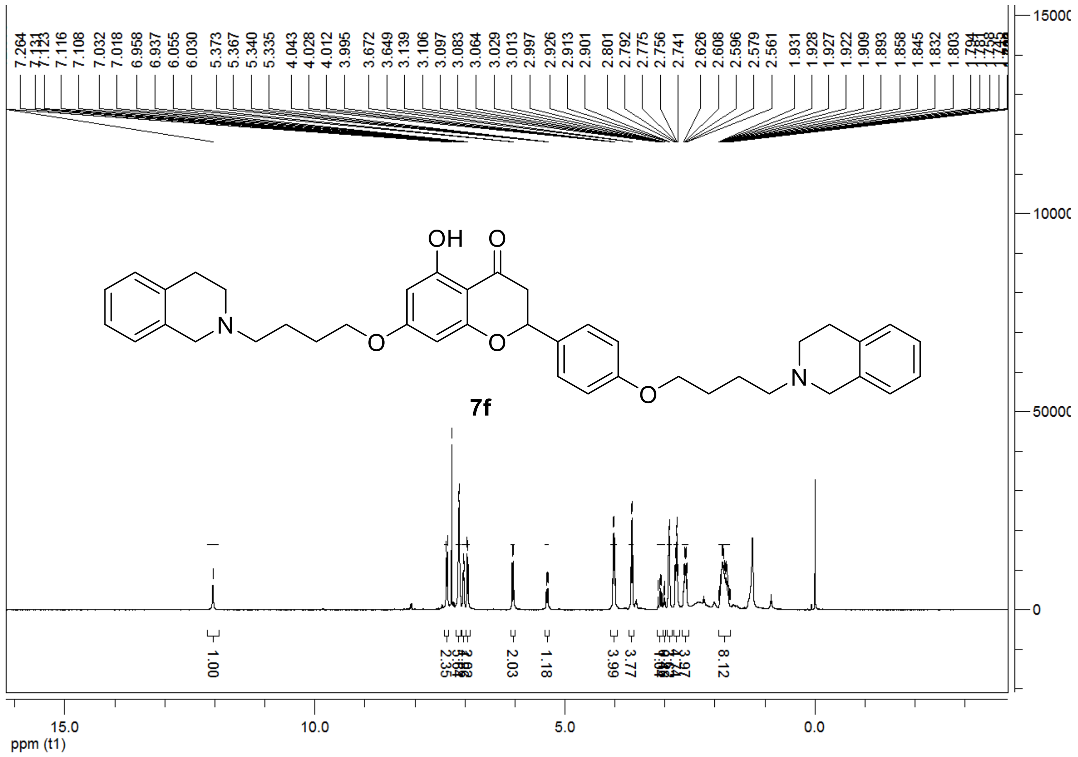
OH
O
N
O
O
7f
O
N
15.0
10.0
5.0
0.0
ppm (t1)
